# A scoping review on the field validation and implementation of rapid diagnostic tests for vector-borne and other infectious diseases of poverty in urban areas

**DOI:** 10.1186/s40249-018-0474-8

**Published:** 2018-09-03

**Authors:** Lyda Osorio, Jonny Alejandro Garcia, Luis Gabriel Parra, Victor Garcia, Laura Torres, Stéphanie Degroote, Valéry Ridde

**Affiliations:** 1 Epidemiology and Population Health Research Group, School of Public Health, Universidad del Valle, Calle 4B No. 36-00 Edif 118 Escuela de Salud Pública, Universidad del Valle Campus San Fernando, Cali, Colombia; 2 School of Medicine, Universidad del Valle, Cali, Colombia; 3 University of Montreal Public Health Research Institute (IRSPUM), Montreal, Canada; 4 French Institute for Research on Sustainable Development (IRD), Paris Descartes University, Population and Development Center (CEPED), Université Paris Sorbonne Cité, National Institute of Health and Medical Research (INSERM), Health, Vulnerabilities and Gender Relations South (SAGESUD), Paris, France

**Keywords:** Communicable diseases, Diagnostic services, Point-of-care testing, Field evaluation, Sensitivity and specificity, Implementation, Evaluation studies, Urban health

## Abstract

**Background:**

Health personnel face challenges in diagnosing vector-borne and other diseases of poverty in urban settings. There is a need to know what rapid diagnostic technologies are available, have been properly assessed, and are being implemented to improve control of these diseases in the urban context. This paper characterizes evidence on the field validation and implementation in urban areas of rapid diagnostics for vector-borne diseases and other diseases of poverty.

**Main body:**

A scoping review was conducted. Peer-reviewed and grey literature were searched using terms describing the targeted infectious diseases, diagnostics evaluations, rapid tests, and urban setting. The review was limited to studies published between 2000 and 2016 in English, Spanish, French, and Portuguese. Inclusion and exclusion criteria were refined post hoc to identify relevant literature regardless of study design and geography.

A total of 179 documents of the 7806 initially screened were included in the analysis. Malaria (*n =* 100) and tuberculosis (*n =* 47) accounted for the majority of studies that reported diagnostics performance, impact, and implementation outcomes. Fewer studies, assessing mainly performance, were identified for visceral leishmaniasis (*n =* 9), filariasis and leptospirosis (each *n =* 5), enteric fever and schistosomiasis (each *n =* 3), dengue and leprosy (each *n =* 2), and Chagas disease, human African trypanosomiasis, and cholera (each *n =* 1). Reported sensitivity of rapid tests was variable depending on several factors. Overall, specificities were high (> 80%), except for schistosomiasis and cholera. Impact and implementation outcomes, mainly acceptability and cost, followed by adoption, feasibility, and sustainability of rapid tests are being evaluated in the field. Challenges to implementing rapid tests range from cultural to technical and administrative issues.

**Conclusions:**

Rapid diagnostic tests for vector-borne and other diseases of poverty are being used in the urban context with demonstrated impact on case detection. However, most evidence comes from malaria rapid diagnostics, with variable results. While rapid tests for tuberculosis and visceral leishmaniasis require further implementation studies, more evidence on performance of current tests or development of new alternatives is needed for dengue, Chagas disease, filariasis, leptospirosis, enteric fever, human African trypanosomiasis, schistosomiasis and cholera.

**Electronic supplementary material:**

The online version of this article (10.1186/s40249-018-0474-8) contains supplementary material, which is available to authorized users.

## Multilingual abstracts

Please see Additional file 1 for translations of the abstract into six official working languages of the United Nations.

## Background

A common scenario in health facilities in urban areas involves the following diagnostic process: a patient’s arrival, triage, questionnaire and physical examination, presumptive diagnosis, request for laboratory analyses, taking of the sample, its transport to the laboratory, its processing, transmission of result, review by the treating physician, and case management decision. This process is likely to have varying turnaround times, some too long to be acceptable in relation to the expected needs of the patient, the physician, and the health system. For infectious diseases, there is a recognized need for speed in this process, as timely correct treatment can improve patients’ probability of survival and prevent long-term complications and further dissemination. Consequently, rapid diagnostics are being developed using a wide spectrum of technological platforms, including rapid microscopy, immunochromatography (lateral flow, dipstick, card, latex), molecular technologies (real-time polymerase chain reaction [PCR], arrays, mass spectrometry, nanotechnology), and microfluidics [1]. In 2004, a call was made to improve validation and prioritize research and development of rapid diagnostics for tropical infectious diseases in developing countries as a key element in case management both within and outside hospital settings, in public health surveillance, and in meeting world-wide control and elimination targets [2].

Research and development of rapid diagnostics for vector-borne diseases (VBDs) and other poverty-related infectious diseases have been hampered by lack of investment, weak and heterogeneous regulatory standards, and insufficient capacity for product development in endemic countries. Moreover, the clinical and public health impacts that available rapid diagnostics might have are impeded by the scant adequate evidence of their accuracy and implementation processes under real-life conditions in the various settings where these diseases occur [3]. These include urban settings where social and environmental determinants facilitate the emergence, re-emergence and dissemination of infectious diseases [4]. Hence, the objective of the present study was to summarize the evidence on field validation and implementation in urban areas of rapid diagnostics for VBDs and other infectious diseases of poverty to inform decision-makers and future research. This is part of a series of scoping reviews on urban health and VBDs.

## Methods

### Description of the Delphi process used to select the topic of the scoping review

To decide on topics for scoping reviews on urban health and VBDs, we used an eDelphi survey to select the six topics considered of highest priority by a panel of 109 international experts (43% researchers; 52% public health decision-makers; 5% from the private sector). The eDelphi process consisted of three rounds: 1) participants suggested topics to consider; 2) the more than 80 topics suggested were rated from “1–eliminate” to “5–top priority”; and finally, 3) the 20 topics rated 4 or 5 by more than 65% of panelists were rated a second time. The present topic was the only one automatically retained at the end of the second round, have obtained the mean rating of 4.29 ± 0.87 and thereby being ranked first (rated 4 or 5 by 85.7% of panelists).

### Search strategy

The search strategy was constructed to answer the research questions of what rapid diagnostic tests for VBD and other infectious diseases of poverty in urban areas have been evaluated and what those evaluations were and found [5]. Search terms were defined that described four key concepts: 1) VBDs and other infectious diseases; 2) urban area; 3) diagnostic technologies; and 4) characteristics of rapid diagnostic technologies, these terms were combined using Boolean operators OR (within key concepts) and AND (between key concepts). The search was conducted in the following databases: MEDLINE (PubMed), Cochrane Library (Wiley), EMBASE, LILACS, Global Health (Ovid), WHOLIS, Opengray, and Scopus (Additional file 2: Table S1: Search strategy). Additional information was identified by manually screening the references of retrieved literature reviews and of some of the articles retained, as well as known international diagnostic programs, such as the World Health Organization (WHO) in vitro diagnostics prequalification process and the Special Programme for Research and Training in Tropical Diseases (TDR) diagnostic evaluation series [6, 7].

### Study selection

The retrieved literature was downloaded into Zotero reference manager, and duplicates were identified and deleted. The entire library was exported to a Microsoft Excel® (2016, Microsoft, Redmond, Washington, United States) screening template adapted from a free systematic review template [8]. References were distributed to two teams of two members each (JG/LGP and LT/VG), who independently reviewed titles and abstracts. During a pilot exercise, satisfactory agreement for the screening process was assessed between reviewers of the same team and between teams. A third independent reviewer (LO), who also performed the full-text screening, resolved discordant results. The study selection process was iterative. Studies published between 2000 and 2016 (last search conducted on October 31, 2016) in English, Spanish, French, and Portuguese were included. The following exclusion criteria were applied to titles, abstracts, and full texts: not relating to infectious diseases; none of our targeted infectious diseases; not conducted in humans or on human samples; not conducted in low- and middle-income countries (LMICs), or, if in a high-income country, not poverty related; no diagnosis of the disease/infection (e.g. diagnosis of complications or drug resistance); is a book; is in a rural area; diagnostics were used but not evaluated (e.g. to measure prevalence of disease/infection); the diagnostic intervention was not a biomarker (e.g. clinical algorithm or X-rays); it was not a field evaluation. The inclusion and exclusion criteria for rapid tests and urban area were refined post hoc during full-text screening to ensure objective definitions were followed. First, rapid diagnostic tests were defined using the WHO criteria [9]. One exception was made to include a rapid automated nucleic acid amplification test for tuberculosis (Xpert® MTB/RIF), as it is endorsed by WHO [10]. Second, it was not feasible to apply a standard definition of urban area, because what constitutes an urban population differs between countries and even within countries over time [11], and details of study settings in the retrieved documents were not consistent. Instead, we excluded studies only when they explicitly mentioned that the study area was exclusively rural or remote, or if they described the population as farmers, tribal, or nomadic. Otherwise, the study was included.

### Data extraction and analysis

Characteristics, diagnostic performance, and implementation outcomes were extracted into a template using a Microsoft Excel® spreadsheet. The following tools were used for data extraction: 1) Mixed Methods Appraisal Tool (MMAT) to describe key characteristics of qualitative, quantitative (randomized controlled, non-randomized, and descriptive), and mixed-methods studies [12]; 2) Template for Intervention Description and Replication (TIDieR) to describe the diagnostic interventions [13]; 3) Analysis of the transferability of health promotion interventions (ASTAIRE) to describe the epidemiological and sociodemographic characteristics of the study population [14]; and 4) diagnostic impact of tests, performance (sensitivity, specificity, predictive values), and implementation outcomes (acceptability, adoption, appropriateness, feasibility, fidelity, cost, penetration, and sustainability) [15] (Table 1). A descriptive analysis was done by disease.

**Table 1:** Definitions of diagnostic impact, performance, and implementation outcomes

Impact: effect of diagnostic test implementation on public health or patient-oriented outcomes	
Performance: operational characteristics of diagnostic tests in relation to sensitivity, specificity, predictive values, and concordance with other tests	
Acceptability: patient’s and provider’s perceptions of a diagnostic test being satisfactory	
Adoption: intention, decision, or action to use a diagnostic test intervention	
Appropriateness: perception of how well the diagnostic intervention meets the needs in a specific context	
Feasibility: the extent to which the diagnostic intervention can be successfully used in a specific context	
Fidelity: to what degree the diagnostic intervention was implemented as originally planned	
Cost: monetary effort of the use of a diagnostic intervention in a specific context	
Penetration: to what extent the diagnostic intervention reached the expected users	
Sustainability: to what extent the diagnostic intervention is maintained or institutionalized	

## Results

### Characteristics of included studies

**Fig. 1:**
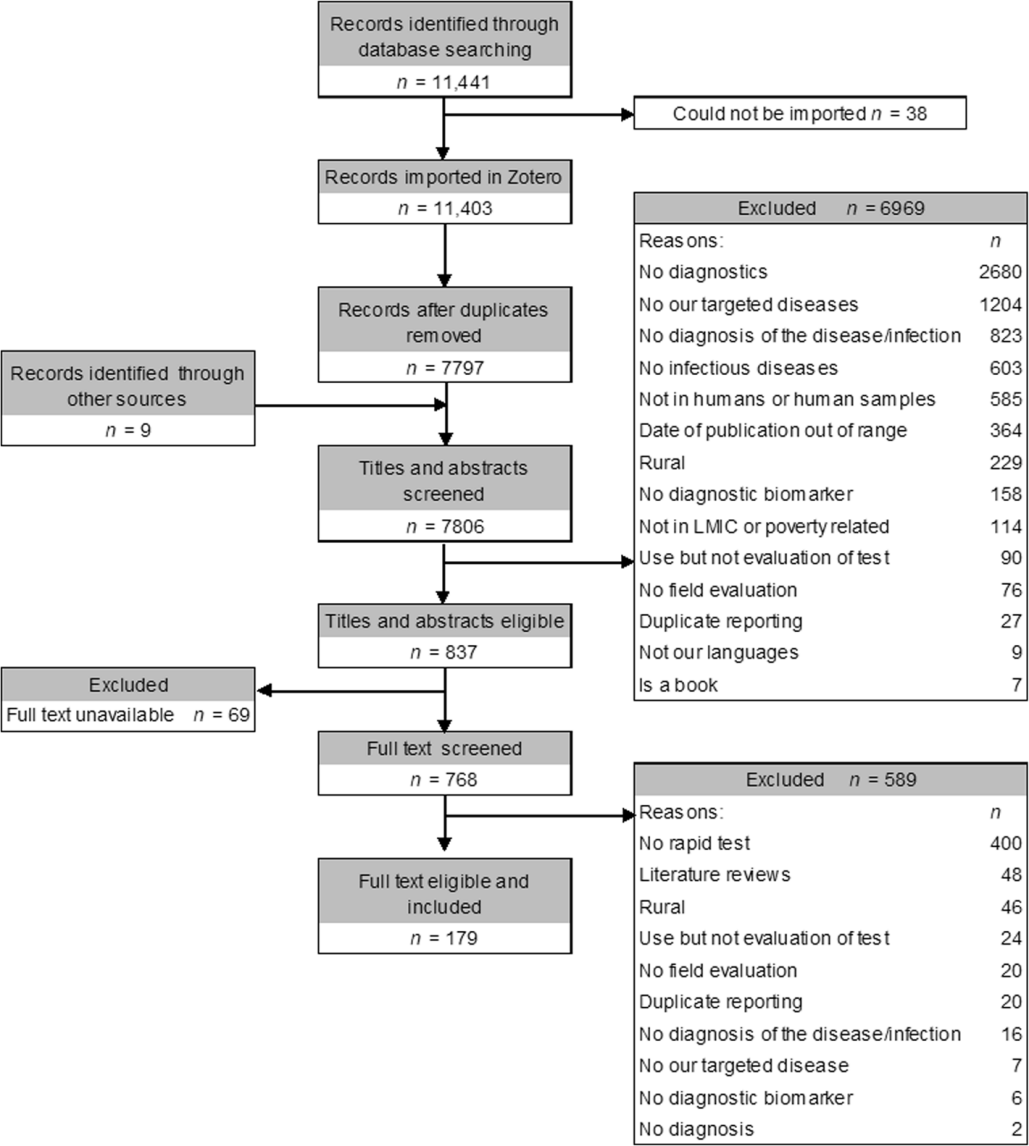
Flow chart of included studies

Of the 11 441 documents that were identified from all databases plus 9 from other sources, 7806 were screened after duplicates were removed. From these, 6969 were excluded during titles/abstracts screening, 589 were excluded during full-text screening and 69 full texts could not be located. Hence, a total of 179 documents were included in the analysis, of which 143 were published in peer-reviewed journals, 34 were conference abstracts, and two were Master’s theses (Fig. 1).

**Fig. 2:**
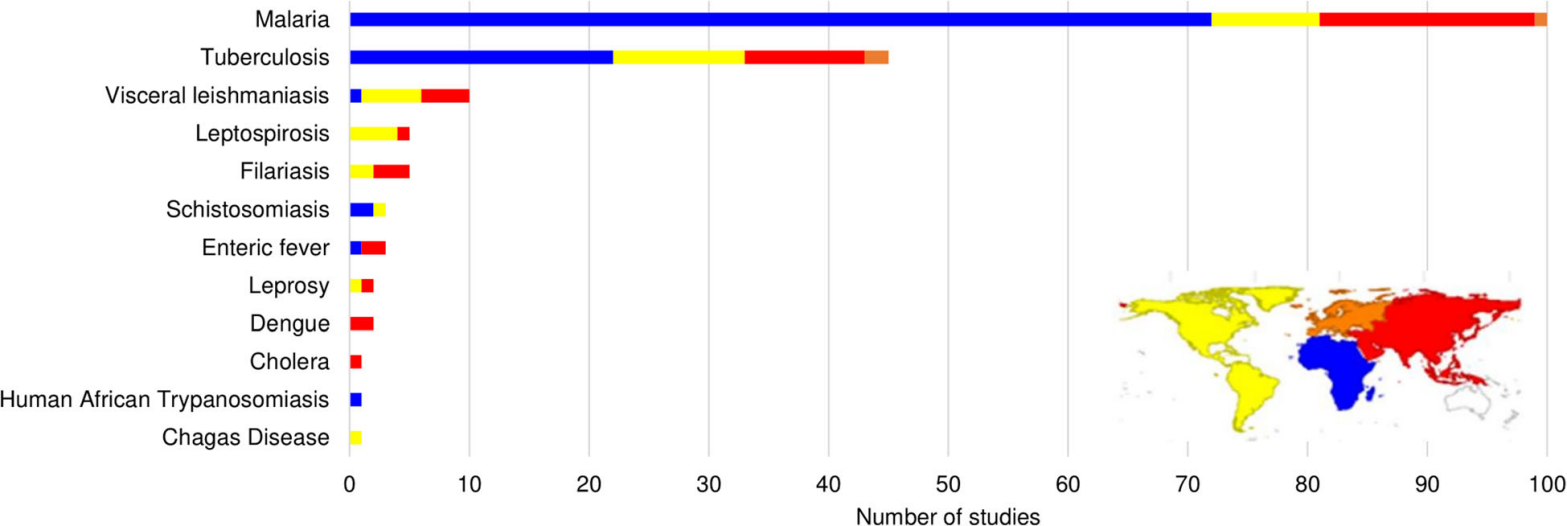
Number of included studies by disease and continent

The majority of studies were about diagnosis of malaria (*n =* 100, 56%) and tuberculosis (*n =* 47, 26%), followed by visceral leishmaniasis (*n =* 9, 5%), filariasis and leptospirosis (*n =* 5 each, 3%), enteric fever and schistosomiasis (*n =* 3 each, 1.7%), dengue and leprosy (*n =* 2 each, 1%), and Chagas disease, African trypanosomiasis, and cholera (*n =* 1 each, 0.6%). More than half of the studies were carried out in Africa (*n =* 99, 55%), followed by Asia (*n =* 41, 23%), the Americas (*n =* 34, 19%) and Europe (*n =* 3, 2%). One study was conducted in countries on three continents (Africa, Americas, and Asia), and one study did not provide information (Fig. 2).

**Fig. 3:**
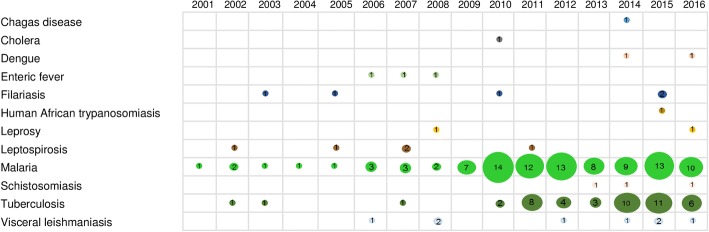
Frequency of studies by disease and year of publication

There was an increasing trend in the number of studies over time, with 83% (*n =* 150) of the studies published after 2009. This trend was particularly observed for malaria (from 2009 onwards), tuberculosis (from 2010 onwards), and visceral leishmaniasis (from 2012 onwards) (Fig. 3).

All but six of the included studies were considered to have clear objectives. However, the reporting of the diagnostic intervention was often incomplete, particularly in relation to the level of training of the person performing the tests, which was not reported in most studies (*n =* 104, 58%). Likewise, epidemiological and sociodemographic characteristics of the population were not described in 27 (15%) studies. Outcomes of evaluation of rapid diagnostics were mainly reported in relation to their performance alone (*n =* 112, 62.5%), while impact was evaluated alone in 12 studies (6.7%) and together with performance in six studies (3.4%). Cost and acceptability were the next characteristics evaluated either alone or in combination. There was no evaluation of fidelity or penetration. For every disease, there was at least one study that evaluated the performance of a test (Table 2).

**Table 2:** Frequency of outcome evaluated by disease

Outcome	Malaria	Tuberculosis	Visceral Leishmaniasis	Filariasis	Leptospirosis	Enteric Fever	Schistosomiasis	Dengue	Leprosy	Chagas Disease	Cholera	Trypanosomiasis	Total
Acceptability	2	2	1						1				6
Acceptability and adoption	2												2
Acceptability and appropriateness	1												1
Acceptability and feasibility	2												2
Acceptability, adoption and appropriateness	1												1
Acceptability, appropriateness and feasibility	1												1
Adoption	6	1											7
Cost	6	2	1										9
Details of study design	1												1
Feasibility		1											1
Impact	5	7											12
Impact and adoption	1												1
Impact and acceptability	2												1
Impact and cost	1												1
Impact and performance	3	2							1				6
Impact, acceptability, and appropriateness	1												1
Impact, acceptability, adoption and sustainability	1												1
Impact, acceptability and sustainability	1												1
Impact, adoption and appropriateness	1												1
Impact, adoption and penetration		1											1
Impact, performance and acceptability	1												1
Impact, performance and cost	1												1
Lessons learned	1												1
Other use	1												1
Performance	55	30	7	4	5	3	3	2		1	1	1	112
Performance and cost	2	1											3
Performance, acceptability and appropriateness				1									1
Performance and appropriateness	1												1
Total	100	47	9	5	5	3	3	2	2	1	1	1	179

Reference standards used to assess the performance of diagnostic tests were reported in all but 6 studies. Light microscopy was the diagnostic reference standard most frequently used in malaria and filariasis. For malaria, microscopy alone was used in 37 studies, and together with PCR in 12 studies. All four filariasis studies used microscopy as reference standard. More variability in the standards used was observed in tuberculosis, visceral leishmaniasis, leptospirosis, enteric fever and schistosomiasis. For tuberculosis, liquid culture alone (*n =* 7) was the most common reference test used followed by the combination of microscopy plus culture (*n =* 6). For visceral leishmaniasis, the combination of bone marrow microscopy and direct agglutination test was used in 2 studies. For leptospirosis, the microscopy agglutination test plus culture, and IgM enzyme-linked immunosorbent assay (ELISA) alone were used each in two studies. There was one study using the following reference tests: Culture alone or Widal test alone for enteric fever; and Kato-Katz alone, Kato-Katz plus ELISA, or urine microscopy for schistosomiasis. RTPCR was used for dengue, a combination of ELISA plus indirect haemagglutination (IHA) plus immunofluorescent antibody assay for Chagas disease, and culture for cholera (Additional file 3: Table S2: Details of included studies).

### Malaria

There were 100 studies on malaria, of which 63 assessed performance of rapid tests. Among the evaluated assays were: OptiMAL-IT (DiaMed Basel, Switzerland, or DiaMed China Ltd., Hong Kong, China, or Flow Inc., Portland, OR, USA); Now ICT Malaria for Pf or Pf/Pv, ParaSight-F, Paracheck-Pf (Orchid Biomedical Systems, Verna, Goa, India), CareStart Pf/Pv, SD Bioline Malaria Pf/Pan, Paramax-3 Pan/Pv/Pf, DiaSpot® malaria (Acumen Diagnostics Inc., USA), Mal Card™, ICT Malaria Combo (ICT Diagnostics, Cape Town, South Africa), ParaHIT f (Span Diagnostics, Surat, India), PALUTOP+ 4 (All.Diag, Strasbourg, France), ICT malaria (Healgen Scientific LCC, Houston, TX, USA), ICT Parascreen test kit (Zephyr Biomedicals, Verna, Goa, India), ICT P.f./P.v™ (ICT; AMRAD–ICT, Brookvale, Australia), and Malaria Makromed (Makromed (Pty) Ltd., Johannesburg, South Africa). Reporting sensitivities and specificities were highly variable (from 2 to 100% and from 44.3 to 100%, respectively), depending on study site, reference standard used, assay, parasitaemia, *Plasmodium* species (*P. falciparum, P. vivax and P. knowlesi*), whether hospital or community-based study, age of patients, and pregnancy (i.e., placental malaria) [16–78].

Impact was evaluated in 18 studies that reported that antimalarials were used in both rapid tests positive and negative patients; however, there was a decrease in use of antimalarials for presumptive treatment. Reductions in incidence of confirmed cases were observed plus a twofold or fourfold decrease in antimalarial prescription [17, 23, 59, 63, 79–91].

Acceptability was measured in 15 studies that showed a wide spectrum of responses to the use of rapid tests. In some studies, rapid tests were valued and appreciated by health workers, drug shop vendors, and communities, who in some cases were willing to purchase subsidized tests or trusted them, either because they were approved by relevant authorities and consistent with symptoms or because they were thought to reduce the cost of poor treatment. In contrast, in other scenarios, providers, health workers, and communities disliked or were suspicious of rapid tests. Among the reasons given for the low acceptability were: lack of trust in the accuracy of the tests (e.g. result clashes with patient’s or provider’s opinion); test positive after treatment; not able to quantify parasites; invalid results (control band non-reactive); high burden added to providers’ work; no supplies, including gloves; patients’ belief that they were being tested for HIV; or the cultural belief that the blood of someone alive cannot be buried. Interventions were designed to improve acceptability of rapid tests [23, 81, 83, 84, 89, 91–100].

Studies of the adoption of rapid tests (*n =* 11) identified an increased number of facilities using the tests. Routine direct and/or indirect quality assessment of performance was included as part of diagnostic tests’ adoption in some studies, but annual quality assurance assessments were found more often in the public than private sector. Problems with stock-outs of tests and paperwork were reported [84, 88, 95, 99–106].

There were 10 cost studies, of which nine presented results and one did not report the findings. The rapid test cost per correctly treated patient was found to be similar to the cost of microscopy, but lower than clinical diagnosis. The estimated cost of the rapid tests themselves ranged from USD 0.47 to 2.00, and the implementation cost per kit (excluding the test), from USD 0.04 to 0.05. It was reported that implementing rapid tests resulted in increased cost of treatment per patient in public facilities, despite drug savings in the health system. However, rapid tests were found to be more cost-effective than presumptive treatment in other studies, depending on sensitivity and costs of tests and prevalence of disease. Willingness to pay for rapid tests in Nigeria increased after they were tested [59, 60, 75, 86, 107–112].

Appropriateness, feasibility, or sustainability were evaluated in ten studies that reported challenges during implementation, including: inadequate transport; the need to cover long distances; inadequate compensation and supplies; and treatment-seeking behaviours associated with not undergoing diagnostic testing due to patients’ not wanting to spend money on tests, preference for home-based over laboratory-based testing, not wanting to travel to a laboratory, and lack of perception of the need for a test. Even when using rapid tests, busy labs could not transmit results rapidly enough for clinical decision-making in real-time. Difficulties in sourcing and maintaining a continuous and adequate supply of tests at the local level were found to affect feasibility and sustainability of implementing rapid tests. The introduction of rapid tests into drug shops in Uganda was found to be feasible within a complex intervention that included guidelines for treatment and referral of negative patients, community sensitization about the test, regulation, or subsidization of the price of the tests, and a system for training and supervision to guarantee conformity of drug vendors [42, 80, 81, 83, 84, 93, 94, 96, 98, 99]. The study design and implementation of the rapid test in drug shops in Uganda and lessons learned were detailed in two papers [113, 114]. For other uses, it was found that DNA can be successfully extracted and amplified from positive tests in the field [115]. There were positive features of rapid tests, such as their perceived ease of use, the ability to indicate the patient’s name or code, and the ability to deposit reagents in same well (Additional file 3: Table S2: Details of included studies).

### Tuberculosis (TB)

There were 47 studies on tuberculosis of which 22 evaluated the performance of Xpert® MTB/RIF and reported sensitivities ranging from 58.7 to 100% compared to culture in one or more spontaneous or induced sputum samples, and specificities near 100%. Sensitivity in non-respiratory samples (including cerebrospinal fluid, pleural, peritoneal, pericardial, gastric juice, bone, and lymph nodes) was also high (> 75%). Sensitivity decreased in HIV-infected subjects and smear negative samples, but was not affected by anaemia when evaluated in HIV-infected subjects [116–137]. Alere Determine TB-LAM Ag (Alere Inc., Waltham, MA, USA), a lateral flow assay that detects the mycobacterial cell wall antigen lipoarabinomannan (LAM) in urine, showed low sensitivity of 28.2% (95% *CI:* 19–39) against culture and 57.6% (95% *CI:* 46.4–68.3) against Xpert® MTB/RIF in patients with HIV-associated TB with high specificity (> 98%) [138].

Impacts of Xpert® MTB/RIF implementation in respiratory and non-respiratory samples and of TB-LAM were evaluated in eleven studies, which found a consistent increase in detection of TB cases, a decrease in time-to-treatment of patients with rifampicin-resistant TB, and a small decrease in TB-attributed mortality. Delay in patient diagnosis was not associated with Ct values of Xpert® MTB/RIF (Spearman R^2^ = 0.001. *P =* 0.612) [118, 122, 139–147].

Three studies assessed the cost of rapid tests as part of diagnosis and treatment algorithms. They found that the largest cost driver was consumables: cartridges in the case of Xpert® MTB/RIF, increasing the mean cost per TB case diagnosed threefold, but slightly less costly in MDR-TB case diagnosis. Other direct and indirect costs were related to transport, medical care, and time in health facility, which favoured the implementation of Xpert® MTB/RIF [148–150].

The adoption of Xpert® MTB/RIF was found to be highly acceptable to lab technicians and improved the notification indicators in Brazil, although there were concerns around ensuring the necessary funding to sustain the intervention [147, 151]. Other obstacles to implementation included lack of key documents—such as updated guidelines, concise and clear standard operating procedures (SOP), and training modules—as well as failures in identifying presumptive MDR-TB cases. It was reported that notification forms needed to be adapted to minimize errors when using Xpert® MTB/RIF, and that a regular supply of cartridges and spare parts was required. It was felt that it was important to maintain the capacity to perform smear microscopy to follow up patients and to process samples that were too scanty to run Xpert® MTB/RIF. In addition, feasibility was linked to quality control, qualified staff, work organization, computerized labs, and staff replacement programs [143, 152, 153].

The ICT Tuberculosis test (AMRAD Corporation, Melbourne, Australia), in an original (ICT1) and a manufacturer-modified version (ICT2), showed up to 83% sensitivity using whole blood. However, the combined sensitivity of microscopy and serology was 37.9% in HIV-coinfected patients [154, 155]. The TB STAT PAK® assay showed 63.4% sensitivity and 100% specificity in the Philippines [156, 157]. A Mexican rapid immunologic test (PRIM) was 79.2% (95% *CI:* 67.2–87.5) sensitive and 100% (95% *CI:* 93.6–100) specific [158]. On-site TB IgG/IgM rapid test yielded 88% sensitivity and 55% specificity compared to Quantiferon [159]. We found one exploratory study with Hexagon chromatographic immunoanalysis [160]. The measurement of exhaled nitric oxide showed sensitivity and specificity below 80% [161] and one field study of the amplified *Mycobacterium tuberculosis* direct (AMTD) test (Gen-Probe, San Diego, CA, USA) showed higher sensitivities which varied in smear positive and negative HIV-infected patients [162]. (Additional file 3: Table S2).

### Visceral leishmaniasis

Nine studies on visceral leishmaniasis evaluated the performance, cost, or acceptability and appropriateness of commercial or in-house rapid tests. The sensitivity of recombinant K39 protein (rK39) rapid tests, such as IT-LEISH® (Bio-Rad Laboratories, Hercules, CA, USA, and DiaMed, Cressier, Switzerland) and Kalazar Detect® (InBios International, Seattle, WA, USA) ranged from 72.4 to 87.59% and specificity, from 99.6 to 100% in blood or serum [163, 164]. HIV infection decreased sensitivity to 60% [163], but performance was not affected by malnutrition [165]. The reproducibility of immunochromatographic strip rK39 was very low (kappa 0.14) in one study in Brazil [166]. In urine samples, the rK39 rapid test and KAtex® (Kalon Biological, Guildford, UK), a latex agglutination test based on the detection of a low-molecular weight (5–20 kDa) heat-stable carbohydrate antigen, were evaluated. The sensitivity of the rK39 rapid test was 100% (95% *CI:* 94.95–100%) and specificity was 86.33% (95% *CI:* 79.23–91.36%) [167] and sensitivity of KAtex® was 77.77% and specificity was 98.24% [168]. In an endemic area in Brazil, the direct cost of IT-LEISH® was estimated to be USD 6.62 compared to USD 6.72 for the Kala-Azar Detect® rapid test [169]. The IT LEISH was found to be more appropriate to implement in the same endemic area due to its use of capillary blood, the positive reaction of 96% of patients to the finger prick blood collection, and its acceptability among healthcare professionals [170]. The sensitivity of an in-house latex agglutination test based on A2 antigen (A2LAT) or promastigote lysates proteins (proLAT) were 88.4% (both tests), and specificity was 93.5% for A2LAT and 100% for proLAT [171]. (Additional file 3: Table S2: Details of included studies).

### Leptospirosis

Five studies carried out on leptospirosis assessed the performance of commercial and in-house rapid tests. Dip-S-Tick (PanBio InDx, Inc., Baltimore, MD, USA) and LeptoTek Dri Dot (bioMérieux) showed sensitivities from 32.9 to 72.3% and 50% to 80% in acute phase, respectively, which increased to 80% and 84% in convalescent samples, respectively. Specificity was high for both tests (> 95%) [172]. LeptoTek Lateral Flow (Organon Teknika Ltd., Ireland) showed sensitivities of 65.4% in acute samples and 80.9% in samples at a later stage with 93.6% specificity [173]. LEPTO dipstick was highly sensitive (93.3%) but poorly specific (25%) in a study in Venezuela [174] while in Cuba, it was reported that LeptoTek Lateral Flow and LeptoTek Dip Stick were implemented for rapid disease confirmation with sensitivities and specificities > 90%, and LeptoTek Dri Dot for the investigation of suspected cases. Lepto Cuba (Cuban latex test) had results comparable to the latter [175]. A novel in-house method yielded sensitivities similar to IgM ELISA in Brazil with lower specificity [176]. (Additional file 3: Table S2: Details of included studies).

### Filariasis

Five studies on filariasis assessed performance or acceptability of rapid tests. They reported excellent agreement, with kappa value of 0.825 (95% *CI:* 0.739–0.912) between BinaxNOW® Filariasis card tests and Filariasis Test Strips (FTS) (both from Alere Scarborough, Inc., Scarborough, ME, USA). They also reported problems with the study personnel’s handling of FTS tests and the fact that 1.7% of tests turned positive at 30 min and 3.8% at 12 h, with potential for false positive results [177]. An in-house Brugia Rapid dipstick showed 87% (95% *CI:* 66.4–97.2) sensitivity and 100% specificity, and was regarded by field staff as easy to use and useful for increasing drug compliance, as subjects could visualize results on their own [178]. A commercial test, Brugia Rapid® (Reszon Diagnostics International, Subang Java, Selangor, Malaysia) was compared to microfilaremia in two endemic areas and one non-endemic area, showing 1%, 15%, and 5% positivity by rapid test against a single participant (out of 1543 tested) positive for microfilaremia, respectively. At one of the study sites, 4% of results were invalid [179]. The two theses from Brazil assessed the ICT card test, with overall sensitivities of 94.4% and 100% and specificities of 90.7% and 84.4% [180, 181]. (Additional file 3: Table S2: Details of included studies).

### Enteric fever

For enteric fever, there were three studies: two assessed the performance of Tubex® (IDL Biotech, Borlange, Sweden) in febrile subjects and one assessed Diazo reagent. Using a Tubex score of ≥4 as a cut-off for positivity, the sensitivity was 69% and specificity, 95% in China and 56% (95% *CI:* 47–66) and 88% (95% *CI:* 82–94), respectively, in India, [182, 183]. The study from Nigeria found that the Diazo reagent had 27.3% sensitivity when compared to the Widal test [184]. (Additional file 3: Table S2: Details of included studies).

### Schistosomiasis

Three studies on schistosomiasis evaluated circulating cathodic antigen (CCA) immunochromatographic tests (Rapid Medical Diagnostics, Pretoria, South Africa) and an in-house IgG rapid diagnostic test in urine samples. In Uganda, two versions of CCA, the second believed to be less sensitive than the first, were compared to two, four or six Kato-Katz smears, resulting in overall CCA1/CCA2 sensitivities of 91%/70%, 89%/63%, and 88%/59%, and CCA1/CCA2 specificities of 47%/86%, 50%/90%, and 52%/91%, respectively [185]. In Brazil, CCA against two Kato-Katz tests was 85.4% (95% *CI:* 72.2–93.9) sensitive and 78% (95% *CI:* 62.4–89.4%) specific [186]. The rapid test for IgG evaluated in Kenya had 97% (95% *CI:* 91–100) sensitivity and 78% (95% *CI:* 67–89%) specificity [187]. (Additional file 3: Table S2: Details of included studies).

### Dengue

Two studies on dengue assessed the performance of rapid tests and the knowledge of diagnostic procedures among primary care personnel, respectively. In Taiwan, China, the SD BIOLINE Dengue DUO® rapid immunochromatographic test kit (Standard Diagnostics, Inc., Gyeonggi-do, Republic of Korea), interpreted as positive when either NS1 or IgM were positive, yielded sensitivities above 90% from day 0 to day 5 and specificities between 14.29 and 74.6% in the same period in the context of a DENV2 epidemic [188]. In Saudi Arabia, primary care physicians’ self-reported knowledge of about dengue was found to be excellent among 43.4% of participants, but with poor use of specific dengue diagnostics, partly due to their unavailability [189]. (Additional file 3: Table S2: Details of included studies).

### Leprosy

Two studies reported on leprosy assessed the impact and performance of the ML Flow rapid test, and one reported the acceptability of a “representative lateral flow-based rapid test.” A greater decrease in multibacillary cases was observed in health services in Brazil that participated in the ML Flow study (from 73.1% in 2000 to 53.3% in 2004) compared to non-participating services (from 80.6 to 72.2% in the same period). Agreement of rapid test with final classification given by the health centre for treatment purposes was substantial (kappa 0.77) [190]. In the Philippines, 95.9% of patients, 93.2% of household contacts, and 81.4% of community contacts thought rapid tests would be beneficial, with 88.6% of patients willing to “definitely” submit to testing, followed by 69.4% of households and 72.2% of community contacts [191]. (Additional file 3: Table S2: Details of included studies).

### Chagas disease

One study assessed the Chagas Detect Plus rapid test (InBios, Seattle, WA, USA) against conventional serology, with sensitivities of 96.2% in whole blood and 99.3% in serum and specificities of 98.8% in whole blood and 96.9% in serum [192]. (Additional file 3: Table S2: Details of included studies).

### Human African trypanosomiasis

One conference abstract reported kappa 0.27 (95% *CI:* 0.24–0.3) between rapid test SD BIOLINE® HAT (Standard Diagnostics Inc., Gyeonggi-do, Republic of Korea) and Card Agglutination Test for Trypanosomiasis (CATT) during routine screening [193]. (Additional file 3: Table S2: Details of included studies).

### Cholera

There was one study on cholera that showed 91.7% sensitivity and 72.9% specificity of the Crystal VC kit (Span Diagnostics, Surat, India) against culture in diluted stool samples from hospitalized subjects [194]. (Additional file 3: Table S2: Details of included studies).

## Discussion

We sought to identify which rapid diagnostic tests for VBDs and other infectious diseases of poverty in urban areas have been evaluated in the field and what were the evaluation results. The present study showed that rapid diagnostic tests are being used in urban areas, but that reporting on the field evaluation of these type of tests is dominated, by a large margin, by studies on malaria and tuberculosis. This could be attributed partially to the burden of disease, the availability of devices to be tested, and the research priorities of funding agencies. Fewer studies were found on neglected tropical diseases (visceral leishmaniasis, filariasis, leptospirosis, enteric fever, schistosomiasis, dengue, leprosy, Chagas disease, human African trypanosomiasis, and cholera), supporting the need to further improve research and product development, including rapid diagnostics, in these groups of diseases. The lack of studies on the other diseases included in our search strategy (viral encephalitis, viral haemorrhagic fevers, echinococcosis, rickettsial diseases, onchocerciasis, and trachoma) might reflect the even lower investment in diagnostic development for these diseases, the lack of a perceived need for rapid diagnostic tests in the urban context, or limitations of our search strategy. The search identified both commercial and in-house tests, showing the potential for product development outside the industry that could be further explored, particularly for neglected tropical diseases.

Performance is, logically, a key characteristic of diagnostic tests to be prioritized for field evaluation and, hence, at least one field performance evaluation per disease was identified. In malaria, a relatively large number of rapid tests are available on the market; hence, there is a greater need for regulation and quality assurance in this area. We identified studies that compared the performance of several commercial tests simultaneously. Results from this type of study would be useful to providers faced with having to choose from among several rapid diagnostics options. The advantages and disadvantages of such studies have been highlighted [195]. Performance studies were carried out in several countries on several continents, but local studies of malaria rapid diagnostics may be required, since the results from one region cannot necessarily be extrapolated to another, as shown by a systematic review [196]. Continent was identified as a source of heterogeneity in test performance, with lower sensitivity and specificity in Africa than in Asia, and high specificity and low sensitivity in South America. Heterogeneity within the same continent was not assessed, and this systematic review included only *P. falciparum* malaria [196]. A separate systematic review analyzed the performance of rapid tests for non-falciparum malaria [197]. The separate analysis by falciparum and non-falciparum malaria does not necessarily reflect the performance of the diagnostics under field conditions where several species co-exist. Therefore, the field performance of tests could be further affected by the prevalence of *Plasmodia* over time and other factors not analyzed, such as whether the rapid tests are used in the hospital or community context. Despite the few studies identified in our review, there was a systematic review of rapid diagnosis of malaria in pregnancy that included a relatively large number of studies from Africa, but not enough to compare performance between rapid tests or parasite species [198].

For tuberculosis, most studies evaluated the performance of Xpert® MTB/RIF with heterogeneous results, but the sources of heterogeneity were clearer compared to malaria rapid tests. Decreased sensitivity in respiratory samples was reported in smear negative cases and HIV-infected cases, as confirmed by a systematic review [199]. Other sources of heterogeneity identified by meta-regression were number of samples, incidence of TB in the samples, quality of reporting, and proportion of smear-positive samples [200]. For non-respiratory samples, reported sensitivities of > 75% were similar to the findings of a systematic review [200]. Likewise, the low sensitivity of TB-LAM found was confirmed in a systematic review [201]. Detection of tuberculosis human antibodies by immunochromatography, such as ICT, On-site TBIgG/IgM, TB STAT PAK®, and PRIM, yielded variable sensitivity and specificity; as such, this would appear to be an interesting potential topic for a systematic review.

Our search retrieved relatively few studies on visceral leishmaniasis, dengue, schistosomiasis, leprosy, enteric fever, Chagas disease, and human African trypanosomiasis, even though at least one systematic review of diagnostics for each of these diseases has been previously published. In visceral leishmaniasis, most studies were conducted with rK39 as the target, although other rapid tests with other targets have been developed. A recent systematic review of only rK39-based tests suggests that rapid tests or ELISA are good choices for implementation [202]. The fact that our search was restricted to field evaluations might explain the relatively small number of studies on this disease. It is to be expected that more field evaluations and implementation studies will be performed in the future to confirm some preliminary findings pointing towards not differences in performance between various rK39 available tests and the presence of heterogeneity between East Africa and the Indian subcontinent [203]. A similar explanation of search being restricted to field evaluation could be provided for the other diseases. In dengue, IgM and NS1-based tests have been addressed in systematic reviews separately, with variable sensitivities and identified sources of heterogeneity; however, results suggest that simultaneous detection of both markers would improve their performance, and hence more studies in the future to assess the combination of tests would be useful [204, 205]. In schistosomiasis, we observed the effect of the gold standard used. A systematic review summarized the challenges of assessing the performance of schistosomiasis diagnostics that could be more sensitive than the commonly used reference test (Kato-Katz smear) [206]. In leprosy, the ML Flow rapid test that detects IgM to specific phenolic glycolipid-I (PGL-I) is used to improve the classification of multibacillary cases and hence to assist in clinical diagnosis, but not as a stand-alone diagnostic test [207]. The performance of rapid tests for enteric fever does not support their routine use, at least not by themselves, but potentially in combination with other tests [208]. While the rapid test for Chagas disease was highly sensitive and specific, the performance of the rapid test for human African trypanosomiasis was disappointing. For Chagas disease and human African trypanosomiasis, the available systematic reviews do not include the identified rapid tests we found in this search [209, 210]. A systematic review of rapid tests for Chagas disease could provide the evidence required to inform decisions. This is also true for filariasis and leptospirosis, which showed contrasting results in the performance of the available rapid tests.

The impacts and implementation outcomes of rapid tests—mainly acceptability and cost, followed by adoption, feasibility, and sustainability—are being evaluated. These studies shed light on both the facilitators and the challenges encountered when using rapid diagnostics under real-life conditions, ranging from cultural to technical and administrative issues. The reported reduction in antimalarial drugs use as a result of the implementation of rapid tests is consistent with a systematic review of controlled trials in rural endemic areas that also found the impact of malaria rapid tests to be directly correlated with provider compliance [211], which in turn depends on other factors [212]. Interestingly, the impact of Xpert® MTB/RIF in improving TB case detection does not necessarily imply the withdrawal of other diagnostic methods (e.g. smear microscopy), but rather their more selective use. The implementation outcomes results observed in the present study help to inform on what other contextual factors (cultural, political, socioeconomic, health system, institutional) should be taken into account. Some of these are: community beliefs related to the sample or specimen (blood, sputum, urine, etc.); trust in the accuracy of the test; ability to discern the target of the test (e.g. patients believing they are being tested for HIV and not for malaria); burden of work to providers; sustainability of supplies; training issues; and routine quality assessment. Table 3 summarizes the identified knowledge gaps and priority needs for future research.

**Table 3:** Knowledge gaps and priority needs for future research

• Increase research and development of rapid diagnostics for neglected tropical diseases. • Assess the performance of several diagnostic tests simultaneously. • Develop a framework to assess sources of heterogeneity in the performance of rapid diagnostics. • Conduct systematic reviews of diagnostic performance of rapid tests for TB, Chagas disease, filariasis, leptospirosis, and simultaneous detection of NS1 and IgM for dengue. • Harmonize protocols for validation of schistosomiasis, leptospirosis, and enteric fever rapid diagnostics	

### Strengths and limitations of this review

To widen the coverage of potential relevant studies, the search strategy included documents in four languages (English, Spanish, French, and Portuguese) and a comprehensive list of infectious diseases. In fact, a substantial number of titles and abstracts were retrieved. The aim of this scoping review was to describe the tests and results of field evaluations of rapid diagnostics and not to be exhaustive. We believe the use of a key concept representing “urban area” resulted in decreased sensitivity, since information on whether the study was conducted in an urban area is not systematically reported or compulsorily requested for publication. For this same reason, we cannot rule out the misclassification of included studies as urban. Hence, we suggest conducting future scoping reviews without a term for urban area, to increase sensitivity, but restricting them to a smaller list of diseases to keep the reviews feasible. The extraction process revealed there was not enough information on who performed the rapid tests. This points to the need to improve the reporting of diagnostic studies. QUADAS 2 was not used to analyze the quality of the studies, as we used a standardized extraction grid for this series of scoping reviews [213]. We suggest considering the inclusion of implementation outcomes in the standardized reporting guidelines of diagnostic studies.

### Implications for public health policy and/or practice

Rapid diagnostic tests for VBDs and other diseases of poverty are being used in the urban context with demonstrated positive impacts on case detection, rational use of drugs, and even decreased mortality. Nevertheless, most evidence comes from malaria rapid tests, where there are multiple options whose performance is heterogeneous. Decision-makers are advised to consider the potential sources of heterogeneity in the performance of diagnostic tests when deciding whether to implement them in their own context; these include geographical region, patients’ characteristics (e.g. HIV status, pregnancy), and providers’ level of expertise. Nonetheless, in some situations, it is neither easy nor feasible to wait for the evidence expected from required multicountry studies; in such cases, the recommended strategy would be to analyze the availability, costs, and prior results of rapid diagnostics within the country or in comparable geographical regions to inform decisions. When faced with multiple options for rapid diagnostics, or with lack of data, comparative operational research might be needed to verify whether those rapid diagnostics work under routine conditions and are acceptable to providers and patients, as well as to confirm their implementation feasibility in representative contexts before rolling them out.

This review highlights that, for practitioners and public health workers, besides issues of performance and sources of heterogeneity, when implementing rapid diagnostics it is essential to take into account the beliefs of communities and providers, their trust in the accuracy of the tests, the burden of work to providers, the sustainability of supply chains, training issues, and quality assurance, among other factors. Hence, pragmatic research is recommended to assess acceptability, feasibility, cost, and sustainability in representative contexts. Monitoring impacts, cost-effectiveness, correct use, quality, and long term sustainability is necessary to fully justify the decision to implement and maintain the investment in rapid diagnostics. Table 4 summarizes the implications for public health policy and practice.

**Table 4:** Implications for public health policy and practice

• Take context into account when deciding on the use of rapid diagnostics, as performance, impact, and implementation outcomes are highly variable. • Consider the beliefs of communities and providers before implementing rapid diagnostics. • Confirm that rapid diagnostics are feasible to implement in representative contexts before rolling out. • Burden of work to providers, sustainability of supplies, training, and routine quality assessment are key factors to be considered before implementation. • Invest in pragmatic evaluation before, during, and after implementation.	

## Conclusions

The present scoping review provides an overview of the field validation and implementation studies of rapid diagnostic tests for VBD and other infectious diseases of poverty in urban areas. We identified a relatively large number of available malaria rapid diagnostic tests that have been evaluated and fewer options for other diseases, such as visceral leishmaniasis, dengue, Chagas disease, leptospirosis, filariasis, and leprosy. The field performance of malaria rapid diagnostics depends upon epidemiological, technical and clinical factors. There is evidence of their positive impact in decreased use of presumptive treatment but challenges remain to achieve their successful implementation. Based on their reported good performance, Xpert® MTB/RIF for tuberculosis and rapid diagnostics for visceral leishmaniasis that detect rK39 warrant more implementation studies. Multicentre studies and/or systematic reviews of rapid diagnostics for dengue using simultaneous detection of both NS1 and IgM, as well as for Chagas disease, filariasis, and leptospirosis, are required to provide evidence on their performance and hence, suitability of further implementation studies.

There is a need to establish valid reference standards to evaluate the performance of rapid diagnostic tests, particularly for schistosomiasis, and harmonised field validation research protocols to take into account potential sources of heterogeneity such as geographical region, assay, HIV co-infection, infection type and load, and epidemiological characteristics. This is likely to demand a global (multicountry or multicontinent) approach to field validation of rapid diagnostic tests. Implementation studies that assess rapid diagnostics as part of complex interventions rather than by themselves are likely to be required to identify solutions to some of the challenges encountered in their implementation in real-life situations.

## Additional files

Additional file 1: Multilingual abstracts in the six official working languages of the United Nations. (PDF 484 kb)

Additional file 2: **Table S1.** Search strategy. (DOC 222 kb)

Additional file 3: **Table S2.** Details of included studies. (DOCX 234 kb)

## Abbreviations

ASTAIRE: Analysis of the transferability of health promotion interventions
ELISA: Enzyme-linked immunosorbent assay
MMAT: Mixed methods appraisal tool
TDR: The special programme for research and training in tropical diseases
TIDieR: Template for intervention description and replication
VBDs: Vector-borne diseases
WHO: World Health Organization
