# Supplementary material for: A scoping review on the field validation and implementation of rapid diagnostic tests for vector-borne and other infectious diseases of poverty in urban areas
Source: Infect Dis Poverty. 2018 Sep 3;7:87. doi: 10.1186/s40249-018-0474-8 (PMC6120097; doi:10.1186/s40249-018-0474-8)
Supplement: Supplementary file 1 — Multilingual abstracts in the six official working languages of the United Nations. (PDF 484 kb) [file 40249_2018_474_MOESM1_ESM.pdf]

Translation of the abstract into the six official working languages of the United Nations

دراسة استطلاعية للتحقق الميداني من صحة وتنفيذ الاختبارات التشخيصية السريعة للأمراض المنقولة وغيرها من أمراض الفقر المعدية في المناطق الحضرية

ليدا اوسوريو، وجوني اليخاندرو غارسيا، ولويس غابرييل بارا، وفيكتور غارسيا، ولورا توريس، وستيفاني ديرودتي، وفاليري ريدي

ملخص

معلومات أساسية: يواجه الموظفون الصحيون تحديات في تشخيص أمراض الفقر المنقولة وغيرها في البيئات الحضرية. وهناك حاجة إلى معرفة التكنولوجيات التشخيصية السريعة المتاحة، وتقييمها بشكل سليم، ويجري تنفيذها لتحسين السيطرة على هذه الأمراض في السياق الحضري. وتناقش هذه الدراسة الأدلة المتعلقة بالتحقق الميداني والتنفيذ في المناطق الحضرية للتشخيص السريع للأمراض المنقولة بالنواقل وغيرها من أمراض الفقر.

الجزء الرئيسي: أُجريت مراجعة استطلاعية. وجرى البحث في المنشورات غير الرسمية واستعراض النظائر باستخدام مصطلحات تصف الأمراض المعدية المستهدفة، وتقييمات التشخيصات، والاختبارات السريعة، والبيئة الحضرية. واقتصرت المراجعة على الدراسات التي نشرت بين عامي 2000 و 2016 باللغات الانجليزية والاسبانية والفرنسية والبرتغالية. وتم صقل معايير الاشتمال والاستبعاد لتحديد المنشورات ذات الصلة بغض النظر عن تصميم الدراسة والجغرافيا. وأُدرج في التحليل ما مجموعه 179 وثيقة من 7806 وثيقة التي فحصت في البداية. وشكل مرض الملاريا (*n* = 100) والسل (*n* = 47) بغالبية الدراسات التي أبلغت عن الأداء التشخيصي والأثر ونتائج التنفيذ. وتم تحديد عدد اقل من الدراسات، التي تقيم أساسا الأداء، بالنسبة لداء الليشمانيات الحشويه (*n* = 9)، وداء الخيطيات وداء البلهارسيا (*n* each = 5) ، والحمى المعوية، والهارتورات (*n* each = 3)، وحمى الدنج والملاريا (*n* each = 3)، ومرض شاغاس، وداء المثقبيات الافريقي البشري، والكوليرا (*n* each = 1). وكانت حساسية التجارب السريعة المبلغ عنها متباينة تبعا لعدة عوامل. وإجمالا، كانت الخصوصيات مرتفعة (أكثر من 80 في المائة)، باستثناء البلهارسيا والكوليرا. ويجري في الميدان تقييم نتائج الأثر والتنفيذ، و بصفة رئيسية درجة القبول والتكلفة، يليها الاعتماد، والجدوى، واستدامة الاختبارات السريعة. وتتراوح التحديات التي تواجه تنفيذ الاختبارات السريعة بين القضايا الثقافية والتقنية والإدارية.

الاستنتاجات: يجري استخدام الاختبارات التشخيصية السريعة لأمراض الفقر المنقولة وغيرها في السياق الحضري مع التأثير الواضح على الكشف عن الحالات. ومع ذلك، فإن معظم الأدلة تأتي من التشخيص السريع للملاريا، مع نتائج متفاوتة. وفي حين أن الاختبارات السريعة للسل وداء الليشمانيات العميق تتطلب مزيدا من الدراسات التنفيذية، فإن هناك حاجة إلى المزيد من الأدلة على أداء الاختبارات الحالية أو استحداث بدائل جديدة لحمى الدنك، ومرض شاغاس، وداء الخيطيات، والليبتوبيوروسيس، والحمى المعوية وداء المثقبيات الإفريقي البشري والبلهارسيا والكوليرا.

Translated from English version into Arabic by Free bird, proofread by Abdessalam AIT TOUIJAR, through

TRANSLATORS
WITHOUT BORDERS

对城市地区媒传疾病和其他贫困所致传染病快速诊断试验（RDT）的现场验证和实施：堪域综述

Lyda Osorio, Jonny Alejandro Garcia, Luis Gabriel Parra, Victor Garcia, Laura Torres, Stéphanie Degroote, Valéry Ridde

摘要

引言：卫生人员在诊断城市地区的媒传疾病和其他贫困所致传染病方面仍面临许多挑战。因此，需要了解目前哪些快速诊断技术是可用的，哪些已得到了适当的评估，并正在城市环境实施以促进对这些疾病的控制。本文综述了在城市地区对媒传疾病和其他贫困所致传染病进行快速诊断的现场验证和实施的证据。

主要内容：通过使用描述目标传染病、诊断评估、快速检测和城市环境的相关术语对经同行评议的文献和灰色文献进行检索，检索时限为 2000–2016 年，文种包括英文、西班牙文、法文和葡萄牙文。设定纳入标准和排除标准以确定相关文献，不考虑研究设计和地理范围。
本研究最终纳入了 179 篇文献（来自初筛的 7806 篇）。疟疾(*n* = 100)和结核病(*n* = 47)的相关研究最多，主要涉及诊断性能、影响和实施结果的研究。少数针对以下疾病的 RDT 主要性能的评估，包括内脏利什曼病(*n* = 9)，丝虫病和钩端螺旋体病(每种疾病 *n* = 5)，伤寒肠热病和血吸虫病(每种疾病 *n* = 3)，登革热和麻风(每种疾病 *n* = 3)，及恰加斯病、非洲人类锥虫病和霍乱(每种疾病 *n* = 1)。据报道 RDT 灵敏度受到多种因素的影响。总体而言，除血吸虫病和霍乱外，特异性较高(> 80%)。快速诊断试剂的影响和实施结果主要有可接受性和成本，以及适用性、可行性和在现场环境中评估的可持续性。实施快速检测的挑战包括文化、技术和行政问题。
结论：在城市环境中，快速诊断试验正被应用于媒传疾病和其他贫困所致传染病的诊断，并已证明对病例检测有显著影响。然而，大多数证据来自疟疾快速诊断，且结果各不相同。虽然结核病和内脏利什曼病的快速检测需要作进一步的实施研究，但对于登革热、恰加斯病、丝虫病、钩端螺旋体病、肠热病、人类非洲锥虫病、血吸虫病和霍乱，需要更多的证据证明目前检测的有效性或开发新的替代品。

Translated from English version into Chinese by Xin-Yu Feng, edited by Pin Yang

CHINESE CENTER FOR DISEASE CONTROL AND PREVENTION
NATIONAL INSTITUTE OF PARASITIC DISEASES

Étude de la portée de la validation sur le terrain et de la mise en œuvre de tests de diagnostic rapide pour les maladies à transmission vectorielle et autres maladies infectieuses dues à la pauvreté dans les zones urbaines

Lyda Osorio, Jonny Alejandro Garcia, Luis Gabriel Parra, Victor Garcia, Laura Torres, Stéphanie Degroote, Valéry Ridde

Résumé

Contexte: Le personnel médical est confronté à des difficultés concernant le diagnostic des maladies à transmission vectorielle et autres maladies dues à la pauvreté en milieu urbain. Il est nécessaire de savoir quelles technologies de diagnostic rapide sont disponibles, ont été convenablement évaluées, et sont mises en œuvre pour améliorer le contrôle de ces maladies dans le contexte urbain. Cet article définit les preuves de la validation sur le terrain et de la mise en œuvre, dans les zones urbaines, de diagnostics rapides pour les maladies à transmission vectorielle et autres maladies dues à la pauvreté.
Développement: Une étude de la portée a été menée. La littérature scientifique et la littérature grise ont été examinées en recherchant les termes décrivant les maladies infectieuses ciblées, les évaluations de diagnostics, les tests rapides et le milieu urbain. L'étude était limitée aux recherches publiées entre 2000 et 2016 en anglais, en espagnol, en français et en portugais. Les critères d'inclusion et d'exclusion ont été affinés a posteriori afin d'identifier la littérature pertinente, indépendamment de la conception de l'étude, et de la géographie.
Sur 7806 initialement sélectionnés, 179 documents au total ont été inclus dans l'analyse. Le paludisme (*n* = 100) et la tuberculose (*n* = 47) représentaient la majorité des recherches ayant rapporté la performance et l'impact des diagnostics, et les résultats de mise en œuvre. Un plus petit nombre de recherches, évaluant principalement la performance, ont été identifiées pour la leishmaniose viscérale (*n* = 9), la filariose et la leptospirose (chacune*n* = 5), la fièvre typhoïde et la schistosomiase (chacune*n* = 3), la dengue et la lèpre (chacune*n* = 3), et la maladie de Chagas, la trypanosomiase humaine africaine, et le choléra (chacuns*n* = 1). La sensibilité rapportée des tests rapides était variable en fonction de plusieurs facteurs. Dans l'ensemble, les spécificités étaient hautes (>80%), hormis pour la schistosomiase et le choléra. Les résultats

d'impact et de mise en œuvre, principalement l'admissibilité et le coût, mais aussi l'adoption, la faisabilité, et la durabilité des tests rapides, sont en cours d'évaluation sur le terrain. Les difficultés de la mise en œuvre des tests rapides vont des défis culturels aux problèmes techniques et administratifs.
Conclusions: Des tests de diagnostic rapide pour les maladies à transmission vectorielle et autres maladies dues à la pauvreté sont utilisés dans le contexte urbain avec un impact prouvé sur la détection des cas. Cependant, la plupart des preuves viennent des diagnostics rapides de paludisme, avec des résultats variables. Tandis que les tests rapides pour la tuberculose et la leishmaniose viscérale nécessitent des études de mise en œuvre plus approfondies, davantage de preuves sur la performance des tests actuels ou le développement de nouvelles alternatives sont nécessaires pour la dengue, la maladie de Chagas, la filariose, la leptospirose, la fièvre typhoïde, la trypanosomiase humaine africaine, la schistosomiase et le choléra.

Translated from English version into French by Mathilde Grignon, proofread by Sophie N, through

Предварительный обзор полевых испытаний и внедрения диагностических экспресс-анализов на трансмиссивные и другие инфекционные болезни бедных слоев населения в городских районах

Лида Осорио, Джонни Алехандро Гарсия, Луис Габриэль Парра, Виктор Гарсия, Лаура Торрес, Стефани Дегрот, Валери Ридд

Аннотация

Краткое описание: Работники здравоохранения сталкиваются с трудностями при диагностировании трансмиссивных и других болезней бедных слоев населения в условиях города. Необходимо отметить, что диагностические экспресс-технологии существуют; они были надлежащим образом оценены и в настоящее время проходят внедрение для улучшения борьбы с этими болезнями в городской среде. В данной статье приводятся фактические данные полевых испытаний и внедрения диагностических экспресс-анализов на трансмиссивные и другие инфекционные болезней бедных слоев населения в городских районах
Основная часть: Был выполнен предварительный обзор. В рецензируемой и внеиздательской литературе был проведен поиск по терминам, описывающим искомые инфекционные болезни, диагностические оценки, экспресс-анализы и городскую среду. Обзор ограничивался исследованиями, опубликованными между 2000 и 2016 годами на английском, испанском, французском и португальском языках. Критерии включения и исключения после получения результатов были дополнительно уточнены для отбора литературы, имеющей отношение к рассматриваемой теме независимо от планов исследований и их географии.
Всего в анализ было включено 179 из 7806 первоначально отобранных документов. Большинство исследований, в которых сообщались данные об эффективности диагностики, эффекте и результатах внедрения, было посвящено малярии (n = 100) и туберкулезу (n = 47). Меньшее число исследований, которые, главным образом, давали оценку эффективности, было связано с висцеральным лейшманиозом (n = 9), филяриозом и лептоспирозом (каждая болезнь n = 5), кишечной лихорадкой и шистосомозом (каждая болезнь n = 3), лихорадкой денге и проказой (каждая болезнь n = 3), а также с болезнью Шагаса, африканским трипаносомозом человека и холерой (каждая

болезнь n = 1). Данные о чувствительности к экспресс-анализам различались в зависимости от нескольких факторов. Общие специфичности были высокими (>80%), за исключением шистосомоза и холеры. В полевых условиях оценивались эффект и результаты внедрения экспресс-анализов; в первую очередь, их приемлемость и стоимость с последующей оценкой принятия к использованию, практической осуществимости и пригодности к длительному применению. Проблемы с внедрением экспресс-анализов охватывали широкий диапазон от культурных и технических до административных трудностей.
Заключение: Применение в настоящее время в городских условиях диагностических экспресс-анализов на трансмиссивные и другие болезни бедных слоев населения оказывает подтвержденный эффект на выявление случаев заболевания. Однако, большая часть фактических данных поступает от экспресс-диагностики на малярию, при этом результаты варьируются. Экспресс-анализы на туберкулез и висцеральный лейшманиоз требуют дальнейших исследований их внедрения, а выполняемые в настоящее время анализы на лихорадку денге, болезнь Шагаса, филяриоз, лептоспироз, кишечную лихорадку, африканский трипаносомоз человека, шистосомоз и холеру нуждаются в дополнительных свидетельствах их эффективности, или необходима разработка новых альтернативных методов.

Translated from English version into Russian by Natalia Potashnik, proofread by Tatiana Kary, through

TRANSLATORS WITHOUT BORDERS

Revisión del alcance de la comprobación en el terreno y la puesta en práctica de métodos de diagnóstico rápido para enfermedades transmitidas por vectores y otras enfermedades infecciosas asociadas con la pobreza en zonas urbanas.

Lyda Osorio, Jonny Alejandro Garcia, Luis Gabriel Parra, Victor Garcia, Laura Torres, Stéphanie Degroote, Valéry Ridde

Resumen

Antecedentes: El personal sanitario encuentra dificultades para diagnosticar enfermedades transmitidas por vectores y otras enfermedades asociadas con la pobreza en entornos urbanos. Es necesario conocer qué técnicas de diagnóstico rápido están disponibles, que han sido evaluadas apropiadamente y se están ejecutando para mejorar el control de estas enfermedades en el ambiente urbano. En este trabajo se describen los resultados de la validación y la puesta en práctica en el terreno, en zonas urbanas, de métodos de diagnóstico rápido para enfermedades transmitidas por vectores y otras enfermedades infecciosas asociadas con la pobreza.
Cuerpo principal: Se realizó una revisión sistemática. Se analizaron artículos científicos y de "literatura gris" en la búsqueda de términos que describieran estas enfermedades específicas, evaluaciones diagnósticas, pruebas rápidas y ambiente urbano. La revisión se limitó a estudios publicados entre los años 2000 y 2016 en inglés, español, francés y portugués. Los criterios de inclusión y de exclusión se acotaron a posteriori a fin de identificar la literatura relevante con independencia del diseño del estudio y la geografía.
En el análisis se incluyeron 179 de los 7806 documentos que habían sido examinados al principio. La mayor parte de los estudios que informaban el rendimiento diagnóstico, el impacto y los resultados de la puesta en práctica se referían a la malaria (n = 100) y a la tuberculosis (n = 47). Una menor cantidad de estudios, referidos en su mayor parte al rendimiento,

identificaban la leishmaniasis visceral (*n* = 9), la filariasis y la leptospirosis (*n* = 5 cada una), la fiebre entérica y la esquistosomiasis (*n* = 3 cada una), el dengue y la leptospirosis (*n* = 3 cada una), y la enfermedad de Chagas, la tripanosomiasis humana africana y el cólera (*n* = 1 cada una). La sensibilidad a las pruebas rápidas informada fue variable y dependiendo de diversos factores. En general, las especificidades fueron elevadas (> 80%), excepto para la esquistosomiasis y el cólera. El impacto y los resultados de la implementación, principalmente la aceptabilidad y el costo, seguidos por la adopción, la factibilidad y la sustentabilidad de las pruebas rápidas se están evaluando en el terreno. Los desafíos de implementar las pruebas rápidas van desde lo cultural hasta los aspectos técnicos y administrativos.

Conclusiones: Las pruebas de diagnóstico rápido para enfermedades transmitidas por vectores y otras enfermedades asociadas con la pobreza se están utilizando en el ambiente urbano con efecto comprobado sobre la detección de casos. Sin embargo, la mayor parte de la evidencia proviene de pruebas de diagnóstico rápido para malaria, con resultados variables. Mientras que las pruebas rápidas para tuberculosis y leishmaniasis visceral requieren más estudios para su implementación, se necesitan más pruebas de los resultados de las pruebas actuales o el desarrollo de nuevas alternativas para dengue, enfermedad de Chagas, filariasis, leptospirosis, fiebre entérica, tripanosomiasis humana africana, esquistosomiasis y cólera.

Translated from English version into Spanish by Lidia Norese, proofread byPatricia Cassoni, through

TRANSLATORS
WITHOUT BORDERS
