# Supplementary material for: A scoping review on the field validation and implementation of rapid diagnostic tests for vector-borne and other infectious diseases of poverty in urban areas
Source: Infect Dis Poverty. 2018 Sep 3;7:87. doi: 10.1186/s40249-018-0474-8 (PMC6120097; doi:10.1186/s40249-018-0474-8)
Supplement: Supplementary file 2 — Table S1. Search strategy. (DOC 222 kb) [file 40249_2018_474_MOESM2_ESM.doc]

**Table S1. Search Strategy**

1.1. Search for MEDLINE (Pubmed):

| “African tick bite fever”[tiab] OR “African trypanosomiasis”[tiab] OR Alphaviruse*[tiab] OR “American trypanosomiasis”[tiab] OR Arbovirus*[tiab] OR  “arthropod borne disease*"[tiab] OR “Arthropod-borne virus*”[tiab] OR “bacterial disease*”[tiab] OR Bartonellosis[tiab] OR Bilharzias*[tiab] OR “Borrelia infections”[MeSH Terms] OR borreliosis[tiab] OR Borrelia*[tiab] OR “break-bone fever”[tiab] OR “Boutonneuse fever”[MeSH Terms] OR “Buruli ulcer”[MeSH Terms] OR “buruli ulcer”[tiab] OR “Chagas disease”[MeSH Terms] OR “chagas disease”[tiab] OR “Chikungunya virus”[MeSH Terms] OR Chikungunya[MeSH Terms] OR chikungunya[tiab] OR Cholera[MeSH Terms] OR cholera[tiab] OR “Colorado tick fever”[tiab] OR “Communicable diseases”[MeSH Terms] OR “communicable disease*”[tiab] OR “Communicable Diseases, Emerging”[MeSH Terms] OR [Cysticercosis](https://www.ncbi.nlm.nih.gov/mesh/68003551)[MeSH Terms] OR cysticercosis[tiab] OR “Crimean-congo haemorrhagic fever*”[tiab] OR Dengue[MeSH Terms] OR dengue[tiab] OR “[Hemorrhagic Fever, Ebola](https://www.ncbi.nlm.nih.gov/mesh/68019142)”[MeSH Terms] OR Ebolavirus[MeSH Terms] OR “ebola virus”[tiab] OR [Echinococcosis](https://www.ncbi.nlm.nih.gov/mesh/68004443)[MeSH Terms] OR “encephalitis, viral”[MeSH Terms] OR “encephalitis virus*”[tiab] OR “enteric fever”[tiab] OR “Ehrlichia chaffeensis”[tiab] OR “Ehrlichia ewingii”[tiab] OR “erythema chronicum migrans”[tiab] OR “erythema migrans”[tiab] OR filariasis[MeSH Terms] OR Elephantiasis[MeSH Terms] OR “Elephantiasis, filarial”[MeSH Terms] OR “filovirus diseases”[tiab] OR “Flea borne spotted fever”[tiab] OR “Food-borne trematodiases"[tiab] OR “Francisella tularensis”[tiab] OR “Hansen disease”[tiab] OR “Hantavirus fever”[tiab] OR “Hemorrhagic Fever Virus, Crimean-Congo”[MeSH Terms] OR “Hemorrhagic fever, omsk”[MeSH Terms] OR “Hendra virus”[MeSH Terms] OR “Katayama fever”[tiab] OR “Lassa fever”[tiab] OR Leishman*[tiab] OR “Leprosy”[MeSH Terms] OR “Leptospirosis”[MeSH Terms] OR Leptospir*[tiab] OR Lyme*[tiab] OR Malaria[MeSH Terms] OR Marburg*[tiab] OR “Neglected diseases”[MeSH Terms] OR nipah[tiab] OR Neuroschistosomias*[tiab] OR “North asian tick typhus”[tiab] OR Onchocerciasis[MeSH Terms] OR Onchocerc*[tiab] OR “Oriental spotted fever”[tiab] OR “Orientia tsutsugamushi”[tiab] OR “Pappataci fever”[tiab] OR “parasitic disease*”[tiab] OR “Paratyphoid fever”[MeSH Terms] OR “Phlebotomus fever”[MeSH Terms] OR “Phlebovirus”[MeSH Terms] OR “Plague”[MeSH Terms] OR “Plague*”[tiab] OR Plasmodium[tiab] OR “Coxiella burnetii”[MeSH Terms] OR “Q fever”[tiab] OR Rabies[MeSH Terms] OR Rabies[tiab] OR “Re emerging infectious disease*”[tiab] OR “Relapsing fever”[tiab] OR “Remittent fever”[tiab] OR Rickettsi*[tiab] OR “Rickettsia infections”[MeSH Terms] OR “Rift valley fever”[MeSH Terms] OR “River Blindness”[tiab] OR “Robles Disease*”[tiab] OR “Rocky mountain spotted fever”[tiab] OR “Salmonella typhi”[tiab] OR “Salmonella enterica”[tiab] OR “Sandfly fever”[tiab] OR “Schistosomiasis”[MeSH Terms] OR “Schistosomia*”[tiab] OR “Schistosoma*”[tiab] OR “Sicilian virus”[tiab] OR “sleeping sickness”[tiab] OR “Spotted fever”[tiab] OR “three-day fever”[tiab] OR “Toscana virus”[tiab] OR Trachoma[Mesh Terms] OR Trachoma[tiab] OR “tick-borne infection”[tiab] OR “tick-borne viral disease*”[tiab] OR “tick-borne bacterial disease*”[tiab] OR “tick borne parasite”[tiab] OR “Tick borne viral disease*”[tiab] OR “Tropical disease*”[tiab] OR “Tropical neglected disease”[tiab] OR “Trypanosom*”[tiab] OR “Tuberculosis”[Mesh Terms] OR “Tularemia”[Mesh Terms] OR “Typhoid fever”[MeSH Terms] OR “Typhoid”[tiab] OR “Typhus*”[tiab] OR “Vector borne infection”[tiab] OR “Vector-borne pathogen*”[tiab] OR “Viral encephalitis”[tiab] OR "Virus hemorrhagic fever"[tiab] OR “water borne disease”[tiab] OR “Weil disease”[MeSH Terms] OR “West Nile fever”[Mesh Terms] OR “West Nile Virus”[tiab] OR “West Nile neuroinvasive”[tiab] OR “West Nile Meningitis”[tiab] OR “West Nile meningoencephalitis”[tiab] OR “West Nile poliomyelitis”[tiab] OR “Yellow fever”[MeSH Terms] OR “Yersinia pestis”[MeSH Terms] OR “Zika virus infection”[MeSH Terms] OR Zika*[tiab] OR Zoonos*[tiab] OR “zoonotic pathogen*”[tiab] **AND** | Target VBD and other infections |
| --- | --- |
| “Diagnostic techniques and Procedures”[MeSH Terms] OR “Urine sample”[tiab] OR “Blood sample”[tiab] OR “Saliva sample”[tiab] OR “feces sample”[tiab] OR “sputum sample”[tiab] OR swab[tiab] OR fingerprick[tiab] OR biopsy[tiab] OR biopsies[tiab] OR Serology[tiab] OR Smear[tiab] OR Strip*[tiab] OR Microscop*[tiab] OR Immunoassay[tiab] OR “Lateral flow”[tiab] OR “reagent kit”[tiab] OR Diagnosis[tiab] OR Diagnostic*[tiab] OR “fluids and secretions”[MeSH Terms] OR “Diagnostic services”[MeSH Terms] **AND** | Target diagnostic technologies |
| “Point-of-care testing”[MeSH Terms] OR “Point of Care”[tiab] OR “Health care”[tiab] OR “Evaluation studies as topic”[MeSH Terms] OR “Evaluation studies”[PT] OR “Rapid Test”[tiab] OR “Quick test”[tiab] OR “Bedside test”[tiab] OR “Low Technology”[tiab] OR easy[tiab] OR Inexpensi*[tiab] OR Implementation[tiab] OR “Reproducibility of results”[MeSH Terms] OR “sensitivity and specificity”[MeSH Terms] OR Sensitivity[tiab] OR Specificity[tiab] OR Reliabilit*[tiab] OR Performance[tiab] OR Efficacy[tiab] OR Effectiv*[tiab] OR Efficient*[tiab] OR development[tiab] OR Evaluation[tiab] OR Reproducibility[tiab] OR Accuracy[tiab] OR Assessment[tiab] OR Cost[tiab] OR simple*[tiab] OR Simplicity[tiab] OR rapid*[tiab] OR quick[tiab] OR adapted[tiab] **AND** | Characteristics of Target diagnostic technologies |
| “Urban population”[MeSH Terms] OR “Residence characteristics”[MeSH Terms] OR “Urban health”[MeSH Terms] OR “Cities”[MeSH Terms] OR “Public housing”[MeSH Terms] OR “Urban*”[tiab] OR “Urban area”[tiab]OR “Urban settings”[tiab] OR “Urban health”[tiab] OR City[tiab] OR Periurban[tiab] OR city*[tiab] OR urban*[tiab] OR “urban health”[MeSH Terms] OR cities[MeSH Terms] OR “metropolitan area”[tiab] OR metropolitan*[tiab] OR “non rural”[tiab] OR downtown[tiab] OR midtown[tiab] OR uptown[tiab] OR district[tiab] OR slum[tiab] OR barrio[tiab] OR township[tiab] OR municipal*[tiab] OR civic[tiab] OR building*[tiab] OR suburban[tiab] OR town[tiab] OR “informal settlement”[tiab] OR neighborhood[tiab] OR neighbourhood[tiab] | Target population |

1.2 Search for Cochrane Library (Wiley)

| (“African tick bite fever”):ti,ab,kw OR (“African trypanosomiasis”):ti,ab,kw OR (Alphaviruse*):ti,ab,kw OR (“American trypanosomiasis”):ti,ab,kw OR (Arbovirus*):ti,ab,kw OR (arthropod borne disease*):ti,ab,kw OR (Arthropod-borne virus*):ti,ab,kw OR (bacterial NEXT disease*):ti,ab,kw OR (Bartonellosis):ti,ab,kw OR (Bilharzias*):ti,ab,kw OR [mh “Borrelia infections”] OR (Borrelia*):ti,ab,kw OR (“break-bone fever”):ti,ab,kw OR [mh “Boutonneuse fever”] OR [mh “Buruli ulcer”] OR (“buruli ulcer”):ti,ab,kw OR [mh “Chagas disease”] OR (“chagas disease”):ti,ab,kw OR [mh “Chikungunya virus”] OR [mh Chikungunya] OR (chikungunya):ti,ab,kw OR [mh Cholera] OR (cholera):ti,ab,kw OR (“Colorado tick fever”):ti,ab,kw OR [mh “Communicable diseases”] OR (communicable NEXT disease*):ti,ab,kw OR [mh “Communicable Diseases, Emerging”] OR [mh [Cysticercosis](https://www.ncbi.nlm.nih.gov/mesh/68003551)] OR (cysticercosis):ti,ab,kw OR (Crimean-congo NEXT haemorrhagic NEXT fever*):ti,ab,kw OR [mh Dengue] OR (dengue):ti,ab,kw OR [mh “[Hemorrhagic Fever, Ebola](https://www.ncbi.nlm.nih.gov/mesh/68019142)”] OR [mh Ebolavirus] OR (“ebola virus”):ti,ab,kw OR [mh [Echinococcosis](https://www.ncbi.nlm.nih.gov/mesh/68004443)] OR [mh “encephalitis, viral”] OR (encephalitis NEXT virus*):ti,ab,kw OR (“enteric fever”):ti,ab,kw OR (“Ehrlichia chaffeensis”):ti,ab,kw OR (“Ehrlichia ewingii”):ti,ab,kw OR (“erythema chronicum migrans”):ti,ab,kw OR (“erythema migrans”):ti,ab,kw OR [mh filariasis] OR [mh Elephantiasis] OR [mh “Elephantiasis, filarial”] OR (“filovirus diseases”):ti,ab,kw OR (“Flea borne spotted fever”):ti,ab,kw OR (“Food-borne trematodiases"):ti,ab,kw OR (“Francisella tularensis”):ti,ab,kw OR (“Hansen disease”):ti,ab,kw OR (“Hantavirus fever”):ti,ab,kw OR [mh “Hemorrhagic Fever Virus, Crimean-Congo”] OR [mh “Hemorrhagic fever, omsk”] OR [mh “Hendra virus”] OR (“Katayama fever”):ti,ab,kw OR (“Lassa fever”):ti,ab,kw OR (Leishman*):ti,ab,kw OR [mh Leprosy] OR [mh Leptospirosis] OR (Leptospir*):ti,ab,kw OR (Lyme*):ti,ab,kw OR [mh Malaria] OR (Marburg*):ti,ab,kw OR [mh “Neglected diseases”] OR (nipah):ti,ab,kw OR (Neuroschistosomias*):ti,ab,kw OR (“North asian tick typhus”):ti,ab,kw OR [mh Onchocerciasis] OR (Onchocerc*):ti,ab,kw OR (“Oriental spotted fever”):ti,ab,kw OR (“Orientia tsutsugamushi”):ti,ab,kw OR (“Pappataci fever”):ti,ab,kw OR (parasitic NEXT disease*):ti,ab,kw OR [mh “Paratyphoid fever”] OR [mh “Phlebotomus fever”] OR [mh “Phlebovirus”] OR [mh Plague] OR (Plague*):ti,ab,kw OR (Plasmodium):ti,ab,kw OR [mh “Coxiella burnetii”] OR (“Q fever”):ti,ab,kw OR [mh Rabies] OR (Rabies):ti,ab,kw OR (Re-emerging NEXT infectious NEXT disease*):ti,ab,kw OR (“Relapsing fever”):ti,ab,kw OR (“Remittent fever”):ti,ab,kw OR (Rickettsi*):ti,ab,kw OR [mh “Rickettsia infections”] OR [mh “Rift valley fever”] OR (“River Blindness”):ti,ab,kw OR (Robles NEXT Disease*):ti,ab,kw OR (“Rocky mountain spotted fever”):ti,ab,kw OR (“Salmonella typhi”):ti,ab,kw OR (“Salmonella enterica”):ti,ab,kw OR (“Sandfly fever”):ti,ab,kw OR [mh Schistosomiasis] OR (Schistosomia*):ti,ab,kw OR (Schistosoma*):ti,ab,kw OR (“Sicilian virus”):ti,ab,kw OR (“sleeping sickness”):ti,ab,kw OR (“Spotted fever”):ti,ab,kw OR (“three-day fever”):ti,ab,kw OR (“Toscana virus”):ti,ab,kw OR [mh Trachoma] OR (Trachoma):ti,ab,kw OR (“tick-borne infection”):ti,ab,kw OR (tick-borne NEXT viral NEXT disease*):ti,ab,kw OR (tick-borne NEXT bacterial NEXT disease*):ti,ab,kw OR (“tick borne parasite”):ti,ab,kw OR (Tick NEXT borne NEXT viral NEXT disease*):ti,ab,kw OR (Tropical NEXT disease*):ti,ab,kw OR (“Tropical neglected disease”):ti,ab,kw OR (Trypanosom*):ti,ab,kw OR [mh Tuberculosis] OR [mh Tularemia] OR [mh “Typhoid fever”] OR (Typhoid):ti,ab,kw OR (Typhus*):ti,ab,kw OR (“Vector borne infection”):ti,ab,kw OR (Vector-borne NEXT pathogen*):ti,ab,kw OR (“Viral encephalitis”):ti,ab,kw OR ("Virus hemorrhagic fever"):ti,ab,kw OR (“water borne disease”):ti,ab,kw OR [mh “Weil disease”] OR [mh “West Nile fever”] OR (“West Nile Virus”):ti,ab,kw OR (“West Nile neuroinvasive”):ti,ab,kw OR (“West Nile Meningitis”):ti,ab,kw OR (“West Nile meningoencephalitis”):ti,ab,kw OR (“West Nile poliomyelitis”):ti,ab,kw OR [mh “Yellow fever”] OR [mh “Yersinia pestis”] OR [mh “Zika virus infection”] OR (Zika*):ti,ab,kw OR (Zoonos*):ti,ab,kw OR (zoonotic NEXT pathogen*):ti,ab,kw **AND** | Target VBD and other infections |
| --- | --- |
| [mh “Diagnostic techniques and Procedures”] OR (“Urine sample”):ti,ab,kw OR (“Blood sample”);ti,ab,kw OR (“Saliva sample”):ti,ab,kw OR (“feces sample”):ti,ab,kw OR (“sputum sample”):ti,ab,kw OR (swab):ti,ab,kw OR (fingerprick):ti,ab,kw OR (biopsy):ti,ab,kw OR (biopsies):ti,ab,kw OR (Serology):ti,ab,kw OR (Smear):ti,ab,kw OR (Strip*):ti,ab,kw OR (Microscop*):ti,ab,kw OR (Immunoassay):ti,ab,kw OR (“Lateral flow”):ti,ab,kw OR (“reagent kit”):ti,ab,kw OR (Diagnosis):ti,ab,kw OR (Diagnostic*):ti,ab,kw OR [mh “fluids and secretions”] OR [mh “Diagnostic services”] **AND** | Target diagnostic technologies |
| [mh “Point-of-care testing”] OR (“Point of Care”):ti,ab,kw OR (“Health care”):ti,ab,kw OR [mh “Evaluation studies as topic”] OR (“Evaluation studies”):pt OR (“Rapid Test”):ti,ab,kw OR (“Quick test”):ti,ab,kw OR (“Bedside test”):ti,ab,kw OR (“Low Technology”):ti,ab,kw OR (easy):ti,ab,kw OR (Inexpensi*):ti,ab,kw OR (Implementation):ti,ab,kw OR [mh “Reproducibility of results”] OR [mh “sensitivity and specificity”] OR (Sensitivity):ti,ab,kw OR (Specificity):ti,ab,kw OR (Reliabilit*):ti,ab,kw OR (Performance):ti,ab,kw OR (Efficacy):ti,ab,kw OR (Effectiv*):ti,ab,kw OR (Efficient*):ti,ab,kw OR (development):ti,ab,kw OR (Evaluation):ti,ab,kw OR (Reproducibility):ti,ab,kw OR (Accuracy):ti,ab,kw OR (Assessment):ti,ab,kw OR (Cost):ti,ab,kw OR (simple*):ti,ab,kw OR (Simplicity):ti,ab,kw OR (rapid*):ti,ab,kw OR (quick):ti,ab,kw OR (adapted);ti,ab,kw **AND** | Characteristics of Target diagnostic technologies |
| [mh “Urban population”] OR [mh “Residence characteristics”] OR [mh “Urban health”] OR (urban health):ti,ab,kw OR [mh Cities] OR [mh “Public housing”] OR (Urban*):ti,ab,kw OR (“Urban area”):ti,ab,kw OR (“Urban settings”):ti,ab,kw OR (“Urban health”):ti,ab,kw OR (City):ti,ab,kw OR (Periurban):ti,ab,kw OR (city*):ti,ab,kw OR (urban*):ti,ab,kw OR [mh “urban health”] OR (“metropolitan area”):ti,ab,kw OR (metropolitan*):ti,ab,kw OR (“non rural”):ti,ab,kw OR (downtown):ti,ab,kw OR (midtown):ti,ab,kw OR (uptown):ti,ab,kw OR (district):ti,ab,kw OR (slum):ti,ab,kw OR (barrio):ti,ab,kw OR (township):ti,ab,kw OR (municipal*):ti,ab,kw OR (civic):ti,ab,kw OR (building*):ti,ab,kw OR (suburban):ti,ab,kw OR (town):ti,ab,kw OR (“informal settlement”):ti,ab,kw OR (neighborhood):ti,ab,kw OR (neighbourhood):ti,ab,kw | Target population |

1.3. Search for Embase:

| ‘African trypanosomiasis’/mj OR ‘Alphavirus infection’/mj OR ‘Arbovirus’/mj OR ‘Bacterial infection’/mj OR ‘Bartonellosis’/mj OR ‘Borrelia infection’/mj OR ‘Boutonneuse fever’/mj OR ‘Buruli ulcer’/mj OR ‘Chagas disease’/mj OR ‘Chikungunya virus’/mj OR ‘Chikungunya’/mj OR ‘Cholera’/mj OR ‘Vibrio cholerae’/mj OR ‘Colorado tick fever’/mj OR ‘Colorado tick fever virus’/mj OR ‘Communicable diseases’/mj OR ‘[Cysticercosis](https://www.ncbi.nlm.nih.gov/mesh/68003551)’/mj OR ‘Crimean-congo haemorrhagic fever’/mj OR ‘Crimean-congo haemorrhagic fever virus’/mj OR ‘Dengue’/mj OR ‘Ebola [Hemorrhagic Fever](https://www.ncbi.nlm.nih.gov/mesh/68019142)‘/mj OR ‘Ebolavirus’/mj OR ‘[Echinococcosis](https://www.ncbi.nlm.nih.gov/mesh/68004443)’/mj OR ‘virus encephalitis’/mj OR ‘Ehrlichiosis’/mj OR ‘erythema chronicum migrans’/mj OR ‘filariasis’/mj OR ‘lymphatic filariasis’/mj OR ‘filovirus infection’/mj OR ‘Francisella tularensis’/mj OR ‘tularemia’/mj OR ‘Hantavirus infection’/mj OR ‘Osmk Hemorrhagic fever’/mj OR ‘Hendra virus’/mj OR ‘Hendra virus infection’/mj OR ‘Lassa fever’/mj OR ‘Lassa fever virus’/mj OR ‘Leishmaniasis’/mj OR ‘Leprosy’/mj OR ‘Leptospirosis’/mj OR ‘Malaria’/mj OR ‘Neglected disease’/mj OR ‘Nipah Virus Infection’/mj OR ‘Neuroschistosomias’/mj OR ‘Onchocerciasis’/mj OR ‘Parasitosis’/mj OR ‘Paratyphoid fever’/mj OR ‘Phlebovirus’/mj OR ‘Plague’/mj OR ‘Coxiella burnetii’/mj OR ‘Q fever’/mj OR ‘Rabies’/mj OR ‘Rickettsia’/mj OR ‘Rift valley fever’/mj OR ‘Rocky mountain spotted fever’/mj OR ‘Sandfly fever’/mj OR ‘Schistosomiasis’/mj OR ‘Toscana virus’/mj OR ‘Trachoma’/mj OR ‘Tick borne encephalitis virus’/mj OR ‘Tuberculosis’/mj OR ‘Tularemia’/mj OR ‘Typhoid fever’/mj OR ‘Virus encephalitis’/mj OR ‘West Nile fever’/mj OR ‘Yellow fever’/mj OR ‘Yellow fever virus’/mj OR ‘Yersinia pestis’/mj OR ‘Zoonosis’/mj **AND** | Target VBD and other infections |
| --- | --- |
| ‘African tick bite fever’:ab,ti OR ‘African trypanosomiasis’:ab,ti OR ‘Alphaviruse*’:ab,ti OR ‘American trypanosomiasis’:ab,ti OR ‘Arbovirus*’:ab,ti OR ‘arthropod borne disease*’:ab,ti OR ‘Arthropod-borne virus*’:ab,ti OR ‘bacterial disease*’:ab,ti OR ‘Bartonellosis’:ab,ti OR ‘Bilharzi*’:ab,ti OR ‘borreliosis’:ab,ti OR ‘Borrelia*’:ab,ti OR ‘break-bone fever’:ab,ti OR ‘buruli ulcer’:ab,ti OR ‘chagas disease’:ab,ti OR ‘chikungunya’:ab,ti OR ‘cholera’:ab,ti OR ‘communicable disease*’:ab,ti OR ‘Communicable Diseases, Emerging’:ab,ti OR ‘cysticercosis’:ab,ti OR ‘dengue’:ab,ti OR ‘ebola virus’:ab,ti OR ‘enteric fever’:ab,ti OR ‘Ehrlichia chaffeensis’:ab,ti OR ‘Hansen disease’:ab,ti OR ‘Leishman*’:ab,ti OR ‘Leptospir*’:ab,ti OR ‘Lyme*’:ab,ti OR ‘Marburg*’:ab,ti OR ‘North asian tick typhus’:ab,ti OR Onchocerc*:ab,ti OR ‘Oriental spotted fever’:ab,ti OR ‘Orientia tsutsugamushi’:ab,ti OR ‘Pappataci fever’:ab,ti OR ‘parasitic disease*’:ab,ti OR ‘Phlebotomus fever’:ab,ti OR ‘Plague*’:ab,ti OR ‘Plasmodium’:ab,ti OR ‘Human Rabies’:ab,ti OR ‘Re emerging infectious disease*’:ab,ti OR ‘Relapsing fever’:ab,ti OR ‘Remittent fever’:ab,ti OR Rickettsi*:ab,ti OR ‘River Blindness’:ab,ti OR ‘Robles Disease*’:ab,ti OR ‘Salmonella typhi’:ab,ti OR ‘Salmonella enterica’:ab,ti OR ‘Schistosomia*’:ab,ti OR ‘Schistosoma*’:ab,ti OR ‘Sicilian virus’:ab,ti OR ‘sleeping sickness’:ab,ti OR ‘Spotted fever’:ab,ti OR ‘three-day fever’:ab,ti OR Trachoma:ab,ti OR ‘tick borne infection’:ab,ti OR ‘tick borne viral disease*’:ab,ti OR ‘tick borne bacterial disease*’:ab,ti OR ‘tick borne parasite’:ab,ti OR ‘Tick borne viral disease*’:ab,ti OR ‘Tropical disease*’:ab,ti OR ‘Tropical neglected disease’:ab,ti OR ‘Trypanosom*’:ab,ti OR ‘Typhoid’:ab,ti OR ‘Typhus*’:ab,ti OR ‘Vector borne infection’:ab,ti OR ‘Vector-borne pathogen*’:ab,ti OR ‘Virus hemorrhagic fever’:ab,ti OR ‘water borne disease’:ab,ti OR ‘Weil disease’:ab,ti OR ‘West Nile Virus’:ab,ti OR ‘West Nile neuroinvasive’:ab,ti OR ‘West Nile Meningitis’:ab,ti OR ‘West Nile meningoencephalitis’:ab,ti OR ‘West Nile poliomyelitis’:ab,ti OR Zika*:ab,ti OR Zoonos*:ab,ti OR ‘zoonotic pathogen*’:ab,ti **AND** | Target VBD and other infections |
| ‘Diagnostic Procedures’/mj OR ‘Body fluids and secretions’/mj OR ‘Diagnostic test’/mj **AND** | Target diagnostic technologies |
| ‘Urine sample’:ab,ti OR ‘Blood sample’:ab,ti OR ‘Saliva sample’:ab,ti OR ‘feces sample’:ab,ti OR ‘sputum sample’:ab,ti OR swab:ab,ti OR fingerprick:ab,ti OR biopsy:ab,ti OR Serology:ab,ti OR Smear:ab,ti OR Strip*:ab,ti OR Microscop*:ab,ti OR Immunoassay:ab,ti OR ‘Lateral flow’:ab,ti OR ‘reagent kit’:ab,ti OR Diagnosis:ab,ti OR Diagnostic*:ab,ti **AND** | Target diagnostic technologies |
| ‘Point of care testing’/mj OR ‘Health care’/mj OR ‘Evaluation study’/mj OR ‘Rapid Test’/mj OR ‘Reproducibility’/mj OR ‘sensitivity and specificity’/mj OR ‘Reliability’/mj OR ‘Performance’/mj **AND** | Characteristics of Target diagnostic technologies |
| ‘Point of Care’:ab,ti OR ‘Quick test’:ab,ti OR ‘Bedside test’:ab,ti OR ‘Low Technology’:ab,ti OR ‘easy’:ab,ti OR ‘Inexpensi*’:ab,ti OR ‘Implementation’:ab,ti OR ‘Sensitivity’:ab,ti OR ‘Specificity’:ab,ti OR ‘Efficacy’:ab,ti OR ‘development’:ab,ti OR ‘Evaluation’:ab,ti OR ‘Reproducibility’:ab,ti OR ‘Accuracy’:ab,ti OR ‘Assessment’:ab,ti OR ‘Cost’:ab,ti OR ‘simple*’:ab,ti OR ‘Rapid*’:ab,ti OR ‘quick’:ab,ti OR ‘adapted’:ab,ti **AND** | Characteristics of Target diagnostic technologies |
| ‘Urban population’/mj OR ‘Housing’/mj OR ‘Urban area’/mj OR ‘City’/mj OR ‘suburban area’/mj OR ‘neighborhood’/mj **AND** | Target population |
| ‘Residence characteristics’:ab,ti OR ‘Urban health’:ab,ti OR ‘Cities’:ab,ti OR ‘Urban*’:ab,ti OR ‘Urban settings’:ab,ti OR Periurban:ab,ti OR city*:ab,ti OR urban*:ab,ti OR ‘metropolitan area’:ab,ti OR metropolitan*:ab,ti OR ‘non rural’:ab,ti OR ‘downtown’:ab,ti OR ‘midtown’:ab,ti OR uptown:ab,ti OR district:ab,ti OR slum:ab,ti OR barrio:ab,ti OR township:ab,ti OR ‘city center’:ab,ti OR municipal*:ab,ti OR civic:ab,ti OR building:ab,ti OR town:ab,ti OR ‘informal settlement’:ab,ti OR Building:ab,ti OR neighbourhood:ab,ti | Target population |

1.4. Search for LILACS (LILACS BVS search by iAH form)

| ((TW African tick bite fever OR TW Fiebre por picadura de garrapata Africana OR TW Febre da picada de carrapato africano) OR (TW African trypanosomiasis OR TW Tripanosomiasis Africana OR TW Tripanossomíase Humana Africana) OR (TW Alphaviruse$) OR (TW American trypanosomiasis TW tripanossomíase americana OR TW tripanosomiasis americana) OR (TW Arbovirus$) OR (TW arthropod borne disease OR TW arthropod borne diseases OR TW Enfermedades transmitidas por artrópodos OR TW Enfermedad transmitida por artrópodo OR TW doenças transmitidas por artrópodes OR TW doenças associadas a artrópodes OR TW doenças causadas por artrópodes) OR (TW Arthropod-borne virus OR TW Arthropod-borne viruses OR TW virus transmitidos por artrópodos OR TW virus asociado a artrópodos OR TW vírus transmitido por artrópodes) OR (TW bacterial disease OR TW bacterial diseases OR TW Enfermedades bacterianas OR TW Enfermedad bacteriana OR TW doença bacteriana OR TW Doença bacteriana OR TW bacteri$) OR (TW Bartonellosis OR TW Bartonelosis) OR (TW Bilharzias$) OR (MH Borrelia infections OR MH Infecciones por Borrelia OR MH Infecções por Borrelia) OR (TW borreliosis OR TW borreliose) OR (TW Borreli$) OR (TW break-bone fever OR TW fiebre rompehuesos) OR (MH Boutonneuse fever OR MH Fiebre Botonosa OR MH Febre Botonosa) OR (MH Buruli ulcer OR MH Úlcera de Buruli) OR (TW Buruli ulcer OR TW Úlcera de Buruli) OR (MH Chagas disease OR MH Enfermedad de Chagas OR MH Doença de Chagas) OR (TW Chagas disease OR TW Enfermedad de Chagas OR TW Doença de Chagas) OR (MH Chikungunya virus OR MH Virus Chikungunya OR MH Vírus Chikungunya) OR (MH Chikungunya fever OR MH Fiebre Chikungunya OR MH Feber de Chikungunya) OR (TW Chikungunya) OR (MH Cholera OR MH Cólera) OR (TW Cholera OR TW cólera) OR (TW Colorado tick fever OR TW febre da carraça Colorado OR TW Fiebre por garrapatas de Colorado) OR (MH Communicable diseases OR MH Enfermedades transmisibles OR MH Doenças Transmissíveis) OR (TW Communicable diseases OR TW Enfermedades transmisibles OR TW Doenças Transmissíveis OR TW Infectious disease OR TW Enfermedades infecciosas OR TW doenças infecciosas) OR (MH Communicable Diseases, Emerging OR MH Enfermedades Transmisibles Emergentes OR MH Doenças Transmissíveis Emergentes) OR (MH [Cysticercosis](https://www.ncbi.nlm.nih.gov/mesh/68003551) OR MH Cisticercosis OR MH Cisticercose) OR (TW Cysticer$ OR TW Cisticerco$) OR (TW Crimean-congo haemorrhagic fever) OR (TW Crimean-congo haemorrhagic fever) OR (MH Dengue) OR (MH Dengue virus OR MH Virus del Dengue OR MH Vírus da Dengue) OR (TW Dengue) OR (MH Hemorrhagic Fever, Ebola OR MH Enfermedad por el Virus de Ebola OR Doença pelo Vírus Ebola) OR (MH Ebolavirus) OR (TW ebola) OR (MH [Echinococcosis](https://www.ncbi.nlm.nih.gov/mesh/68004443) OR MH Equinococosis OR MH Equinococose) OR (TW Echinoco$ OR TW Equinoco$ OR TW Hydatid Cyst OR Quiste hidatídico OR cisto hidático) OR (MH Encephalitis, Viral OR MH Encefalitis Viral OR MH Encefalite Viral) OR (TW Encephalitis virus OR TW Encephalitis viruses OR TW Encefalitis causada por virus OR TW Encefalite viral) OR (TW enteric fever OR TW febre entérica OR TW Fiebre entérica) OR (TW Ehrlichia chaffeensis OR TW Ehrlichia ewingii) OR (TW erythema chronicum migrans OR TW eritema crónico migratorio OR Eritema crónico migrans OR TW eritema crônico migratório) OR (TW erythema migrans OR TW eritema migrans) OR (MH filariasis OR MH filariose) OR (MH Elephantiasis OR MH Elefantiasis OR MH Elefantíase) OR (MH Elephantiasis, filarial OR MH Filariasis Linfática OR MH Filariose Linfática) OR (TW filari$) OR (TW filovirus diseases OR TW Enfermedades por filovirus OR TW filov$) OR (TW Flea borne spotted fever OR TW fiebre manchada OR TW febre maculosa) OR (TW Food-borne trematodiases OR TW trematodiasis de transmisión alimentaria OR TW trematodiases de origem alimentar OR Tremat$) OR (TW Francisella tularensis OR TW Tulare$) OR (TW Hansen disease OR TW enfermedad de Hansen OR TW doença de Hansen OR TW Hanseníase) OR (TW Hantavirus fever OR TW fiebre por Hantavirus OR TW Hantav$) OR (MH Hemorrhagic Fever, Crimean OR MH Fiebre Hemorrágica de Crimea OR MH Febre Hemorrágica da Crimeia OR MH Hemorrhagic Fever Virus, Crimean-Congo OR MH Virus de la Fiebre Hemorrágica de Crimea-Congo OR MH Vírus da Febre Hemorrágica da Crimeia-Congo) OR (MH Hemorrhagic Fever, Omsk OR MH Fiebre Hemorrágica de Omsk OR MH Febre Hemorrágica de Omsk) OR (MH Hendra virus OR MH Virus Hendra OR MH Vírus Hendra) OR (TW Katayama fever OR TW Febre de Katayama OR Fiebre de Katayama) OR (TW Lassa fever OR TW Fiebre de Lassa OR Febre de Lassa) OR (TW Leishman$) OR (MH Leprosy OR MH Lepra OR MH Hanseníase) OR (TW Lepr$) OR (MH Leptospirosis OR MH Leptospirose) OR (TW Leptospir$) OR (TW Lyme$) OR (MH Malaria OR MH Malária) OR (TW Malaria OR TW Malária) OR (TW Marburg$) OR (MH Neglected diseases OR MH Enfermedades desatendidas OR MH Doenças Negligenciadas) OR (TW nipah$) OR (TW Neuroschistosomias$ OR TW Neuroesquistos$ OR TW Schistosomias$ OR TW Esquistos$) OR (TW North Asian tick typhus OR TW Typh$ OR TW Tifo$ OR TW Tifus$) OR (MH Onchocerciasis OR MH Oncocercosis OR MH Oncocercose) OR (TW Onchocer$ OR TW Oncocer$) OR (TW Oriental spotted fever OR TW febre maculosa oriental OR Fiebre maculosa oriental) OR (TW Orientia tsutsugamushi) OR (TW Pappataci fever OR TW Fiebre Pappataci OR TW Febre Pappataci) OR (TW Parasitic disease OR TW parasit$) OR (MH Paratyphoid fever OR Fiebre paratifoidea OR Febre paratifoide) OR (TW paratyph$ OR TW Paratifo$) OR (MH Phlebotomus fever OR MH Fiebre por Flebótomos OR MH Febre por Flebótomos) OR (TW Phlebotomus OR TW Flebótomos) OR (MH Phlebovirus OR TW Phlebov$) OR (MH Plague OR MH Peste OR TW Plague OR TW Peste) OR (TW Plasmodium) OR (MH Coxiella burnetii OR TW Coxiella) OR (TW Q fever OR TW Fiebre Q OR TW Febre Q) OR (MH Rabies OR MH Rabia OR MH Raiva) OR (TW Rabies OR TW Rabia OR TW Raiva) OR (TW Re emerging infectious disease OR TW Enfermedades infecciosas re-emergentes OR TW doença infecciosa re-emergente) OR (TW Relapsing fever OR TW febre relapse OR TW Fiebre recurrente OR TW Rickett$ OR TW Borrel$) OR (TW Remittent fever OR TW Fiebre remitente OR TW Febre remitente) OR (MH Rickettsia infections OR MH Infecciones por Rickettsia OR MH Infecções por Rickettsia) OR (MH Rift valley fever OR MH Fiebre del valle del Rift OR MH Febre do Vale de Rift OR TW Rift) OR (TW River Blindness OR TW cegueira dos rios OR TW ceguera de los rios) OR (TW Robles disease OR TW doença de Robles OR TW Enfermedad de Robles) OR (TW Rocky mountain spotted fever OR TW Fiebre de las montañas rocosas OR TW Febre maculosa) OR (TW Salmonella typhi OR TW Salmonella enterica) OR (TW Sandfly fever) OR (MH Schistosomiasis) OR (TW Sicilian virus OR TW Virus siciliano OR TW Vírus siciliano) OR (TW sleeping sickness OR TW Enfermedad del sueño OR TW doença do sono) OR (TW Spotted fever OR TW Fiebre maculosa OR TW Fiebre manchada) OR (TW Toscana virus OR TW Vírus de Toscana OR TW Virus de Toscana) OR (TW three day fever OR TW Fiebre de los tres días) OR (MH Trachoma OR MH Tracoma OR TW Trachoma OR TW Tracoma) OR (TW tick-borne infection OR Enfermedades transmitidas por garrapatas OR TW Enfermedades asociadas a garrapatas OR TW doença transmitidas por carrapatos) OR (TW tick-borne bacterial disease) OR (TW tick borne parasite OR TW parasit$) OR (TW Tick borne viral disease) OR (TW Tropical disease OR TW Enfermedades tropicales OR TW doença tropical OR Tropical neglected disease) OR (TW Trypanosom$ OR TW Tripano$) OR (MH Tuberculosis OR MH Tuberculose OR TW Tuberculos$ OR MH Tularemia) OR (MH Typhoid fever OR MH Fiebre tifoidea OR MH Febre tifoide) OR (TW Vector borne infection OR Vector-borne pathogen OR TW Vector$ OR TW Vetor$) OR (TW Viral encephalitis OR TW Encefalitis viral OR TW Viral encefalite) OR (TW Virus hemorrhagic fever OR TW Virus de la fiebre hemorrágica OR TW vírus da febre hemorrágica) OR (TW water borne disease OR TW Enfermedades transmitidas por el agua OR TW doenças transmitidas pela água) OR (MH Weil Disease OR MH Enfermedad de Weil OR MH Doença de Weil OR TW Weil) OR (MH West Nile fever OR MH Fiebre del Nilo occidental OR MH Febre do Nilo Occidental OR TW West nile fever OR TW Nilo occidental) OR (TW West Nile neuroinvasive OR TW West Nile Meningitis OR TW West Nile meningoencephalitis OR TW West Nile poliomyelitis) OR (MH Yellow fever OR MH Fiebre amarilla OR MH febre amarela OR TW Yellow fever OR TW Fiebre amarilla OR TW febre amarela) OR (MH Yersinia pestis OR TW Yersinia) OR (MH Zika virus infection OR MH Infección por el virus Zika OR MH Infecção pelo Zika virus OR TW zika$) OR (TW Zoono$ OR TW Zoonó$)) **AND** | Target VBD and other infections |
| --- | --- |
| ((MH Diagnostic Techniques and Procedures OR MH Técnicas y Procedimientos Diagnósticos OR MH Técnicas e Procedimentos Diagnósticos) OR (TW Urine sample OR TW amostra de urina OR TW muestra de orina) OR (TW Blood sample OR TW muestra de sangre OR TW amostra de sangre) OR (TW saliva sample OR TW muestra de saliva OR TW amostra de saliva) OR (TW feces sample OR TW muestra de heces OR TW material fecal OR TW amostra de fezes) OR (TW sputum sample OR TW amostra de escarro OR TW muestra de esputo) OR (TW swab OR TW hisopado OR TW escobillón OR TW esfregão) OR (TW fingerprick TW lanceta OR TW puncionador OR TW pinchazo) OR (TW biops$ OR TW biópsias) OR (TW serology OR TW serología OR TW sorologia) OR (TW Smear OR TW borrão OR TW frotis) OR (TW strip$ TW tira TW tirilla) OR (TW Microscop$) OR (TW Immunoassay OR TW imunoensaio OR TW inmunoensayo) OR (TW lateral flow OR flujo lateral OR fluxo lateral) OR (TW reagent kit OR TW kit de reactivos OR TW kit de reagentes) OR (TW Diagnos$) OR (MH fluids and secretions OR MH Líquidos y Secreciones OR Líquidos e Secreções) OR (MH Diagnostic services OR MH Serviços de Diagnóstico OR MH Servicios de diagnóstico)) **AND** | Target diagnostic technologies |
| ((MH Point-of-care testing OR MH Pruebas en el punto de atención OR MH Testes imediatos) OR (TW Point of care OR TW Punto de atención OR TW Ponto de atendimiento) OR (TW Health care OR TW Cuidado de la salud OR TW Atención de salud OR TW cuidados de saúde OR TW Assistência à Saúde) OR (MH Evaluation Studies as topic OR MH Estudios de evaluación como asunto OR MH Estudos de Avaliação como Assunto) OR (PT Evaluation studies OR PT Estudios de evaluación OR PT estudos de avaliação) OR (TW Rapid Test OR TW Quick test TW Prueba rápida OR TW teste rápido) OR (TW Bedside test OR TW análisis clínico OR TW teste de cabeceira) OR (TW LowTechnology OR TW baixa tecnologia OR TW baja tecnología OR TW baja complejidad) OR (TW easy OR TW fácil) OR (TW Inexpensi$ OR TW Barato TW bajo costo) OR (TW Implementation OR TW implementação OR TW Implementación) OR (MH Reproducibility of results OR MH Reproducibilidad de los resultados OR MH Reprodutibilidade dos Testes) OR (MH Sensitivity and specificity OR MH Sensibilidad y Especificidad OR MH Sensibilidade e Especificidade) OR (TW Sensitivity OR TW Sensibilidad OR TW Sensibilidade) OR (TW Specificity OR TW Especificidad OR TW Especificidade) OR (TW Reliabili$ OR TW Reproducibility OR TW Reproducibilidad OR TW Confiabilidad OR TW confiança OR TW Reproducibilidade) OR (TW Performance OR TW atuação OR TW ejecución OR TW desempeño OR TW rendimiento OR TW Actuación) OR (TW Efficacy OR TW Eficacia OR TW Eficácia) OR (MH Efficacy OR MH Eficacia OR MH Eficácia) OR (TW Effectiv$ OR TW Efectiv$) OR (TW Efficient$ OR TW Eficien$) OR (TW development OR TW desarrollo OR TW desenvolvi$) OR (TW Evaluation OR TW Assessment OR TW Evaluación OR TW avaliação) OR (TW Accuracy OR TW precisão OR TW Precisión OR TW Exactitud) OR (TW Cost OR TW Costo OR TW Precio OR TW valor OR TW custo) OR (TW Simpl$ OR TW Sencillo OR TW Sencillez) OR (TW rapid$ OR TW quick OR TW rápido) OR (TW adapted OR TW adaptado)) **AND** | Characteristics of Target diagnostic technologies |
| ((MH Urban population OR MH Población urbana OR MH População Urbana) OR (MH Residence characteristics OR MH Distribución especial de la población OR MH Distribuição Espacial da População) OR (MH Urban health OR MH Salud urbana OR MH Saúde da População Urbana) OR (MH cities OR MH ciudades OR MH Cidades) OR (MH Suburban population OR MH Población suburban OR MH População Suburbana) OR (MH Public housing OR MH Vivienda popular OR MH Habitação Popular) OR (MH Metropolitan zones OR MH Zonas metropolitanas) OR (TW Urban$) OR (TW Urban area OR TW área urbana) OR (TW Urban settings OR TW Entornos urbanos OR TW ambientes urbanos) OR (TW Urban health OR TW Salud urbana OR TW saúde urbana) OR (TW City OR TW Ciudad OR TW Cidade) OR (TW Periurban$) OR (TW Metropolitan area OR TW área metropolitan) OR (TW metropolitan$) OR (TW non rural OR TW no rural) OR (TW downtown OR TW centro de la ciudad OR TW centro da cidade) OR (TW midtown) OR (TW uptown OR TW zona residencial) OR (TW distric OR TW distrito) OR (TW slum OR TW favela OR TW barrio bajo) OR (TW barrio OR TW neighborhood OR TW neighbourhood OR TW bairro) OR (TW township OR TW municipio OR TW municipalidade) OR (TW municipal$) OR (TW civic OR TW ciudadano OR TW civi$) OR (TW building$ OR TW edificio OR TW construcción OR TW prédio) OR (TW suburban OR TW suburban) OR (TW town OR TW vila OR TW pueblo) OR (TW informal settlement OR TW asentamiento informal OR TW assentamento informal)) | Target population |

1.5 Search for Global Health (Ovid):

| ((African tick bite fever.TI,AB.) OR (African trypanosomiasis.TI,AB.) OR (Alphaviruse$.TI,AB.) OR (American trypanosomiasis.TI,AB.) OR (Arbovirus$.TI,AB.) OR (arthropod borne disease$.TI,AB.) OR (Arthropod-borne virus$.TI,AB.) OR (bacterial disease$.TI,AB.) OR (Bartonellosis.TI,AB.) OR (Bilharzias$.TI,AB.) OR (Borrelia infections/) OR (borreliosis.TI,AB.) OR (Borreli$.TI,AB.) OR (break-bone fever.TI,AB.) OR (Boutonneuse fever/) OR (Boutonneuse.TI,AB.) OR (Buruli ulcer/) OR (buruli ulcer.TI,AB.) OR (Chagas disease/) OR (chagas disease.TI,AB.) OR (Chikungunya virus/) OR (Chikungunya/) OR (chikungunya.TI,AB.) OR (Cholera/) OR (cholera.TI,AB.) OR (Colorado tick fever.TI,AB.) OR (Communicable diseases/) OR (communicable disease$.TI,AB.) OR ([Cysticercosis](https://www.ncbi.nlm.nih.gov/mesh/68003551)/) OR (cysticerc$.TI,AB.) OR (Crimean-congo haemorrhagic fever$.TI,AB.) OR (Dengue/) OR (dengue.TI,AB.) OR ([Hemorrhagic Fever, Ebola](https://www.ncbi.nlm.nih.gov/mesh/68019142)/) OR (Ebolavirus/) OR (ebola.TI,AB.) OR ([Echinococcosis](https://www.ncbi.nlm.nih.gov/mesh/68004443)/) OR (encephalitis, viral/) OR (encephalitis virus$.TI,AB.) OR (enteric fever.TI,AB.) OR (Ehrlichia chaffeensis.TI,AB.) OR (Ehrlichi$.TI,AB.) OR (erythema chronicum migrans.TI,AB.) OR (erythema migrans.TI,AB.) OR (filariasis/) OR (Elephantiasis/) OR (Elephantiasis, filarial/) OR (filovirus diseases.TI,AB.) OR (Flea borne spotted fever.TI,AB.) OR (Food-borne trematodiases.TI,AB.) OR (Francisella tularensis.TI,AB.) OR (Hansen disease.TI,AB.) OR (Hantavirus fever.TI,AB.) OR (Hemorrhagic Fever Virus, Crimean-Congo/) OR (Hemorrhagic fever, omsk/) OR (Hendra virus/) OR (Katayama fever.TI,AB.) OR (Lassa fever.TI,AB.) OR (Leishman$.TI,AB.) OR (Leprosy/) OR (Leptospirosis/) OR (Leptospir$.TI,AB.) OR (Lyme$.TI,AB.) OR (Malaria/) OR (Marburg$.TI,AB.) OR (Neglected diseases/) OR (nipah.TI,AB.) OR (Neuroschisto$.TI,AB.) OR (North asian tick typhus.TI,AB.) OR (Onchocerciasis/) OR (Onchocerc$.TI,AB.) OR (Oriental spotted fever.TI,AB.) OR (Orientia tsutsugamushi.TI,AB.) OR (Pappataci fever.TI,AB.) OR (parasitic disease$.TI,AB.) OR (Paratyphoid fever/) OR (Phlebotomus fever/) OR (Phlebovirus/) OR (Plague/) OR (Plague$.TI,AB.) OR (Plasmodium.TI,AB.) OR (Coxiella burnetii/) OR (Q fever.TI,AB.) OR (Rabies/) OR (Rabies.TI,AB.) OR (Re emerging infectious disease$.TI,AB.) OR (Relapsing fever.TI,AB.) OR (Remittent fever.TI,AB.) OR (Ricket$.TI,AB.) OR (Rickettsia infections/) OR (Rift valley fever/) OR (River Blindness.TI,AB.) OR (Robles Disease$.TI,AB.) OR (Rocky mountain spotted fever.TI,AB.) OR (Salmonella typhi.TI,AB.) OR (Salmonella enterica.TI,AB.) OR (Sandfly fever.TI,AB.) OR (Schistosomiasis/) OR (Schistosomia$.TI,AB.) OR (Schistosoma$.TI,AB.) OR (Sicilian virus.TI,AB.) OR (sleeping sickness.TI,AB.) OR (Spotted fever.TI,AB.) OR (three-day fever.TI,AB.) OR (Toscana virus.TI,AB.) OR (Trachoma/) OR (Trachoma.TI,AB.) OR (tick-borne infection.TI,AB.) OR (tick-borne viral disease$.TI,AB.) OR (tick-borne bacterial disease$.TI,AB.) OR (tick borne parasite.TI,AB.) OR (Tick borne viral disease$.TI,AB.) OR (Tropical disease$.TI,AB.) OR (Tropical neglected disease.TI,AB.) OR (Trypanosom$.TI,AB.) OR (Tuberculosis/) OR (Tularemia/) OR (Typhoid fever/) OR (Typhoid.TI,AB.) OR (Typhus$.TI,AB.) OR (Vector borne infection.TI,AB.) OR (Vector-borne pathogen$.TI,AB.) OR (Viral encephalitis.TI,AB.) OR (Virus hemorrhagic fever.TI,AB.) OR (water borne disease.TI,AB.) OR (Weil disease/) OR (West Nile fever/) OR (West Nile Virus.TI,AB.) OR (West Nile neuroinvasive.TI,AB.) OR (West Nile Meningitis.TI,AB.) OR (West Nile meningoencephalitis.TI,AB.) OR (West Nile poliomyelitis.TI,AB.) OR (Yellow fever/) OR (Yersinia pestis/) OR (Zika virus infection/) OR (Zika$.TI,AB.) OR (Zoonos$.TI,AB.) OR (zoonotic pathogen$.TI,AB.)) **AND** | Target VBD and other infections |
| --- | --- |
| ((Diagnostic techniques and Procedures/) OR (Urine sample.TI,AB.) OR (Blood sample.TI,AB.) OR (Saliva sample.TI,AB.) OR (feces sample.TI,AB.) OR (sputum sample.TI,AB.) OR (swab.TI,AB.) OR (fingerprick.TI,AB.) OR (biopsy.TI,AB.) OR (biopsies.TI,AB.) OR (Serology.TI,AB.) OR (Smear.TI,AB.) OR (Strip$.TI,AB.) OR (Microscop$.TI,AB.) OR (Immunoassay.TI,AB.) OR (Lateral flow.TI,AB.) OR (reagent kit.TI,AB.) OR (Diagnosis.TI,AB.) OR (Diagnostic$.TI,AB.) OR (fluids and secretions/) OR (Diagnostic services/)) **AND** | Target diagnostic technologies |
| ((Point of care.TI,AB.) OR (Health care.TI,AB.) OR (Evaluation studies as topic/) OR (Rapid Test.TI,AB.) OR (Quick test.TI,AB.) OR (Bedside test.TI,AB.) OR (Low Technology.TI,AB.) OR (easy.TI,AB.) OR (Inexpensi$.TI,AB.) OR (Implementation.TI,AB.) OR (Reproducibility of results/) OR (sensitivity and specificity/) OR (Sensitivity.TI,AB.) OR (Specificity.TI,AB.) OR (Reliabilit$.TI,AB.) OR (Performance.TI,AB.) OR (Efficacy.TI,AB.) OR (Effectiv$.TI,AB.) OR (Efficient$.TI,AB.) OR (development.TI,AB.) OR (Evaluation.TI,AB.) OR (Reproducibility.TI,AB.) OR (Accuracy.TI,AB.) OR (Assessment.TI,AB.) OR (Cost.TI,AB.) OR (simple$.TI,AB.) OR (Simplicity.TI,AB.) OR (rapid$.TI,AB.) OR (quick.TI,AB.) OR (adapted.TI,AB.)) **AND** | Characteristics of Target diagnostic technologies |
| ((Urban population/) OR (Residence characteristics/) OR (Urban health/) OR (Cities/) OR (Public housing/) OR (Urban$.TI,AB.) OR (Urban area.TI,AB.) OR (Urban settings.TI,AB.) OR (Urban health.TI,AB.) OR (Periurban.TI,AB.) OR (city$.TI,AB.) OR (urban$.TI,AB.) OR (urban health/) OR (cities/) OR (metropolitan area.TI,AB.) OR (metropolitan$.TI,AB.) OR (non rural.TI,AB.) OR (downtown.TI,AB.) OR (midtown.TI,AB.) OR (uptown.TI,AB.) OR (district.TI,AB.) OR (slum.TI,AB.) OR (barrio.TI,AB.) OR (township.TI,AB.) OR (municipal$.TI,AB.) OR (civic.TI,AB.) OR (building$.TI,AB.) OR (suburban.TI,AB.) OR (town.TI,AB.) OR (informal settlement.TI,AB.) OR (neighborhood.TI,AB.) OR (neighbourhood.TI,AB.)) | Target population |

1.6 Search for WHOLIS (BVS search by iAH form):

| ((TW African tick bite fever OR TW Fiebre por picadura de garrapata Africana OR TW Febre da picada de carrapato africano) OR (TW African trypanosomiasis OR TW Tripanosomiasis Africana OR TW Tripanossomíase Humana Africana) OR (TW Alphaviruse$) OR (TW American trypanosomiasis TW tripanossomíase americana OR TW tripanosomiasis americana) OR (TW Arbovirus$) OR (TW arthropod borne disease OR TW arthropod borne diseases OR TW Enfermedades transmitidas por artrópodos OR TW Enfermedad transmitida por artrópodo OR TW doenças transmitidas por artrópodes OR TW doenças associadas a artrópodes OR TW doenças causadas por artrópodes) OR (TW Arthropod-borne virus OR TW Arthropod-borne viruses OR TW virus transmitidos por artrópodos OR TW virus asociado a artrópodos OR TW vírus transmitido por artrópodes) OR (TW bacterial disease OR TW bacterial diseases OR TW Enfermedades bacterianas OR TW Enfermedad bacteriana OR TW doença bacteriana OR TW Doença bacteriana OR TW bacteri$) OR (TW Bartonellosis OR TW Bartonelosis) OR (TW Bilharzias$) OR (MH Borrelia infections OR MH Infecciones por Borrelia OR MH Infecções por Borrelia) OR (TW borreliosis OR TW borreliose) OR (TW Borreli$) OR (TW break-bone fever OR TW fiebre rompehuesos) OR (MH Boutonneuse fever OR MH Fiebre Botonosa OR MH Febre Botonosa) OR (MH Buruli ulcer OR MH Úlcera de Buruli) OR (TW Buruli ulcer OR TW Úlcera de Buruli) OR (MH Chagas disease OR MH Enfermedad de Chagas OR MH Doença de Chagas) OR (TW Chagas disease OR TW Enfermedad de Chagas OR TW Doença de Chagas) OR (MH Chikungunya virus OR MH Virus Chikungunya OR MH Vírus Chikungunya) OR (MH Chikungunya fever OR MH Fiebre Chikungunya OR MH Feber de Chikungunya) OR (TW Chikungunya) OR (MH Cholera OR MH Cólera) OR (TW Cholera OR TW cólera) OR (TW Colorado tick fever OR TW febre da carraça Colorado OR TW Fiebre por garrapatas de Colorado) OR (MH Communicable diseases OR MH Enfermedades transmisibles OR MH Doenças Transmissíveis) OR (TW Communicable diseases OR TW Enfermedades transmisibles OR TW Doenças Transmissíveis OR TW Infectious disease OR TW Enfermedades infecciosas OR TW doenças infecciosas) OR (MH Communicable Diseases, Emerging OR MH Enfermedades Transmisibles Emergentes OR MH Doenças Transmissíveis Emergentes) OR (MH [Cysticercosis](https://www.ncbi.nlm.nih.gov/mesh/68003551) OR MH Cisticercosis OR MH Cisticercose) OR (TW Cysticer$ OR TW Cisticerco$) OR (TW Crimean-congo haemorrhagic fever) OR (TW Crimean-congo haemorrhagic fever) OR (MH Dengue) OR (MH Dengue virus OR MH Virus del Dengue OR MH Vírus da Dengue) OR (TW Dengue) OR (MH Hemorrhagic Fever, Ebola OR MH Enfermedad por el Virus de Ebola OR Doença pelo Vírus Ebola) OR (MH Ebolavirus) OR (TW ebola) OR (MH [Echinococcosis](https://www.ncbi.nlm.nih.gov/mesh/68004443) OR MH Equinococosis OR MH Equinococose) OR (TW Echinoco$ OR TW Equinoco$ OR TW Hydatid Cyst OR Quiste hidatídico OR cisto hidático) OR (MH Encephalitis, Viral OR MH Encefalitis Viral OR MH Encefalite Viral) OR (TW Encephalitis virus OR TW Encephalitis viruses OR TW Encefalitis causada por virus OR TW Encefalite viral) OR (TW enteric fever OR TW febre entérica OR TW Fiebre entérica) OR (TW Ehrlichia chaffeensis OR TW Ehrlichia ewingii) OR (TW erythema chronicum migrans OR TW eritema crónico migratorio OR Eritema crónico migrans OR TW eritema crônico migratório) OR (TW erythema migrans OR TW eritema migrans) OR (MH filariasis OR MH filariose) OR (MH Elephantiasis OR MH Elefantiasis OR MH Elefantíase) OR (MH Elephantiasis, filarial OR MH Filariasis Linfática OR MH Filariose Linfática) OR (TW filari$) OR (TW filovirus diseases OR TW Enfermedades por filovirus OR TW filov$) OR (TW Flea borne spotted fever OR TW fiebre manchada OR TW febre maculosa) OR (TW Food-borne trematodiases OR TW trematodiasis de transmisión alimentaria OR TW trematodiases de origem alimentar OR Tremat$) OR (TW Francisella tularensis OR TW Tulare$) OR (TW Hansen disease OR TW enfermedad de Hansen OR TW doença de Hansen OR TW Hanseníase) OR (TW Hantavirus fever OR TW fiebre por Hantavirus OR TW Hantav$) OR (MH Hemorrhagic Fever, Crimean OR MH Fiebre Hemorrágica de Crimea OR MH Febre Hemorrágica da Crimeia OR MH Hemorrhagic Fever Virus, Crimean-Congo OR MH Virus de la Fiebre Hemorrágica de Crimea-Congo OR MH Vírus da Febre Hemorrágica da Crimeia-Congo) OR (MH Hemorrhagic Fever, Omsk OR MH Fiebre Hemorrágica de Omsk OR MH Febre Hemorrágica de Omsk) OR (MH Hendra virus OR MH Virus Hendra OR MH Vírus Hendra) OR (TW Katayama fever OR TW Febre de Katayama OR Fiebre de Katayama) OR (TW Lassa fever OR TW Fiebre de Lassa OR Febre de Lassa) OR (TW Leishman$) OR (MH Leprosy OR MH Lepra OR MH Hanseníase) OR (TW Lepr$) OR (MH Leptospirosis OR MH Leptospirose) OR (TW Leptospir$) OR (TW Lyme$) OR (MH Malaria OR MH Malária) OR (TW Malaria OR TW Malária) OR (TW Marburg$) OR (MH Neglected diseases OR MH Enfermedades desatendidas OR MH Doenças Negligenciadas) OR (TW nipah$) OR (TW Neuroschistosomias$ OR TW Neuroesquistos$ OR TW Schistosomias$ OR TW Esquistos$) OR (TW North Asian tick typhus OR TW Typh$ OR TW Tifo$ OR TW Tifus$) OR (MH Onchocerciasis OR MH Oncocercosis OR MH Oncocercose) OR (TW Onchocer$ OR TW Oncocer$) OR (TW Oriental spotted fever OR TW febre maculosa oriental OR Fiebre maculosa oriental) OR (TW Orientia tsutsugamushi) OR (TW Pappataci fever OR TW Fiebre Pappataci OR TW Febre Pappataci) OR (TW Parasitic disease OR TW parasit$) OR (MH Paratyphoid fever OR Fiebre paratifoidea OR Febre paratifoide) OR (TW paratyph$ OR TW Paratifo$) OR (MH Phlebotomus fever OR MH Fiebre por Flebótomos OR MH Febre por Flebótomos) OR (TW Phlebotomus OR TW Flebótomos) OR (MH Phlebovirus OR TW Phlebov$) OR (MH Plague OR MH Peste OR TW Plague OR TW Peste) OR (TW Plasmodium) OR (MH Coxiella burnetii OR TW Coxiella) OR (TW Q fever OR TW Fiebre Q OR TW Febre Q) OR (MH Rabies OR MH Rabia OR MH Raiva) OR (TW Rabies OR TW Rabia OR TW Raiva) OR (TW Re emerging infectious disease OR TW Enfermedades infecciosas re-emergentes OR TW doença infecciosa re-emergente) OR (TW Relapsing fever OR TW febre relapse OR TW Fiebre recurrente OR TW Rickett$ OR TW Borrel$) OR (TW Remittent fever OR TW Fiebre remitente OR TW Febre remitente) OR (MH Rickettsia infections OR MH Infecciones por Rickettsia OR MH Infecções por Rickettsia) OR (MH Rift valley fever OR MH Fiebre del valle del Rift OR MH Febre do Vale de Rift OR TW Rift) OR (TW River Blindness OR TW cegueira dos rios OR TW ceguera de los rios) OR (TW Robles disease OR TW doença de Robles OR TW Enfermedad de Robles) OR (TW Rocky mountain spotted fever OR TW Fiebre de las montañas rocosas OR TW Febre maculosa) OR (TW Salmonella typhi OR TW Salmonella enterica) OR (TW Sandfly fever) OR (MH Schistosomiasis) OR (TW Sicilian virus OR TW Virus siciliano OR TW Vírus siciliano) OR (TW sleeping sickness OR TW Enfermedad del sueño OR TW doença do sono) OR (TW Spotted fever OR TW Fiebre maculosa OR TW Fiebre manchada) OR (TW Toscana virus OR TW Vírus de Toscana OR TW Virus de Toscana) OR (TW three day fever OR TW Fiebre de los tres días) OR (MH Trachoma OR MH Tracoma OR TW Trachoma OR TW Tracoma) OR (TW tick-borne infection OR Enfermedades transmitidas por garrapatas OR TW Enfermedades asociadas a garrapatas OR TW doença transmitidas por carrapatos) OR (TW tick-borne bacterial disease) OR (TW tick borne parasite OR TW parasit$) OR (TW Tick borne viral disease) OR (TW Tropical disease OR TW Enfermedades tropicales OR TW doença tropical OR Tropical neglected disease) OR (TW Trypanosom$ OR TW Tripano$) OR (MH Tuberculosis OR MH Tuberculose OR TW Tuberculos$ OR MH Tularemia) OR (MH Typhoid fever OR MH Fiebre tifoidea OR MH Febre tifoide) OR (TW Vector borne infection OR Vector-borne pathogen OR TW Vector$ OR TW Vetor$) OR (TW Viral encephalitis OR TW Encefalitis viral OR TW Viral encefalite) OR (TW Virus hemorrhagic fever OR TW Virus de la fiebre hemorrágica OR TW vírus da febre hemorrágica) OR (TW water borne disease OR TW Enfermedades transmitidas por el agua OR TW doenças transmitidas pela água) OR (MH Weil Disease OR MH Enfermedad de Weil OR MH Doença de Weil OR TW Weil) OR (MH West Nile fever OR MH Fiebre del Nilo occidental OR MH Febre do Nilo Occidental OR TW West nile fever OR TW Nilo occidental) OR (TW West Nile neuroinvasive OR TW West Nile Meningitis OR TW West Nile meningoencephalitis OR TW West Nile poliomyelitis) OR (MH Yellow fever OR MH Fiebre amarilla OR MH febre amarela OR TW Yellow fever OR TW Fiebre amarilla OR TW febre amarela) OR (MH Yersinia pestis OR TW Yersinia) OR (MH Zika virus infection OR MH Infección por el virus Zika OR MH Infecção pelo Zika virus OR TW zika$) OR (TW Zoono$ OR TW Zoonó$)) **AND** | Target VBD and other infections |
| --- | --- |
| ((MH Diagnostic Techniques and Procedures OR MH Técnicas y Procedimientos Diagnósticos OR MH Técnicas e Procedimentos Diagnósticos) OR (TW Urine sample OR TW amostra de urina OR TW muestra de orina) OR (TW Blood sample OR TW muestra de sangre OR TW amostra de sangre) OR (TW saliva sample OR TW muestra de saliva OR TW amostra de saliva) OR (TW feces sample OR TW muestra de heces OR TW material fecal OR TW amostra de fezes) OR (TW sputum sample OR TW amostra de escarro OR TW muestra de esputo) OR (TW swab OR TW hisopado OR TW escobillón OR TW esfregão) OR (TW fingerprick TW lanceta OR TW puncionador OR TW pinchazo) OR (TW biops$ OR TW biópsias) OR (TW serology OR TW serología OR TW sorologia) OR (TW Smear OR TW borrão OR TW frotis) OR (TW strip$ TW tira TW tirilla) OR (TW Microscop$) OR (TW Immunoassay OR TW imunoensaio OR TW inmunoensayo) OR (TW lateral flow OR flujo lateral OR fluxo lateral) OR (TW reagent kit OR TW kit de reactivos OR TW kit de reagentes) OR (TW Diagnos$) OR (MH fluids and secretions OR MH Líquidos y Secreciones OR Líquidos e Secreções) OR (MH Diagnostic services OR MH Serviços de Diagnóstico OR MH Servicios de diagnóstico)) **AND** | Target diagnostic technologies |
| ((MH Point-of-care testing OR MH Pruebas en el punto de atención OR MH Testes imediatos) OR (TW Point of care OR TW Punto de atención OR TW Ponto de atendimiento) OR (TW Health care OR TW Cuidado de la salud OR TW Atención de salud OR TW cuidados de saúde OR TW Assistência à Saúde) OR (MH Evaluation Studies as topic OR MH Estudios de evaluación como asunto OR MH Estudos de Avaliação como Assunto) OR (PT Evaluation studies OR PT Estudios de evaluación OR PT estudos de avaliação) OR (TW Rapid Test OR TW Quick test TW Prueba rápida OR TW teste rápido) OR (TW Bedside test OR TW análisis clínico OR TW teste de cabeceira) OR (TW LowTechnology OR TW baixa tecnologia OR TW baja tecnología OR TW baja complejidad) OR (TW easy OR TW fácil) OR (TW Inexpensi$ OR TW Barato TW bajo costo) OR (TW Implementation OR TW implementação OR TW Implementación) OR (MH Reproducibility of results OR MH Reproducibilidad de los resultados OR MH Reprodutibilidade dos Testes) OR (MH Sensitivity and specificity OR MH Sensibilidad y Especificidad OR MH Sensibilidade e Especificidade) OR (TW Sensitivity OR TW Sensibilidad OR TW Sensibilidade) OR (TW Specificity OR TW Especificidad OR TW Especificidade) OR (TW Reliabili$ OR TW Reproducibility OR TW Reproducibilidad OR TW Confiabilidad OR TW confiança OR TW Reproducibilidade) OR (TW Performance OR TW atuação OR TW ejecución OR TW desempeño OR TW rendimiento OR TW Actuación) OR (TW Efficacy OR TW Eficacia OR TW Eficácia) OR (MH Efficacy OR MH Eficacia OR MH Eficácia) OR (TW Effectiv$ OR TW Efectiv$) OR (TW Efficient$ OR TW Eficien$) OR (TW development OR TW desarrollo OR TW desenvolvi$) OR (TW Evaluation OR TW Assessment OR TW Evaluación OR TW avaliação) OR (TW Accuracy OR TW precisão OR TW Precisión OR TW Exactitud) OR (TW Cost OR TW Costo OR TW Precio OR TW valor OR TW custo) OR (TW Simpl$ OR TW Sencillo OR TW Sencillez) OR (TW rapid$ OR TW quick OR TW rápido) OR (TW adapted OR TW adaptado)) **AND** | Characteristics of Target diagnostic technologies |
| ((MH Urban population OR MH Población urbana OR MH População Urbana) OR (MH Residence characteristics OR MH Distribución especial de la población OR MH Distribuição Espacial da População) OR (MH Urban health OR MH Salud urbana OR MH Saúde da População Urbana) OR (MH cities OR MH ciudades OR MH Cidades) OR (MH Suburban population OR MH Población suburban OR MH População Suburbana) OR (MH Public housing OR MH Vivienda popular OR MH Habitação Popular) OR (MH Metropolitan zones OR MH Zonas metropolitanas) OR (TW Urban$) OR (TW Urban area OR TW área urbana) OR (TW Urban settings OR TW Entornos urbanos OR TW ambientes urbanos) OR (TW Urban health OR TW Salud urbana OR TW saúde urbana) OR (TW City OR TW Ciudad OR TW Cidade) OR (TW Periurban$) OR (TW Metropolitan area OR TW área metropolitan) OR (TW metropolitan$) OR (TW non rural OR TW no rural) OR (TW downtown OR TW centro de la ciudad OR TW centro da cidade) OR (TW midtown) OR (TW uptown OR TW zona residencial) OR (TW distric OR TW distrito) OR (TW slum OR TW favela OR TW barrio bajo) OR (TW barrio OR TW neighborhood OR TW neighbourhood OR TW bairro) OR (TW township OR TW municipio OR TW municipalidade) OR (TW municipal$) OR (TW civic OR TW ciudadano OR TW civi$) OR (TW building$ OR TW edificio OR TW construcción OR TW prédio) OR (TW suburban OR TW suburban) OR (TW town OR TW vila OR TW pueblo) OR (TW informal settlement OR TW asentamiento informal OR TW assentamento informal)) | Target population |

1.7 Search for Opengray:

| ((abstract:African tick bite fever) OR (abstract:African trypanosomiasis) OR (abstract:Alphaviruse*) OR (abstract:American trypanosomiasis) OR (abstract:Arbovirus*) OR (abstract:arthropod borne disease) OR (abstract:Arthropod-borne virus) OR (abstract:bacterial disease) OR (abstract:bacteria*) OR (abstract:bartonellosis) OR (abstract:Bilharzias*) OR (subject:“Borrelia infections”) OR (abstract:borreliosis) OR (abstract:Borrelia*) OR (abstract:break bone fever) OR (subject:“Boutonneuse fever”) OR (abstract:Boutonneuse fever) OR (subject:“Buruli ulcer”) OR (abstract:“buruli ulcer”) OR (subject:“Chagas disease”) OR (abstract:“chagas disease”) OR (subject:“Chikungunya virus”) OR (subject:Chikungunya) OR (abstract:chikungunya) OR (subject:Cholera) OR (abstract:cholera) OR (abstract:“Colorado tick fever”) OR (subject:“Communicable diseases”) OR (abstract:communicable disease) OR (subject:“Communicable Diseases, Emerging”) OR (subject:[Cysticercosis](https://www.ncbi.nlm.nih.gov/mesh/68003551)) OR (abstract:cysticerc*) OR (abstract:“Crimean-congo haemorrhagic fever) OR (subject:Dengue) OR (abstract:dengue) OR (subject:“[Hemorrhagic Fever, Ebola](https://www.ncbi.nlm.nih.gov/mesh/68019142)”) OR (subject:Ebolavirus) OR (abstract:ebola) OR (subject:[Echinococcosis](https://www.ncbi.nlm.nih.gov/mesh/68004443)) OR (subject:“encephalitis, viral”) OR (abstract:encephalitis virus) OR (abstract:echinoco*) OR (abstract:enteric fever) OR (abstract:Ehrlichia*) OR (abstract:“erythema chronicum migrans”) OR (abstract:“erythema migrans”) OR (subject:filariasis) OR (subject:Elephantiasis) OR (subject:“Elephantiasis, filarial”) OR (abstract:filaria*) OR (abstract:“filovirus disease”) OR (abstract:“Flea borne spotted fever”) OR (abstract:Trematod*) OR (abstract:“Francisella tularensis”) OR (abstract:“Hansen disease”) OR (abstract:“Hantavirus fever”) OR (subject:“Hemorrhagic Fever Virus, Crimean-Congo”) OR (subject:“Hemorrhagic fever, omsk”) OR (abstract:Hemorrhagic fever, omsk) OR (subject:“Hendra virus”) OR (abstract:Hendra virus) OR (abstract:“Katayama fever”) OR (abstract:“Lassa fever”) OR (abstract:Leishman*) OR (subject:Leprosy) OR (subject:Leptospirosis) OR (abstract:lepr*) OR (abstract:Leptospir*) OR (abstract:Lyme*) OR (subject:Malaria) OR (abstract:malaria) OR (abstract:Marburg*) OR (subject:“Neglected diseases”) OR (abstract:nipah*) OR (abstract:Neuroschistosomias*) OR (abstract:schistos*) OR (abstract:“North asian tick typhus”) OR (subject:Onchocerciasis) OR (abstract:Onchocerc*) OR (abstract:“Oriental spotted fever”) OR (abstract:“Orientia tsutsugamushi”) OR (abstract:“Pappataci fever”) OR (abstract:“parasitic disease*) OR (abstract:parasit*) OR (subject:“Paratyphoid fever”) OR (abstract:paratyph*) OR (subject:”Phlebotomus fever”) OR (abstract:phlebotomus) OR (subject:“Phlebovirus”) OR (abstract:Phlebovirus) OR (subject:Plague) OR (abstract:Plague*) OR (abstract:Plasmodium) OR (subject:“Coxiella burnetii”) OR (abstract:coxiella) OR (abstract:“Q fever”) OR (subject:Rabies) OR (abstract:Rabie*) OR (abstract:Re emerging infectious disease) OR (abstract:Relapsing fever) OR (abstract:Remittent fever) OR (abstract:Rickett*) OR (subject:“Rickettsia infections”) OR (subject:“Rift valley fever”) OR (abstract:Rift) OR (abstract:“River Blindness”) OR (abstract:Robles Disease*) OR (abstract:Rocky mountain spotted fever) OR (abstract:Salmonella typhi) OR (abstract:typh*) OR (abstract:“Salmonella enterica”) OR (abstract:“Sandfly fever”) OR (subject:“Schistosomiasis”) OR (abstract:“Sicilian virus”) OR (abstract:“sleeping sickness”) OR (abstract:“Spotted fever”) OR (abstract:“three-day fever”) OR (abstract:“Toscana virus”) OR (subject:Trachoma) OR (abstract:Trachoma) OR (abstract:“tick-borne infection”) OR (abstract:“tick borne viral disease”) OR (abstract:“tick-borne bacterial disease”) OR (abstract:“tick borne parasite”) OR (abstract:“Tropical disease”) OR (abstract:“Tropical neglected disease”) OR (abstract:Trypanosom*) OR (subject:Tuberculosis) OR (abstract:tuberculosis) OR (subject:Tularemia) OR (subject:“Typhoid fever”) OR (abstract:Typhoid) OR (abstract:Typhus*) OR (abstract:“Vector borne infection”) OR (abstract:“Vector-borne pathogen”) OR (abstract:vector) OR (abstract:“Viral encephalitis”) OR (abstract:"Virus hemorrhagic fever") OR (abstract:“water borne disease”) OR (subject:“Weil disease”) OR (abstract:Weil) OR (subject:“West Nile fever”) OR (abstract:West Nile Virus) OR (abstract:West Nile neuroinvasive) OR (abstract:West Nile Meningitis) OR (abstract:West Nile meningoencephalitis) OR (abstract:“West Nile poliomyelitis”) OR (subject:“Yellow fever”) OR (abstract:Yellow fever) OR (subject:“Yersinia pestis”) OR (abstract:Yersinia) OR (subject:“Zika virus infection”) OR (abstract:Zika*) OR (abstract:Zoono*) OR (abstract:zoonotic pathogen)) **AND** | Target VBD and other infections |
| --- | --- |
| ((subject:“Diagnostic techniques and Procedures”) OR (abstract:Diagnostic techniques and Procedures) OR (abstract:Urine sample) OR (abstract:Blood sample) OR (abstract:Saliva sample) OR (abstract:feces sample) OR (abstract:sputum sample) OR (abstract:swab) OR (abstract:fingerprick) OR (abstract:biopsy) OR (abstract:biopsies) OR (abstract:Serology) OR (abstract:Smear) OR (abstract:Strip*) OR (abstract:Microscop*) OR (abstract:Immunoassay) OR (abstract:“Lateral flow”) OR (abstract:“reagent kit”) OR (abstract:Diagnos*) OR (subject:“fluids and secretions”) OR (abstract:fluids and secretions) OR (subject:”Diagnostic services”) OR (abstract:Diagnostic services)) **AND** | Target diagnostic technologies |
| ((subject:”Point-of-care testing”) OR (abstract:Point of care testing) OR (abstract:”Health care”) OR (subject:“Evaluation studies as topic”) OR (abstract:“Evaluation studies”) OR (abstract:“Rapid Test”) OR (abstract:“Quick test”) OR (abstract:“Bedside test”) OR (abstract:“Low Technology”) OR (abstract:easy) OR (abstract:Inexpensi*) OR (abstract:Implementation) OR (subject:“Reproducibility of results”) OR (abstract:reproducibility) OR (subject:“sensitivity and specificity”) OR (abstract:Sensitivity) OR (abstract:Specificity) OR (abstract:Reliabilit*) OR (abstract:Performance) OR (abstract:Efficacy) OR (abstract:Effectiv*) OR (abstract:Efficient*) OR (abstract:development) OR (abstract:Evaluation) OR (abstract:Accuracy) OR (abstract:Assessment) OR (abstract:Cost) OR (abstract:simpl*) OR (abstract:Simplicity) OR (abstract:rapid*) OR (abstract:quick) OR (abstract:adapted)) **AND** | Characteristics of Target diagnostic technologies |
| ((subject:”Urban population”) OR (abstract:urban population) OR (subject:“Residence characteristics”) OR (abstract:residence characteristics) OR (subject:“Urban health”) OR (abstract:urban health OR subject:”Cities”) OR (abstract:city) OR (abstract:cities) OR (abstract:Public housing) OR (subject:”Public housing”) OR (abstract:urban*) OR (abstract:urban area) OR (abstract:urban settings) OR (abstract:periurban) OR (abstract:city*) OR (abstract:”metropolitan area”) OR (abstract:metropolitan*) OR (abstract:“non rural”) OR (abstract:downtown) OR (abstract:midtown) OR (abstract:uptown) OR (abstract:district) OR (abstract:slum) OR (abstract:barrio) OR (abstract:township) OR (abstract:municipal*) OR (abstract:civic) OR (abstract:building*) OR (abstract:suburban) OR (abstract:town) OR (abstract:“informal settlement”) OR (abstract:neighborhood) OR (abstract:neighbourhood)) | Target population |

1.8 Search for Scopus

| ((TITLE-ABS-KEY: (African tick bite fever)) OR (TITLE-ABS-KEY: (African trypanosomiasis)) OR (TITLE-ABS-KEY: (Alphaviruse*)) OR (TITLE-ABS-KEY: (American trypanosomiasis)) OR (TITLE-ABS-KEY: (Arbovirus*)) OR (TITLE-ABS-KEY: (arthropod borne disease)) OR (TITLE-ABS-KEY: (Arthropod-borne virus)) OR (TITLE-ABS-KEY: (bacterial disease)) OR (TITLE-ABS-KEY: (bacteria*)) OR (TITLE-ABS-KEY: (bartonellosis)) OR (TITLE-ABS-KEY: (Bilharzias*)) OR (INDEXTERMS: (“Borrelia infections”)) OR (TITLE-ABS-KEY: (borreliosis)) OR (TITLE-ABS-KEY: (Borrelia*)) OR (TITLE-ABS-KEY: (break bone fever)) OR (INDEXTERMS: (“Boutonneuse fever”)) OR (TITLE-ABS-KEY: (Boutonneuse fever)) OR (INDEXTERMS: (“Buruli ulcer”)) OR (TITLE-ABS-KEY: (“buruli ulcer”)) OR (INDEXTERMS: (“Chagas disease”)) OR (TITLE-ABS-KEY: (“chagas disease”)) OR (INDEXTERMS: (“Chikungunya virus”)) OR (INDEXTERMS: (Chikungunya)) OR (TITLE-ABS-KEY: (chikungunya)) OR (INDEXTERMS: (Cholera)) OR (TITLE-ABS-KEY: (cholera)) OR (TITLE-ABS-KEY: (“Colorado tick fever”)) OR (INDEXTERMS: (“Communicable diseases”)) OR (TITLE-ABS-KEY: (communicable disease)) OR (INDEXTERMS: (“Communicable Diseases, Emerging”)) OR (INDEXTERMS: ([Cysticercosis](https://www.ncbi.nlm.nih.gov/mesh/68003551))) OR (TITLE-ABS-KEY: (cysticerc*)) OR (TITLE-ABS-KEY: (“Crimean-congo haemorrhagic fever)) OR (INDEXTERMS: (Dengue)) OR (TITLE-ABS-KEY: (dengue)) OR (INDEXTERMS: (“[Hemorrhagic Fever, Ebola](https://www.ncbi.nlm.nih.gov/mesh/68019142)”)) OR (INDEXTERMS: (Ebolavirus)) OR (TITLE-ABS-KEY: (ebola)) OR (INDEXTERMS: ([Echinococcosis](https://www.ncbi.nlm.nih.gov/mesh/68004443))) OR (INDEXTERMS: (“encephalitis, viral”)) OR (TITLE-ABS-KEY: (encephalitis virus)) OR (TITLE-ABS-KEY: (echinoco*)) OR (TITLE-ABS-KEY: (enteric fever)) OR (TITLE-ABS-KEY: (Ehrlichia*)) OR (TITLE-ABS-KEY: (“erythema chronicum migrans”)) OR (TITLE-ABS-KEY: (“erythema migrans”)) OR (INDEXTERMS: (filariasis)) OR (INDEXTERMS: (Elephantiasis)) OR (INDEXTERMS: (“Elephantiasis, filarial”)) OR (TITLE-ABS-KEY: (filaria*)) OR (TITLE-ABS-KEY: (“filovirus disease”)) OR (TITLE-ABS-KEY: (“Flea borne spotted fever”)) OR (TITLE-ABS-KEY: (Trematod*)) OR (TITLE-ABS-KEY: (“Francisella tularensis”)) OR (TITLE-ABS-KEY: (“Hansen disease”)) OR (TITLE-ABS-KEY: (“Hantavirus fever”)) OR (INDEXTERMS: (“Hemorrhagic Fever Virus, Crimean-Congo”)) OR (INDEXTERMS: (“Hemorrhagic fever, omsk”)) OR (TITLE-ABS-KEY: (Hemorrhagic fever, omsk)) OR (INDEXTERMS: (“Hendra virus”)) OR (TITLE-ABS-KEY: (Hendra virus)) OR (TITLE-ABS-KEY: (“Katayama fever”)) OR (TITLE-ABS-KEY: (“Lassa fever”)) OR (TITLE-ABS-KEY: (Leishman*)) OR (INDEXTERMS: (Leprosy)) OR (INDEXTERMS: (Leptospirosis)) OR (TITLE-ABS-KEY: (lepr*)) OR (TITLE-ABS-KEY: (Leptospir*)) OR (TITLE-ABS-KEY: (Lyme*)) OR (INDEXTERMS: (Malaria)) OR (TITLE-ABS-KEY: (malaria)) OR (TITLE-ABS-KEY: (Marburg*)) OR (INDEXTERMS: (“Neglected diseases”)) OR (TITLE-ABS-KEY: (nipah)) OR (TITLE-ABS-KEY: (Neuroschistosomias*)) OR (TITLE-ABS-KEY: (schistos*)) OR (TITLE-ABS-KEY: (“North asian tick typhus”)) OR (INDEXTERMS: (Onchocerciasis OR (TITLE-ABS-KEY: (Onchocerc*)) OR (TITLE-ABS-KEY: (“Oriental spotted fever”)) OR (TITLE-ABS-KEY: (“Orientia tsutsugamushi”)) OR (TITLE-ABS-KEY: (“Pappataci fever”)) OR (TITLE-ABS-KEY: (“parasitic disease*)) OR (TITLE-ABS-KEY: (parasit*)) OR (INDEXTERMS: (“Paratyphoid fever”)) OR (TITLE-ABS-KEY: (paratyph*)) OR (INDEXTERMS: (”Phlebotomus fever”)) OR (TITLE-ABS-KEY: (phlebotomus)) OR (INDEXTERMS: (“Phlebovirus”)) OR (TITLE-ABS-KEY: (Phlebovirus)) OR (INDEXTERMS: (Plague)) OR (TITLE-ABS-KEY: (Plague*)) OR (TITLE-ABS-KEY: (Plasmodium)) OR (INDEXTERMS: (“Coxiella burnetii”)) OR (TITLE-ABS-KEY: (coxiella)) OR (TITLE-ABS-KEY: (“Q fever”)) OR (INDEXTERMS: (Rabies)) OR (TITLE-ABS-KEY: (Rabie*)) OR (TITLE-ABS-KEY: (Re emerging infectious disease)) OR (TITLE-ABS-KEY: (Relapsing fever)) OR (TITLE-ABS-KEY: (Remittent fever)) OR (TITLE-ABS-KEY: (Rickett*)) OR (INDEXTERMS: (“Rickettsia infections”)) OR (INDEXTERMS: (“Rift valley fever”)) OR (TITLE-ABS-KEY: (Rift)) OR (TITLE-ABS-KEY: (“River Blindness”)) OR (TITLE-ABS-KEY: (Robles Disease*)) OR (TITLE-ABS-KEY: (Rocky mountain spotted fever)) OR (TITLE-ABS-KEY: (Salmonella typhi)) OR (TITLE-ABS-KEY: (typh*)) OR (TITLE-ABS-KEY: (“Salmonella enterica”)) OR (TITLE-ABS-KEY: (“Sandfly fever”)) OR (INDEXTERMS: (“Schistosomiasis”)) OR (TITLE-ABS-KEY: (“Sicilian virus”)) OR (TITLE-ABS-KEY: (“sleeping sickness”)) OR (TITLE-ABS-KEY: (“Spotted fever”)) OR (TITLE-ABS-KEY: (“three-day fever”)) OR (TITLE-ABS-KEY: (“Toscana virus”)) OR (INDEXTERMS: (Trachoma)) OR (TITLE-ABS-KEY: (Trachoma)) OR (TITLE-ABS-KEY: (“tick-borne infection”)) OR (TITLE-ABS-KEY: (“tick borne viral disease”)) OR (TITLE-ABS-KEY: (“tick-borne bacterial disease”)) OR (TITLE-ABS-KEY: (“tick borne parasite”)) OR (TITLE-ABS-KEY: (“Tropical disease”)) OR (TITLE-ABS-KEY: (“Tropical neglected disease”)) OR (TITLE-ABS-KEY: (Trypanosom*)) OR (INDEXTERMS: (Tuberculosis)) OR (TITLE-ABS-KEY: (tuberculosis)) OR (INDEXTERMS: (Tularemia)) OR (INDEXTERMS: (“Typhoid fever”)) OR (TITLE-ABS-KEY: (Typhoid)) OR (TITLE-ABS-KEY: (Typhus*)) OR (TITLE-ABS-KEY: (“Vector borne infection”)) OR (TITLE-ABS-KEY: (“Vector-borne pathogen”)) OR (TITLE-ABS-KEY: (vector)) OR (TITLE-ABS-KEY: (“Viral encephalitis”)) OR (TITLE-ABS-KEY: ("Virus hemorrhagic fever")) OR (TITLE-ABS-KEY: (“water borne disease”)) OR (INDEXTERMS: (“Weil disease”)) OR (TITLE-ABS-KEY: (Weil)) OR (INDEXTERMS: (“West Nile fever”)) OR (TITLE-ABS-KEY: (West Nile Virus)) OR (TITLE-ABS-KEY: (West Nile neuroinvasive)) OR (TITLE-ABS-KEY: (West Nile Meningitis)) OR (TITLE-ABS-KEY: (West Nile meningoencephalitis)) OR (TITLE-ABS-KEY: (“West Nile poliomyelitis”)) OR (INDEXTERMS: (“Yellow fever”)) OR (TITLE-ABS-KEY: (Yellow fever)) OR (INDEXTERMS: (“Yersinia pestis”)) OR (TITLE-ABS-KEY: (Yersinia)) OR (INDEXTERMS: (“Zika virus infection”)) OR (TITLE-ABS-KEY: (Zika*)) OR (TITLE-ABS-KEY: (Zoono*)) OR (TITLE-ABS-KEY: (zoonotic pathogen))) **AND** | Target VBD and other infections |
| --- | --- |
| ((INDEXTERMS: (“Diagnostic techniques and Procedures”)) OR (TITLE-ABS-KEY: (“Diagnostic techniques and Procedures”)) OR (TITLE-ABS-KEY: (Urine sample)) OR (TITLE-ABS-KEY: (Blood sample)) OR (TITLE-ABS-KEY: (Saliva sample)) OR (TITLE-ABS-KEY: (feces sample)) OR (TITLE-ABS-KEY: (sputum sample)) OR (TITLE-ABS-KEY: (swab)) OR (TITLE-ABS-KEY: (fingerprick)) OR (TITLE-ABS-KEY: (biopsy)) OR (TITLE-ABS-KEY: (biopsies)) OR (TITLE-ABS-KEY: (Serology)) OR (TITLE-ABS-KEY: (Smear)) OR (TITLE-ABS-KEY: (Strip*)) OR (TITLE-ABS-KEY: (Microscop*)) OR (TITLE-ABS-KEY: (Immunoassay)) OR (TITLE-ABS-KEY: (“Lateral flow”)) OR (TITLE-ABS-KEY: (“reagent kit”)) OR (TITLE-ABS-KEY: (Diagnos*)) OR (INDEXTERMS: (“fluids and secretions”)) OR (TITLE-ABS-KEY: (“fluids and secretions”)) OR (INDEXTERMS: (”Diagnostic services”)) OR (TITLE-ABS-KEY: (Diagnostic services))) **AND** | Target diagnostic technologies |
| ((INDEXTERMS: (”Point-of-care testing”)) OR (TITLE-ABS-KEY: (Point of care testing)) OR (TITLE-ABS-KEY: (”Health care”)) OR (INDEXTERMS: (“Evaluation studies as topic”)) OR (TITLE-ABS-KEY: (“Evaluation studies”)) OR (TITLE-ABS-KEY: (rapid*)) OR (TITLE-ABS-KEY: (quick)) OR TITLE-ABS-KEY: (adapted) OR TITLE-ABS-KEY: (“Rapid Test”) OR (TITLE-ABS-KEY: (“Quick test”)) OR (TITLE-ABS-KEY: (“Bedside test”)) OR (TITLE-ABS-KEY: (“Low Technology”)) OR (TITLE-ABS-KEY: (easy)) OR (TITLE-ABS-KEY: (Inexpensi*)) OR (TITLE-ABS-KEY: (Implementation)) OR (INDEXTERMS: (“Reproducibility of results”)) (TITLE-ABS-KEY: (reproducibility)) OR (INDEXTERMS: (“sensitivity and specificity”)) OR (TITLE-ABS-KEY: (Sensitivity)) OR (TITLE-ABS-KEY: (Specificity)) OR (TITLE-ABS-KEY: (Reliabilit*)) OR (TITLE-ABS-KEY: (Performance)) OR (TITLE-ABS-KEY: (Efficacy)) OR (TITLE-ABS-KEY: (Effectiv*)) OR (TITLE-ABS-KEY: (Efficient*)) OR (TITLE-ABS-KEY: (development)) OR (TITLE-ABS-KEY: (Evaluation)) OR (TITLE-ABS-KEY: (Accuracy)) (TITLE-ABS-KEY: (Assessment)) OR (TITLE-ABS-KEY: (Cost)) OR (TITLE-ABS-KEY: (simpl*)) OR (TITLE-ABS-KEY: (Simplicity))) **AND** | Characteristics of Target diagnostic technologies |
| ((INDEXTERMS: (”Urban population”)) OR (TITLE-ABS-KEY: (urban population)) OR (INDEXTERMS: (“Residence characteristics”)) OR (TITLE-ABS-KEY: (residence characteristics)) OR (INDEXTERMS: (“Urban health”)) OR (TITLE-ABS-KEY: (urban health)) OR (INDEXTERMS: (”Cities”)) OR (TITLE-ABS-KEY: (city)) OR (TITLE-ABS-KEY: (cities)) OR (TITLE-ABS-KEY: (Public housing)) OR (INDEXTERMS: (”Public housing”)) OR (TITLE-ABS-KEY: (urban*)) OR (TITLE-ABS-KEY: (urban area)) OR (TITLE-ABS-KEY: (urban settings)) OR (TITLE-ABS-KEY: (periurban)) OR (TITLE-ABS-KEY: (city*)) OR (TITLE-ABS-KEY: (”metropolitan area”)) OR (TITLE-ABS-KEY: (metropolitan*)) OR (TITLE-ABS-KEY: (“non rural”)) OR (TITLE-ABS-KEY: (downtown)) OR (TITLE-ABS-KEY: (midtown)) OR (TITLE-ABS-KEY: (uptown)) OR (TITLE-ABS-KEY: (district)) OR (TITLE-ABS-KEY: (slum)) OR (TITLE-ABS-KEY: (barrio)) OR (TITLE-ABS-KEY: (township)) OR (TITLE-ABS-KEY: (municipal*)) OR (TITLE-ABS-KEY: (civic)) OR (TITLE-ABS-KEY: (building*)) OR (TITLE-ABS-KEY: (suburban)) OR (TITLE-ABS-KEY: (town)) OR (TITLE-ABS-KEY: (“informal settlement”)) OR (TITLE-ABS-KEY: (neighborhood)) OR (TITLE-ABS-KEY: (neighbourhood))) | Target population |
