# Supplementary material for: A scoping review on the field validation and implementation of rapid diagnostic tests for vector-borne and other infectious diseases of poverty in urban areas
Source: Infect Dis Poverty. 2018 Sep 3;7:87. doi: 10.1186/s40249-018-0474-8 (PMC6120097; doi:10.1186/s40249-018-0474-8)
Supplement: Supplementary file 3 — Table S2. Details of included studies. (DOCX 234 kb) [file 40249_2018_474_MOESM3_ESM.docx]

Table S2. Details of included papers

1

| Ref # | 16 |
| --- | --- |
| ID | Sin ID2 |
| Disease | Malaria |
| Authors | Gitonga CW, Kihara JH, Njenga SM, Awuondo K, Noor AM, Snow RW, Brooker S |
| Title | Use of rapid diagnostic tests in malaria school surveys in Kenya: does their under-performance matter for planning malaria control? |
| Year | 2012 |
| Language | English |
| Country | Kenya |
| Type | Peer reviewed paper |
| Design | Prospective |
| Intervention | OptiMal-IT, Paracheck-Pf device, and Paracheck-Pf dipstick |
| Reference test | Expert microscopy |
| Outcome | Performance |
| Results | Sensitivities= Paracheck-Pf device: 96.3% (95%CI 95.7-96.8), OptiMal: 94.9% (95%CI 93.3-96.5), Paracheck-Pf dipstick: 96.3% (95%CI 94.8-97.8)  Specificities= Paracheck-Pf device: 68.8% (95%CI 67.5-70.1), OptiMal: 77.4% (95%CI 74.4-80.5), Paracheck-Pf dipstick : 76.0% (95%CI 72.5-79.5)  Other= Paracheck-Pf device: PPV 64.2% (95%CI 62.9-65.6); NPV 96.9% (95%CI 96.4-97.4), OptiMal: PPV 71.5% (95% 68.3-74.8); NPV 96.2% (95%CI 94.9-97.6), Paracheck-Pf dipstick: PPV 16.6% (95%CI 13.5-19.6); NPV 99.8% (95%CI 99.4-100) |

2

| Ref # | 17 |
| --- | --- |
| ID | 14 |
| Disease | Malaria |
| Authors | Tarimo DS, Jani V, Killewo JZ |
| Title | Management of fever among under-fives and utility of malaria rapid diagnostic test under reduced malaria burden in Rufiji District, Southeastern Tanzania |
| Year | 2015 |
| Language | English |
| Country | Tanzania |
| Type | Peer reviewed paper |
| Design | Cross-sectional |
| Intervention | RDT |
| Reference test | Microscopy |
| Outcome | Impact and performance |
| Results | Impact = Among the 456 under-fives completing the second part of the interview, 130 (28.5%) received an antimalarial; of these, 109 (83.8%) were mRDT positive while 21 (16.2%) were mRDT negative. Among 100 under-fives microscopically positive for malaria, 10 were negative by mRDT and would therefore not receive antimalarial if there is strict adherence to mRDT results.  Sensitivity=90.0% (95%CI 82.4-95.1)  Specificity=94.3% (95%CI 91.4-96.4)  Other= PPV: 81.1% (95%CI 72.8-87.3) NPV: 97.2% (95%CI 94.7-98.6) |

3

| Ref # | 18 |
| --- | --- |
| ID | 156 |
| Disease | Malaria |
| Authors | Tobing HL, Syukur S, Purwati E, Zein R, Muzahar, Gani EH, Fachrial E |
| Title | Comparison of SD Bioline Malaria Ag-Pf/pan Test with Microscopic Examination for Detection of P.Falciparum, P.Vivax and Mixed Infection in South Nias, North Sumatera, Indonesia |
| Year | 2015 |
| Language | English |
| Country | Indonesia |
| Type | Peer reviewed paper |
| Design | Cross-sectional |
| Intervention | SD Bioline RDT |
| Reference test | Microscopy |
| Outcome | Performance |
| Results | Sensitivity= P. falciparum: 97%. P. vivax: 100%. Mixed infections: 97%  Specificity= P. falciparum: 100. P. vivax: 97%. Mixed infections: 100%  Other= P. falciparum: PPV: 100% NPV: 99%. P. vivax: PPV: 91%, NPV: 100% Mixed infections: PPV: 100%, NPV: 98% |

4

| Ref # | 19 |
| --- | --- |
| ID | 210 |
| Disease | Malaria |
| Authors | Ngasala BE |
| Title | Performance of three CareStart™ malaria rapid diagnostic tests after reduction in malaria prevalence in Bagamoyo, Tanzania |
| Year | 2014 |
| Language | English |
| Country | Tanzania |
| Type | Conference abstract |
| Design | Cannot tell |
| Intervention | 3 RDTs |
| Reference test | Microscopy |
| Outcome | Performance |
| Results | Sensitivity= CareStart™Combo RDT: 100.0% (95%CI 47.8-100) CareStart™ PLDH and HRP-2: 87.5% (95%CI 47.3-99.7)  Specificity= CareStart™Combo RDT: 98.6% (95%CI 92.6-100) CareStart™ PLDH and HRP-2: 100% (95%CI 81.5-100)  Other= CareStart™Combo RDT: PPV 83.3% (95%CI 35.9-99.6) compared to 50.0% (23.0- 77.0) for CareStart™ PLDH and 62.1% (42.3-79.3) for HRP-2 RDTs. All the three CareStart™ RDTs had high NPV >=98.5% (91.8-100) |

5

| Ref # | 20 |
| --- | --- |
| ID | 236 |
| Disease | Malaria |
| Authors | Laban NM, Kobayashi T, Sullivan D, Shiff CJ, Moss WJ |
| Title | Comparison of Pfhrp2-based RDTs and PCR in an area of declining malaria transmission in Southern Zambia |
| Year | 2014 |
| Language | English |
| Country | Zambia |
| Type | Conference abstract |
| Design | Cross-sectional |
| Intervention | HRP2-based RDTs |
| Reference test | Microscopy, pan Plasmodium species and nested PCR |
| Outcome | Performance |
| Results | Sensitivity=Thirty percent of 57 samples positive by nested PCR were also RDT positive. Of the 87, sixty one were positive by q-PCR for P. falciparum (53% of which were also RDT positive) |

6

| Ref # | 21 |
| --- | --- |
| ID | 366 |
| Disease | Malaria |
| Authors | Samadoulougou S, Kirakoya-Samadoulougou F, Sarrassat S, Tinto H, Bakiono F, Nebié I, Robert A |
| Title | Paracheck® rapid diagnostic test for detecting malaria infection in under five children: a population-based survey in Burkina Faso |
| Year | 2014 |
| Language | English |
| Country | Burkina Faso |
| Type | Peer reviewed paper |
| Design | Cross-sectional |
| Intervention | Paracheck RDT |
| Reference test | Microscopy |
| Outcome | Performance |
| Results | Sensitivity= 89.9% (95%CI 89.0-90.8)  Specificity= 50.4% (95%CI 48.3-52.6). Specificity did vary markedly by period of the year and area. The highest specificity was estimated to 71.3% (95%CI 65.6-77.0) before the rainy season and decreased to 44.3% (95%CI 41.4-47.2) and 51.9% (95%CI 48.1-55.7) during and after the rainy season, respectively. Specificity in urban areas was good but decreased drastically to 41.5% in rural areas  Other=PPV: 77.9% (95%CI 76.7-79.1) NPV: 72.1% (95%CI 69.7-74.3). The diagnosis accuracy was estimated at 77% and the Youden index at 40% |

7

| Ref # | 22 |
| --- | --- |
| ID | 1431 |
| Disease | Malaria |
| Authors | Grigg MJ, William T, Barber BE, Parameswaran U, Bird E, Piera K, et al. |
| Title | Combining parasite lactate dehydrogenase-based and histidine-rich protein 2-based rapid tests to improve specificity for diagnosis of malaria due to Plasmodium knowlesi and other Plasmodium species in Sabah, Malaysia |
| Year | 2014 |
| Language | English |
| Country | Malaysia |
| Type | Peer reviewed paper |
| Design | Prospective |
| Intervention | Combination RDTs pan-pLDH/Pf-pLDH (OptiMAL-IT) and VOM-pLDH/Pf-HRP2RDT (CareStart) |
| Reference test | PCR for *P. falciparum, P. vivax, P. ovale, P. malariae,* and *P. knowlesi* |
| Outcome | Performance |
| Results | Sensitivity= VOM-pLDH + Pf-HRP2: P. knowlesi 42% (95%CI 34-49) P. vivax 83% (95%CI 66-93); P. falciparum 97% (95%CI 90-99); P. malariae 67% (95%CI 9-99)  pan-pLDH + Pf-pLDH: P. knowlesi 32% (95%CI 25-39); P. vivax 60% (95%CI 42-75); P. falciparum 82% (95%CI 72-89); P. malariae 67% (95%CI 9-99)  Specificity= VOM-pLDH + Pf-HRP2: P. knowlesi 74% (95%CI 65-82); P. vivax 71% (95%CI 65-76); P. falciparum 99% (95%CI 97-100); P. malariae 35% (95%CI 30-41)  pan-pLDH + Pf-pLDH: P. knowlesi 21% (95%CI 15-29); P. vivax 97% (95%CI 94-99); P. falciparum 39% (95%CI 33-46); P. malariae 91% (95%CI 87-94)  Other= RDTs PPV 90% (95%CI 78-97) and NPV 47% (95%CI 40-53) |

8

| Ref # | 23 |
| --- | --- |
| ID | 1120 |
| Disease | Malaria |
| Authors | Mbonye A, Magnussen P, Lal S, Hansen K, Cundill B, Chandler C, Clarke S |
| Title | A cluster randomised trial introducing rapid diagnostic tests into the private health sector in Uganda: impact on appropriate treatment of malaria |
| Year | 2015 |
| Language | English |
| Country | Uganda |
| Type | Peer reviewed paper |
| Design | Cluster randomised trial |
| Intervention | RDT in drug shops |
| Reference test | Microscopy |
| Outcome | Impact, performance and acceptability |
| Results | Impact= 37.4% of clients with a negative research slide in the mRDT arm had received an ACT, compared with almost 100% of blood slide negative clients over-treated with ACTs in the presumptive diagnosis arm.  Sensitivity= 91.7% Specificity=63.1% PPV=66.8% NPV=90.8%  Acceptability= The majority of patients in the intervention arm (8,480/8,672; 97.8%) accepted to purchase an mRDT |

9

| Ref # | 24 |
| --- | --- |
| ID | 434 |
| Disease | Malaria |
| Authors | Ilombe G, Mavoko HM, Maketa V, Matangila J, Kalabuanga M, Lutumba P, Luz RD, Van Geertruyden JP |
| Title | Accuracy of the malaria rapid diagnostic test SD Bioline® in symptomatic children versus non symptomatic |
| Year | 2013 |
| Language | English |
| Country | Democratic Republic of Congo |
| Type | Conference abstract |
| Design | Cross-sectional |
| Intervention | SD Bioline |
| Reference test | Microscopy |
| Outcome | Performance |
|  | Sensitivity= General: 94.3% (95%CI 89.8-97.2)  Health care settings: 99.4% (95%CI 96.8–99.9)  Specificity= General: 79.6% (95%CI 75.7–82.2) Health care settings: 67.5% (95%CI 58.9–75.2)  Other= PPV General: 62.1% (95%CI 55.8–68.1) Health care settings: 78.9% (95%CI 72.7–84.2)  NPV General: 97.5% (95%CI 95.5–98.8)  Health care settings NPV: 98,8% (95%CI 94,4-99,9) |

10

| Ref # | 25 |
| --- | --- |
| ID | 2246 |
| Disease | Malaria |
| Authors | Metzger WG, Giron AM, Vivas-Martinez S, Gonzalez J, Charrasco AJ, Mordmuller BG, Magris M |
| Title | A rapid malaria appraisal in the Venezuelan Amazon |
| Year | 2009 |
| Language | English |
| Country | Venezuela |
| Type | Peer reviewed paper |
| Design | Rapid health impact assessment |
| Intervention | RDT |
| Reference test | Microscopy |
| Outcome | Performance |
| Results | The concordance of slide reading between health posts and CML was reported to be 99%. Results showed a lower concordance (Kappa Index = 0.74) and considerable differences between particular microscopists in the health posts due to different levels of refresher training. Concordance with microscopy was good, but not optimal (Kappa Index = 0.72) |

11

| Ref # | 26 |
| --- | --- |
| ID | 2497 |
| Disease | Malaria |
| Authors | Mendoza NM, García M, Cortés LJ, Vela C, Erazo R, Pérez P, Ospina OL, Burgos JD |
| Title | Evaluation of two rapid diagnostic tests, NOW ICT Malaria Pf/Pv and OptiMal for diagnosis of malaria |
| Year | 2007 |
| Language | Spanish |
| Country | Colombia |
| Type | Peer reviewed paper |
| Design | Cross-sectional |
| Intervention | NOW® ICT Malaria Pf/Pv and OptiMal® |
| Reference test | Microscopy |
| Outcome | Performance |
| Results | Sensitivity= NOW® ICT all: 98.4% (95%CI 93.9-99,9), P. f 98.2% (95%CI 89.4-99.9), P. v 100% (95%CI 51.7-100) OptiMAL® all: 95.2% (95%CI 85.8-98.8), P. f 94,.7% (95%CI 84.5-98.6), P. v 66.7% (95%CI 24.1-94) Parasitemia ranges P. f < 200: OptiMAL® 60%; NOW® ICT 80% >=200: OptiMAL® 100% NOW® ICT 100%  Specificity= NOW® ICT all: 98% (95%CI 93.9-99.5), P. f 98.1% (95%CI 94.1-99.5), P. v 100% (95%CI 97.7-100) OptiMAL® all: 99.3% (95%CI 95.8-100), P. f 99.4% (95%CI 96-100), P. v 99% (95%CI 96.1-99.8)  Other= NOW® ICT all PPV 95.4% (95%CI 86.2-98.8) NPV 99.3% (95%CI 95.8-100); Kappa: 0.955 (0.822-1.088; p<0,001); LR+: 49.53 LR-: 0.02 OptiMAL® all: PPV 98.4% (95%CI 90-99.9) NPV 98% (95%CI 93.9-99.5) Kappa: 0.954 (0.821-1.088; p<0,001); LR+: 143.81 LR-: 0.05 |

12

| Ref # | 27 |
| --- | --- |
| ID | 866 |
| Disease | Malaria |
| Authors | Klarkowski D, Sutamihardja A, Chiduo S, Sekonde E, Hamm T, Ohrt C, et al. |
| Title | Baseline assessments on the use of malaria rapid diagnostic tests (MRDT) in hospitals and dispensaries in Tanzania |
| Year | 2012 |
| Language | English |
| Country | Tanzania |
| Type | Conference abstract |
| Design | Cannot tell |
| Intervention | RDT testing procedures and performance, supply chain management, QA/QC, staff training, documentation, and storage and waste management |
| Reference test | Cannot tell |
| Outcome | Performance |
| Results | 44% (7/16) of health facilities scored ≤60% for testing performance and only one of 16 health facilities achieved 90% |

13

| Ref # | 28 |
| --- | --- |
| ID | 867 |
| Disease | Malaria |
| Authors | Kapito-Tembo A, Mathanga D, Fiore J, Seydel K, Liomba M, Bauleni A, et al. |
| Title | Rapid diagnostic test performance in the setting of differing transmission intensities: The malawi ICEMR experience |
| Year | 2012 |
| Language | English |
| Country | Malawi |
| Type | Conference abstract |
| Design | Cannot tell |
| Intervention | RDT |
| Reference test | Microscopy |
| Outcome | Performance |
| Results | Other= Overall, PPV of a RDT compared to microscopy was 76.1%. The RDT PPV was inversely related to transmission intensity. In the moderate transmission intensity regions PPV was 91.7% and 72.3%, while in the intense malaria transmission region the PPV was 66.7% |

14

| Ref # | 29 |
| --- | --- |
| ID | 945 |
| Disease | Malaria |
| Authors | Phommasone K, Adhikari B, Henriques G, Pongvongsa T, Phongmany P, von Seidlein L, White NJ, Day NPJ, Dondorp AM, Newton PN, Imwong M, Mayxay M |
| Title | Asymptomatic Plasmodium infections in 18 villages of southern Savannakhet Province, Lao PDR (Laos) |
| Year | 2016 |
| Language | English |
| Country | Laos |
| Type | Peer reviewed paper |
| Design | Cross-sectional |
| Intervention | RDT |
| Reference test | Ultra-sensitive quantitative polymerase chain reaction (uPCR) |
| Outcome | Performance |
| Results | Sensitivity= P. f 27.8% (95%CI 14.2-45.2) and P. v 2.7% (95%CI 0.7-8.2)  Specificity= P. f 99.9% (95%CI 99.1-100) and P. v 99.3% (95%CI 98.3-99.7)  Other= P. f PPV 90.9% (95%CI 57.1-99.5) NPV 96.5% (95%CI 94.8-97.6)  P. v PPV 37.5 (95%CI 10.2-74.1) NPV 86.7% (95%CI 84.1-88.9) |

15

| Ref # | 30 |
| --- | --- |
| ID | 1006 |
| Disease | Malaria |
| Authors | Alareqi LM, Mahdy MA, Lau YL, Fong MY, Abdul‑Ghani R, Ali AA, et al. |
| Title | Field evaluation of a PfHRP‑2/pLDH rapid diagnostic test and light microscopy for diagnosis and screening of falciparum malaria during the peak seasonal transmission in an endemic area in Yemen |
| Year | 2016 |
| Language | English |
| Country | Yemen |
| Type | Peer reviewed paper |
| Design | Cross-sectional |
| Intervention | RDT |
| Reference test | Microscopy or nested PCR |
| Outcome | Performance |
| Results | Sensitivity= RDT 96% (95%CI 90.9- 98.3) The RDT maintained its high sensitivity for the detection of P. f among children <10 years old, asymptomatic participants and those with history of antimalarial drug intake. 37.9% (95%CI 29.6-46.3) reduction in LM sensitivity was observed among children <10 years old, asymptomatic participants and those with history of anti-malarial drug intake. Although LM showed high sensitivity (93.5 %) for detecting symptomatic  malaria, such sensitivity dropped to 8.5% in case of asymptomatic malaria  Specificity= RDT 56% (95%CI 44.7-66.8) RDT showed low specificity which dropped to about 30% among people with history of antimalarial drug intake Microscopy 97.6% (95%CI 91.7-99.7).  LM maintained its high specificity for the detection of P. f  Other= PPV RDT 76.3% (95%CI 69.0–82.3) Microscopy 37.6% (95%CI 29.6–46.3) NPV RDT 90.4% (95%CI 78.8–96.8) Microscopy 51.3 % (95%CI 43.2–59.2) |

16

| Ref # | 31 |
| --- | --- |
| ID | 1021 |
| Disease | Malaria |
| Authors | Nankabirwa JI, Yeka A, Arinaitwe E, Kigozi R, Drakeley C, Kamya MR, et al. |
| Title | Estimating malaria parasite prevalence from community surveys in Uganda: a comparison of microscopy, rapid diagnostic tests and polymerase chain reaction |
| Year | 2015 |
| Language | English |
| Country | Uganda |
| Type | Peer reviewed paper |
| Design | Cross-sectional |
| Intervention | RDT and light microscopy |
| Reference test | PCR |
| Outcome | Performance |
| Results | Sensitivity of microscopy was higher in Nagongera (65.3%) compared to Walukuba (49.6%, p < 0.001) and Kihihi (40.9%, p < 0.001). The sensitivity of RDTs was similar across the three sites (range 77.2–82.8%)  The specificity of microscopy was over 98% at all three sites in both years of the study and did not change appreciably with age. The specificity of RDTs was lower than microscopy at all three sites (70.6% to 95.2%) |

17

| Ref # | 32 |
| --- | --- |
| ID | 1041 |
| Disease | Malaria |
| Authors | Larsen DA, Chisha Z, Winters B, Mwanza M, Kamuliwo M, Mbwili C, Hawela M, Hamainza B, Chirwa J, Craig AS, Rutagwera MR, Lungu C, Ngwenya-Kangombe T, Cheelo S, Miller JM, Bridges DJ, Winters AM |
| Title | Malaria surveillance in low-transmission areas of Zambia using reactive case detection |
| Year | 2015 |
| Language | English |
| Country | Zambia |
| Type | Peer reviewed paper |
| Design | Prospective |
| Intervention | Reactive detection of malaria cases by visits from health care workers to the household and neighboring households of a malaria positive case without history of travelling outside urban area |
| Reference test | Cannot tell |
| Outcome | Performance |
| Results | Limitations with human resources were primary reason for not RCD in eligible patients. In 2014 a total of 144 cases have RCD with 3955 tests performed of which 1.94% were positive. 71.5% of RCD did not find malaria-infected individuals |

18

| Ref # | 33 |
| --- | --- |
| ID | 1173 |
| Disease | Malaria |
| Authors | Turki H, Raeisi A, Malekzadeh K, Ghanbarnejad A, Zoghi S, Yeryan M, et al. |
| Title | Efficiency of nested-PCR in detecting asymptomatic cases toward malaria elimination program in an endemic area of Iran |
| Year | 2015 |
| Language | English |
| Country | Iran |
| Type | Peer reviewed paper |
| Design | Cross-sectional |
| Intervention | RDT |
| Reference test | Microscopy or nested PCR |
| Outcome | Performance |
| Results | The entire 200 thick and thin Giemsa stained blood smears were negative. No positive RDT result. There were 3 P. v positive samples (1.5%) by nested-PCR |

19

| Ref # | 34 |
| --- | --- |
| ID | 2787 |
| Disease | Malaria |
| Authors | Hashizume M, Kondo , Murakami T, Kodama M, Nakahara S, Lucas MES, Wakai S |
| Title | Use of rapid diagnostic tests for malaria in an emergency situation after the flood disaster in Mozambique |
| Year | 2006 |
| Language | English |
| Country | Mozambique |
| Type | Peer reviewed paper |
| Design | Cross-sectional |
| Intervention | Combination of RDT with clinical diagnosis |
| Reference test | Fluorescent microscopy using acridine orange |
| Outcome | Performance |
| Results | PPV of RDT + clinical diagnosis (fever or history of fever)= 87.6% (92/105) (95%CI 80.8-92.8) Clinical diagnosis (fever or history of fever)=74.6% (97/130) (95%CI 66.2-81.8) |

20

| Ref # | 35 |
| --- | --- |
| ID | 1787 |
| Disease | Malaria |
| Authors | Muhindo HM, Ilombe G, Meya R, Mitashi PM, KutekemeniA, Gasigwa D, et al. |
| Title | Accuracy of malaria rapid diagnosis test Optimal-IT in Kinshasa, the Democratic Republic of Congo |
| Year | 2012 |
| Language | English |
| Country | Democratic Republic of Congo |
| Type | Peer reviewed paper |
| Design | Two-stage cluster randomized survey |
| Intervention | Optimal-IT and Paracheck-Pf |
| Reference test | Microscopy |
| Outcome | Performance |
| Results | Sensitivity= all age groups: Health centre microscopy = 86.2% (95%CI 79.9-92.3). Optimal-IT = 79.7% (95%CI 72.4 - 86.8). Paracheck-Pf 87.8% (95%CI 81.9-93.6) Patients under 5 years:  Health centre microscopy= 90.9% (95%CI 80.5-100).  Optimal-IT= 87.9% (95%CI 76.1 - 99.6). Paracheck-Pf=90.9% (95%CI 80.5-101.2) Patients above 5 years: Health centre microscopy 84.4% (95%CI 76.8-92-0) Optimal-IT=76.7% (95%CI 67.7 - 85.5) Paracheck Pf=86.7% (95%CI 79.5-93.8)  Specificity= all age groups: Health centre microscopy = 49.1%% (95%CI 41.5-50.2) Optimal-IT=97% (95%CI 95.5-98.5) Paracheck-Pf 91.6% (95%CI 89.1-94)  Patients under 5 years:  Health centre microscopy= 47.6% (95%CI 37.7-7.4)  Optimal - IT= 97% (95%CI 93.7-100)  Paracheck-Pf= 94.2% (95%CI 89.6-98.7)  Patients above 5 years: Health centre microscopy 49.5% (95%CI 44.5-54.4) Optimal IT=97% (95%CI 95.2-98.6) Paracheck Pf=91% (95%CI 88.1-93.7)  Other= all age groups PPV: Health centre microscopy=29.4% (95%CI 24.6-34) Optimal-IT=86.7% (95%CI 80.4-93.0) Paracheck-Pf 72.0% (95%CI 62.7-79.2) Patients under 5 years PPV: Health centre microscopy=35.7% (95%CI 25.3-46.1) Optimal-IT= 90.6% (95%CI 80.2-100 ) Paracheck-Pf=83.3% (95%CI 70.9-95.8) Patients  above 5 years PPV: Health centre microscopy 27.4% (95%CI 22. -32.7) Optimal IT=85.2% (95%CI 77.3-93.0) Paracheck Pf=68.4% (95%CI 59.8-77.0)  All age groups NPV : Health centre microscopy=93.5% (95%CI 90.5-96.5) Optimal-IT=95.1% (95%CI 93.2-97) Paracheck-Pf 96.8% (95%CI 95.2-98.4) Patients under 5 years NPV: Health centre microscopy= 94.2% (95%CI 87.8-100)  Optimal-IT=96.2% (95%CI 92.4-100) Paracheck-Pf=97% (95%CI 93.6-100) Patients above 5 years NPV: Health centre microscopy 93.4% (95%CI 90.0-96.7) Optimal IT=94.8% (95%CI 92.7-97) Paracheck Pf=96.8% (95%CI 95.0-98.6) |

21

| Ref # | 36 |
| --- | --- |
| ID | 1917 |
| Disease | Malaria |
| Authors | Kahama-Maro J, D’Acremont V, Mtasiwa D, Genton B, Lengeler C |
| Title | Low quality of routine microscopy for malaria at different levels of the health system in Dar es Salaam |
| Year | 2011 |
| Language | English |
| Country | Tanzania |
| Type | Peer reviewed paper |
| Design | Cross-sectional |
| Intervention | Routine microscopy |
| Reference test | Expert miscrocopy and RDTs |
| Outcome | Impact and performance |
| Results | Impact= mean positivity rates (PR) of the routine microscopy (From April to December 2006) in Hospitals: 43%. Health centres: 62%. Dispensaries: 58% (range per facility: 14 to 93%) Mean positivity rates (PR) of the routine microscopy (post RDT initiation. during the same period from April 2007 to December 2007) in Hospitals: 6%. Health centres: 7%. Dispensaires: 8% (range per facility: 5 to 12%) Test positivity rates using RDTs (post-intervention period. from April 2007 to September 2008) in Hospitals: 7%. Health centres: 9%. Dispensaires: 9% (range 6 to 12%) Malaria positivity rates in febrile patients in the rainy and dry seasons in Buguruni Health Centre: Positive by RDT: 82 (13.6%)  Sensitivity= using expert microscopy with a single reading as a comparator. routine microscopy: 71.4% (95%CI 35.9-91.8) Using expert microscopy as reference for RDTs (rainy and dry seasons): 97.0%  Specificity= using expert microscopy with a single reading as a comparator. routine microscopy: 47.3% (95%CI 41.9-52.7) Using expert microscopy as reference for RDTs (rainy and dry seasons): 96.8%  Other PPV using expert microscopy with a single reading as a comparator routine microscopy: 2.8% (95%CI1.2-6.4) Using expert microscopy as reference for RDTs (rainy and dry seasons): 79.2% NPV using expert microscopy with a single reading as a comparator. routine microscopy: 98.7% (95%CI 95.5-99.6) Using expert microscopy as reference for RDTs (rainy and dry seasons): 99.6% |

22

| Ref # | 37 |
| --- | --- |
| ID | 2069 |
| Disease | Malaria |
| Authors | Hassanpour GR, Keshavarz H, Mohebali M, Zeraati H, Azizi E, Raiisi A |
| Title | Detection of malaria infection in blood transfusion: A comparative study among real-time PCR, rapid diagnostic test and microscopy |
| Year | 2011 |
| Language | English |
| Country | Iran |
| Type | Conference abstract |
| Design | Cannot tell |
| Intervention | PLDH/HRP2 |
| Reference test | Microscopy and PCR |
| Outcome | Performance |
| Results | All samples were negative by both microscopy and dipstick methods. Two positive samples collected from Bandar Abbas as an endemic area of malaria in Iran using real-time PCR |

23

| Ref # | 38 |
| --- | --- |
| ID | 3494 |
| Disease | Malaria |
| Authors | Houmsou RS, Amuta EU, Sar TT, Adagba AH |
| Title | Malarial infection among patients attending a Nigerian semi-urban based hospital and performance of HRP-2 pf Rapid diagnostic Test (RDT) in screening clinical cases of Plasmodium falciparum malaria |
| Year | 2011 |
| Language | English |
| Country | Nigeria |
| Type | Peer reviewed paper |
| Design | Prospective |
| Intervention | HRP-2 pf |
| Reference test | Microscopy |
| Outcome | Performance |
| Results | Sensitivity= 0.895 Specificity= 1 True positive: 51 (100%) True negative: 156 (93.9%) False negative: 10 (6.0%) False positive: 0 (0.0%) |

24

| Ref # | 39 |
| --- | --- |
| ID | 2181 |
| Disease | Malaria |
| Authors | Dolo A, Diallo M, Saye R, Konare A, Ouattara A, Poudiougo B, et al. |
| Title | Obstacles to laboratory diagnosis of malaria in Mali--perspectives |
| Year | 2010 |
| Language | French |
| Country | Mali |
| Type | Peer reviewed paper |
| Design | Cross-sectional |
| Intervention | ParaSight and OptiMAL |
| Reference test | Microscopy |
| Outcome | Performance |
| Results | Sensitivity= Results of the thick smear of the various CSCOMs in 1998: ASACOBA: 100%. ASACOMSI: 100%. ASACOSEK: 100% ASACOMA: 100%. ASACOBAFA: 100%. Global result: 100% OptiMAL compared thick smear: 1996-1998: 96%. 2003: 97% ParaSight compared thick smear: 1996-1998: 96%  Specificity= Results of the thick smear of the various CSCOMs in 1998: ASACOBA: 12.5%. ASACOMSI: 9.5%. ASACOSEK: Cannot tell. ASACOMA: 15.8% ASACOBAFA: 35%. Global result: 15.7% OptiMAL compared thick smear: 1996-1998: 98% 2003: 95%. ParaSight compared thick smear: 1996-1998: 84% Other= Results of the thick smear of the various CSCOMs in 1998: ASACOBA: PPV=46%; Kappa 11%; Concordance=50%. ASACOMSI: PPV=32%; Kappa 6%; Concordance=37%. ASACOSEK: PPV=57%; Concordance=0%. ASACOMA: PPV=41%; Kappa 12%; Concordance=47%. ASACOBAFA: PPV=43%; Kappa 26%; Concordance=56%. Global result: PPV=44%; Kappa 13%; Concordance=49% OptiMAL compared thick smear: 1996-1998: PPV=98%; NPV=97%; Kappa=94% 2003: PPV=97%; NPV=96%; Kappa=93%. ParaSight compared thick smear: 1996-1998: PPV=85%; NPV=95%; Kappa=79% |

25

| Ref # | 40 |
| --- | --- |
| ID | 2189 |
| Disease | Malaria |
| Authors | Andrade BB, Reis-Filho A, Barros AM, Souza-Neto SM, Nogueira LL, Fukutani KF, et al. |
| Title | Towards a precise test for malaria diagnosis in the Brazilian Amazon: comparison among field microscopy, a rapid diagnostic test, nested PCR, and a computational expert system based on artificial neural networks |
| Year | 2010 |
| Language | English |
| Country | Brazil |
| Type | Peer reviewed paper |
| Design | Cross-sectional |
| Intervention | Optimal IT and field microscopy |
| Reference test | nested PCR |
| Outcome | Performance |
| Results | Sensitivity= RDT 89.02% (95%CI 83.38 - 93.26) Microscopy: 81.50% (95%CI 74.9 - 87)  Specificity= RDT 100% (95%CI 97.36 -100) Microscopy: 100% (95%CI 97.36 -100)  Other= Predictive positive value: RDT 100% (95%CI 97.36 - 100). Microscopy: 100% (95%CI 97.42-100) Negative predictive value: RDT 87.90% (95%CI 87.90 - 100). Microscopy: 81.18% (95%CI 74.48 -86.75).  Of the 12 cases of mixed infections detected by the nested PCR, the RDT discriminated as being eight cases of P. falciparum infection and four cases of vivax malaria. |

26

| Ref # | 41 |
| --- | --- |
| ID | 379 |
| Disease | Malaria |
| Authors | Satyasi SK, Poosapati RK |
| Title | A study of evaluation of rapid diagnostic techniques of Malaria in Urban Slums of Vijayawada, Krishna District, Andhra Pradesh, India |
| Year | 2014 |
| Language | English |
| Country | India |
| Type | Peer reviewed paper |
| Design | Prospective |
| Intervention | PARAMAX-3 Pan/Pv/Pf and SD BIOLINE Malaria P.f/P.v tests |
| Reference test | Microscopy |
| Outcome | Performance |
| Results | Sensitivity pLDH (Paramax-3) compared to traditional blood films: Pv. + P.f: 88% (4400/50), Pv: 88% (95%CI 85.2-97.6), Pf 83.3% (1000/12) (95%CI 62.3-97.9) SD-Bioline test compared to traditional blood films Pv. + P.f 74% (3700/37) Pv: 74% (95%CI 85.2-97.6) Pf: 58.33% (700/12) (95%CI 62.3-97.9)  Specificity= pLDH (Paramax-3) compared to traditional blood films: Pv. + P.f: 100% (15000/150), Pv: 100% IC 95% (95%CI 96.2-100), Pf: 98.9% (18600/188) (95%CI 95.5-99.8) SD-Bioline test compared to traditional blood films Pv. + P.f: 100% (15000/150) Pv: 100% (95%CI 96.2-100) Pf: 100% (18800/188) (95%CI 95.5-99.8)  Other= PPV: Paramax-3 compared to traditional blood films: Pv. + P.f: 100% (4400/44) Pv: 100%(95%CI 93.9-100), Pf: 85.7% (1000/12) (95%CI 62.3-97.9) SD-Bioline test compared to traditional blood films: Pv. + P.f: 100% (3700/37) Pv: 100% (95%CI 93.9-100), Pf: 100% (700/12) (95%CI 62.3-97.9) NPV: Paramax-3 compared to traditional blood films: Pv. + P.f: 96% (15000/156) Pv: 96% (95%CI 95.5-99.8) Pf: 98.9% (18600/188) (95%CI 95.7-99.8) SD-Bioline test compared to traditional blood films: 83.33% (15000/180) Pv: 83.33% (95%CI 95.5-99.8) Pf: 97.4% (18800/19) (95%CI 95.7-99.8) |

27

| Ref # | 42 |
| --- | --- |
| ID | 2308 |
| Disease | Malaria |
| Authors | Sayang C, Soula G, Tahar R, Basco LK, Gazin P, Moyou-Somo R, Delmont J |
| Title | Use of a histidine-rich protein 2-based rapid diagnostic test for malaria by health personnel during routine consultation of febrile outpatients in a peripheral health facility in Yaounde, Cameroon |
| Year | 2009 |
| Language | English |
| Country | Cameroon |
| Type | Peer reviewed paper |
| Design | Prospective |
| Intervention | Complex intervention including training of nurses and use of RDT |
| Reference test | Microscopy |
| Outcome | Impact, performance and appropiateness |
| Results | Impact= use of the DiaSpot®RDT reduced antimalarial drug misuse from 61.6%to 8% in children 0-5 years of age and also reduced misuse from 55.4% to 12.8% in individuals above 6 years.  Sensitivity= 71.4%  Specificity= 82.2%  Appropiateness= In practice, cassette devices seemed easier to use because it was possible to write the name or code of the patient with a pen or pencil, and whole blood and buffer solution are deposited in the same well. For dipsticks, well-labeled individual test tubes for storage were needed to prevent confusion, leading to an increased operational time during outpatient care |

28

| Ref # | 43 |
| --- | --- |
| ID | 4265 |
| Disease | Malaria |
| Authors | Kamugisha ML, Msangeni H, Beale E, Malecela EK, Akida J, Ishengoma DR, Lemnge MM |
| Title | Paracheck Pf compared with microscopy for diagnosis of Plasmodium falciparum malaria among children in Tanga City, north-eastern Tanzania |
| Year | 2008 |
| Language | English |
| Country | Tanzania |
| Type | Peer reviewed paper |
| Design | Cross-sectional |
| Intervention | Paracheck Pf® |
| Reference test | Microscopy |
| Outcome | Performance |
| Results | Sensitivity= 93.1% IC 95% (90.4-96.2)  Specificity= 98.9% IC 95% (98.2-100.0)  Other= PPV: 93.3% IC 95% (90.4-96.2) NPV: 99.2% IC 95% (98.2-100.0) |

29

| Ref # | 44 |
| --- | --- |
| ID | 2629 |
| Disease | Malaria |
| Authors | Arcanjo ARL, de Lacerda MVG, Alecrim WD, Alecrim MdGC |
| Title | Evaluation of the Optimal-IT and ICT P.f./P.v. rapid dipstick tests for diagnosing malaria within primary healthcare in the municipality of Manaus, Amazonas |
| Year | 2007 |
| Language | Portuguese |
| Country | Brazil |
| Type | Peer reviewed paper |
| Design | Cannot tell |
| Intervention | Optimal-IT and ICT-P.f./P.v |
| Reference test | Microscopy |
| Outcome | Performance |
| Results | Kappa: Optimal-IT compared with thick blood smear = 0.67. ICT-P.f./P.v= 0.84 |

30

| Ref # | 45 |
| --- | --- |
| ID | 547 |
| Disease | Malaria |
| Authors | Kyabayinze DJ, Zongo I, Cunningham J, Gatton M, Angutoko P, Ategeka J, et al. |
| Title | HRP2 and pLDH-based rapid diagnostic tests, expert microscopy, and PCR for detection of malaria infection during pregnancy and at delivery in areas of varied transmission: A prospective cohort study in Burkina Faso and Uganda |
| Year | 2016 |
| Language | English |
| Country | Burkina Faso and Uganda |
| Type | Peer reviewed paper |
| Design | multi-center prospective study |
| Intervention | HRP2, pan-pLDH, Pf-pLDH, and microscopy |
| Reference test | PCR |
| Outcome | Performance |
| Results | Specificity= Burkina Faso antenatal visits: Microscopy: 99.8% (95%CI 99.1-99.9) HRP2/pan pLDH: 99.3% (95%CI 98.4-99.7) Pf pLDH/pan pLDH: 99.6% (95%CI 98.9-99.9) Delivery visit: Microscopy: 100.0% (95%CI 99.3-100.0) HRP2/pan pLDH: 100.0% (95%CI 99.3-100.0)Pf pLDH/pan pLDH: 100.0% CI 95% (95%CI 99.3-100.0) in Uganda antenatal visits: Microscopy: 98.4% (95%CI 96.7-99.2) HRP2/pan pLDH: 95.7% (95%CI 93.3-97.2) Pf pLDH/pan pLDH: 98.2% (95%CI 96.4-99.1) Delivery visit Microscopy: 99.5% (95%CI 96.6-99.9)  HRP2/pan pLDH: 96.2% (95%CI 92.4-98.1) Pf pLDH/pan pLDH: 9.0% (95%CI 96.2-99.8) Performance on antenatal samples in Uganda and Burkina Faso Microscopy: baseline value 97.3% HRP2/pan(pLDH): 91.1% PfpLDH/pan(pLDH): 96.0%  Other= Burfina Faso antenatal visits: Microscopy: PPV 98.0% (95%CI 92.2-99.5); NPV 84.8 (95%CI 82.3-86.8) HRP2/pan pLDH: PPV 95.9% (95%CI 90.9-98.1); NPV 88.3% (95%CI 86.1-90.2) Pf pLDH/pan pLDH: PPV 97.3% (95%CI 91.6-99.1); NPV 85.4% (95%CI 83.0-87.4) Delivery visit: Microscopy: PPV 100.0% (95%CI 66.2-100.0); NPV 96.9% (95%CI 95.0-98.1) HRP2/pan pLDH: PPV 100.0% (95%CI 66.2-100.0); NPV 96.9% (95%CI 95.0-98.1) Pf pLDH/pan pLDH: PPV 100.0% (95%CI 66.2-100.0); NPV 96.9% (95%CI 95.0-98.1) In Uganda antenatal visits: Microscopy: PPV 95.6% (95%CI 90.9-97.9); NPV 86.7% (95%CI 83.3-89.4) HRP2/pan pLDH: PPV 89.7% (95%CI 84.2-93.3); NPV 88.6% (95%CI 85.5-91.3) Pf pLDH/pan pLDH: PPV 94.2% (95%CI 87.7-97.1); NPV 83.1% (79.6-86.1) Delivery visit: Microscopy: PPV 97.9% (85.5-99.7); NPV 84.7% (95%CI 80.4-89.6) HRP2/pan pLDH: PPV 88.1% (95%CI 77.1-93.8); NPV 86.0% (95%CI 81.8-90.4) Pf pLDH/pan pLDH: PPV 94.9% (95%CI 80.2-98.7); NPV 81.7% (95%CI 77.0-86.7) NPV Performance on antenatal samples in Uganda and Burkina Faso: Microscopy: 75.6% HRP2/pan(pLDH): 80.8% Pf pLDH/pan(pLDH): 72.9% |

31

| Ref # | 46 |
| --- | --- |
| ID | 3094 |
| Disease | Malaria |
| Authors | Singh N, Valecha N, Nagpal AC, Mishra SS, Varma HS, Subbarao SK |
| Title | The hospital- and field-based performances of the OptiMAL test for malaria diagnosis and treatment monitoring in central India |
| Year | 2013 |
| Language | English |
| Country | India |
| Type | Peer reviewed paper |
| Design | Prospective |
| Intervention | Optimal |
| Reference test | Microscopy |
| Outcome | Performance |
| Results | Sensitivity= Hospital based: 100% Field based: 100%  Specificity= Hospital based: 97% Field based: 67%  Other= Hospital based PPV: 97.8%, NPV: 100%. Field based: PPV: 84.2%, NPV: 100% |

32

| Ref # | 47 |
| --- | --- |
| ID | 3100 |
| Disease | Malaria |
| Authors | Grobusch MP, Hanscheid T, Gobels K, Slevogt H, Zoller T, Rogler G, Teichmann D |
| Title | Comparison of three antigen detection tests for diagnosis and follow-up of falciparum malaria in travellers returning to Berlin, Germany. |
| Year | 2003 |
| Language | English |
| Country | Germany |
| Type | Peer reviewed paper |
| Design | Prospective |
| Intervention | ParaSight-F, ICT Malaria P. f., ICT Malaria P.f./P.v. and OptiMal |
| Reference test | Microscopy |
| Outcome | Performance |
| Results | Sensitivity= ParaSight-F 95.1% ICT Malaria P. f. 90.6% ICT Malaria P.f./P.v. 97.7% OptiMal 76.2%  Specificity= ParaSight-F 97.1% ICT Malaria P. f. 99.4% ICT Malaria P.f./P.v. 98.8% OptiMal 99.7% |

33

| Ref # | 48 |
| --- | --- |
| ID | 3136 |
| Disease | Malaria |
| Authors | Ferro BE, González IJ, de Carvajal F, Palma GI, Saravia NG |
| Title | Performance of OptiMAL in the Diagnosis of Plasmodium vivax and Plasmodium falciparum Infections in a Malaria Referral Center in Colombia |
| Year | 2002 |
| Language | English |
| Country | Colombia |
| Type | Peer reviewed paper |
| Design | Cannot tell |
| Intervention | OptiMAL |
| Reference test | Microscopy or PCR |
| Outcome | Performance |
| Results | Sensitivity= OptiMAL Compared with microscopy: P. f= 90.6% (95%CI 73.8-7.5) P. v= 98.6% (95%CI 91.3-99.9). Malaria overall=99.1% (95%CI 94.2-100) OptiMALCompared with PCR: P. f= 83.3%. P.v= 84.2%  Specificity= OptiMAL compared with microscopy: P. f=96.5 (95%CI 89.3-99.1) P.v: 97.6% (95%CI 91.0 -99.6) Malaria overall= 94.1 (95%CI 86.2-97.8)  Other= OptiMAL compared with microscopy: PPV P.f= 90.6% (95%CI 73.8 -97.5) P.v=97.2% (95%CI 89.4 - 99.5) Malaria overall=95.5 (95%CI 89.5-98.3) NPV P. f= 96.5% (95%CI 89.3-99.1) P.v: 98.8% (95%CI 92.6-99.9) Overall= 98.8% (95%CI 92.4-99.9) Overall diagnostic efficiency for malaria was 96.9%. The diagnostic efficiency for P.v was higher (98.1%) than for P. f (94.9%) |

34

| Ref # | 49 |
| --- | --- |
| ID | 3419 |
| Disease | Malaria |
| Authors | Mwanziva CE |
| Title | Defining malaria burden from morbidity and mortality records, self treatment practices and serological data in Magugu, Babati District, northern Tanzania |
| Year | 2012 |
| Language | English |
| Country | Tanzania |
| Type | Conference abstract |
| Design | Prospective |
| Intervention | RDT |
| Reference standard | Cannot tell |
| Outcome | Performance |
| Results | Sensitivity RDT: 15.6% Specificity RDT: 96.3% |

35

| Ref # | 50 |
| --- | --- |
| ID | 645 |
| Disease | Malaria |
| Authors | Osei-Yeboah J, Norgbe GK, Lokpo SY, Kinansua MK, Nettey L, Allotey EA |
| Title | Comparative performance evaluation of routine malaria diagnosis at Ho Municipal Hospital |
| Year | 2016 |
| Language | English |
| Country | Ghana |
| Type | Peer reviewed paper |
| Design | Cross-sectional |
| Intervention | RDTs |
| Reference test | Microscopy |
| Outcome | Performance |
| Results | Sensitivity= composite as a standard: RDT: 4.78% Presumptive diagnosis: 43.48% Field microscopy: 4.55% Microscopy as a standard: RDT: 62.50% Presumptive diagnosis: 70.83% Age stratified: ≤5 years RDT: 85.71% Presumptive diagnosis: 71.43% 6-18 years RDT: 30% Presumptive diagnosis: 7.41% 19-40 years RDT: 37.5% Presumptive diagnosis: 75% 41-85 years RDT: 50% Presumptive diagnosis: 66.67%  Specificity= composite as a standard: RDT: 72.22% Presumptive diagnosis: 23.55% Field microscopy: 82.28% Microscopy as a standard: RDT: 92.73% Presumptive diagnosis: 25.82% Age stratified: ≤5 years RDT: 86.89% Presumptive diagnosis: 26.23% 6-18 years RDT: 100% Presumptive diagnosis: 92.31% 19-40 years RDT: 96.88% Presumptive diagnosis: 30.21% 41-85 years RDT: 97.18% Presumptive diagnosis: 22.95%  Other= composite as a standard: RDT: PPV 28.57%; NPV 24.62% Presumptive diagnosis: PPV 4.52%; NPV 83.33% Field microscopy: PPV 41.67%; NPV 23.64% Microscopy as a standard: RDT: PPV 42.86%; NPV 96.59% Presumptive diagnosis: PPV 7.69%; NPV 91.03% Age stratified: ≤5 years RDT: PPV 42.86%; NPV 98.15% Presumptive diagnosis: PPV 10.00%; NPV 88.89% 6-18 years RDT: PPV 100%; NPV 85.11% Presumptive diagnosis: PPV 66.67%; NPV 32.43% 19-40 years RDT: PPV 50.00%; NPV 94.90% Presumptive diagnosis: PPV 8.22%; NPV 93.55% 41-85 years RDT: PPV 60.00%; NPV 95.83% Presumptive diagnosis: PPV 7.84%; NPV 87.50% Performance criteria with field microscopy: Total population RDT: AUC ROC 0.697; p value <0.0001; kappa 0.457; youden 0.395 Presumptive diagnosis: AUC ROC 0.506; p value 0.7304; kappa -0.007; youden 0.013 Male participants RDT: AUC ROC 0.702; p value 0.0035; kappa 0.492; youden 0.404 Presumptive diagnosis: AUC ROC 0.504; p value 0.8891; kappa 0.005; youden 0.009 Female participants RDT: AUC ROC 0.695; p value 0.0005; kappa 0.436; youden 0.390 Presumptive diagnosis: AUC ROC 0.512; p value 0.6156; kappa -0.013; youden 0.023 |

36

| Ref # | 51 |
| --- | --- |
| ID | 3518 |
| Disease | Malaria |
| Authors | Sutherland LJ, Bustinduy AL, Mungai PL, Muchiri EM, Kitron U, Zimmerman PA, King CH |
| Title | Performance of a rapid diagnostic card test for detection of single- or multi-species Plasmodium infections among residents of Southern Coast Province, Kenya |
| Year | 2011 |
| Language | English |
| Country | Kenya |
| Type | Conference abstract |
| Design | Cross-sectional |
| Intervention | RDT |
| Reference test | PCR |
| Outcome | Performance |
|  | Sensitivity= as a screening tool, ICT cards (designed for detection of P. falciparum) had sensitivity of 43% for falciparum, 10% for vivax and 0% for ovale and malariae.  Specificity= 99% for all four species. Specifcity remained the same for P.f in single vs. multi-species infections (99%), but sensitivity lowered to 29% when non-falciparum species were present.  Other= ICT PPV for P. f was 86% and NPV was 90%. For single-species P. malariae infection, the PPV was 0% and NPV was 92%. PPV for isolated P. v was 15% and NPV was 98%, and PPV for P. ovale was 0% and NPV was 97%. |

37

| Ref # | 52 |
| --- | --- |
| ID | 3621 |
| Disease | Malaria |
| Authors | Diallo A, Dos Santos S, Diop A, Barbosa L, Le Hesran JY |
| Title | Urban malaria in Dakar: Rapid diagnostic test implementation and presumptive diagnosis |
| Year | 2011 |
| Language | English |
| Country | Senegal |
| Type | Conference abstract |
| Design | Cannot tell |
| Intervention | RDT |
| Reference test | Cannot tell |
| Outcome | Performance |
| Results | The rate of positive RDT was 39% |

38

| Ref # | 53 |
| --- | --- |
| ID | 3907 |
| Disease | Malaria |
| Authors | Ntoumi F, Vouvoungui JC, Ibara R, Landry M, Sidibé A |
| Title | Malaria burden and case management in the Republic of Congo: Limited use and application of rapid diagnostic tests results |
| Year | 2013 |
| Language | English |
| Country | Congo |
| Type | Peer reviewed paper |
| Design | Prospective |
| Intervention | Mal card™ |
| Reference test | Microscopy or PCR |
| Outcome | Performance |
| Results | Microscopy and RDT quality control CSI intedance: k=0.88, CSI NdakaSossou: k 0.35 |

39

| Ref # | 54 |
| --- | --- |
| ID | 3928 |
| Disease | Malaria |
| Authors | Lee PW, Ji DD, Liu CT, Rampao HS, do Rosario VE, Lin IF, et al |
| Title | Application of loop-mediated isothermal amplification for malaria diagnosis during a follow-up study in São Tomé |
| Year | 2012 |
| Language | English |
| Country | the Republic of São Tomé and Príncipe |
| Type | Peer reviewed paper |
| Design | Prospective |
| Intervention | LAMP |
| Reference test | RDT, microscopy or PCR |
| Outcome | Performance |
| Results | Sensitivity= Nested PCR is used as a gold standard RDT: 84% (95%CI 75.3-90.6) LAMP: 100% (95%CI 96.4, 100)  Specificity= Nested PCR is used as a gold standard: RDT: 78% (95%CI 68.6-85.7) LAMP: 98% (95%CI 93.0-99.8)  Nested PCR as gold standard: PPV: RDT: 79% (95%CI 69.7-86.5), LAMP: 98% (95%CI 93.0-99.8). NPV: RDT: 83% (95%CI 74.2-89.8), LAMP: 100% (95%CI 96.4- 100). For the therapeutic follow-up, HRP-2-RDTs had very poor PPV (95%CI 18-21%) Time to obtain test results varied depending on the different methods, i.e. 20 min for HRP-2-RDTs, 60 min for microscopy, 3 hr for LAMP, and 8 hr for nested PCR |

40

| Ref # | 55 |
| --- | --- |
| ID | 4033 |
| Disease | Malaria |
| Authors | Binesh Lal Y, Jayakumar S, Kalyani M, Mathew R, Shameem Banu AS, Dhinesh R |
| Title | Correlation of quantitative Buffy Coat, blood smear and antigen detection in diagnosing malarial infection |
| Year | 2011 |
| Language | English |
| Country | India |
| Type | Peer reviewed paper |
| Design | Prospective |
| Intervention | Antigen detection and quantitative buffy coat |
| Reference test | Microscopy |
| Outcome | Performance |
| Results | Sensitivity ADT = 97.1% QBC = 80.75%  Specificity ADT = 95.42% QBC = 94.53%  Other PPV ADT = 74.44% QBC = 70%. NPV ADT = 99.58% QBC = 96.88% |

41

| Ref # | 56 |
| --- | --- |
| ID | 4071 |
| Disease | Malaria |
| Authors | Metzger WG, Vivas-Martínez S, Giron A, Vaccari E, Campos E, Rodríguez I, et al. |
| Title | Assessment of routine malaria diagnosis in the Venezuelan Amazon |
| Year | 2011 |
| Language | English |
| Country | Venezuela |
| Type | Peer reviewed paper |
| Design | Cannot tell |
| Intervention | OptiMAL-IT |
| Reference test | Microscopy |
| Outcome | Performance |
| Results | Sensitivity= Health post microscopy: Pf 67.6 (95%CI 57.9-75.9) Pv 86.1% (95%CI 83.0-88.7) All 86.1% (95%CI 83.5-88.3) Optimal-IT: Pf 88.9% (95%CI 64.6-96.9) Pv 63.4% (95%CI 57.1-67.2) All 67.0% (95%CI 61.2-70.4)  Specificity= Health post microscopy: Pf 96.0 (95%CI 95.1-96.8) Pv 91.5% (95%CI 90.0-93.5) All 88.4% (95%CI 87.2-91.3)  Optimal-IT: Pf 99.8% (95%CI 99.4-99.9) Pv 98.6% (95%CI 97.4-99.3) All: 98.6% (95%CI 97.3-99.3)  Other= Health post microscopy: Pf PPV 60.0% (95%CI 51.2-67.1) NPV 97.2% (95%CI 96.3-97.9); K=0.60 (95%CI 0.50-0.69); Pv PPV 85.2% (95%CI 82.2-87.8) NPV 92.1% (95%CI 90.3-93.5) K=0.78 (95%CI 0.73-0.82); All PPV 87.2% (95%CI 84.6-89.4) NPV 88.5% (95%CI 86.3-90.3) K 0.76 (95%CI 0.71-0.80) Optimal-IT: Pf PPV 88.9% (95%CI 64.6-96.9) NPV 99.8% (95%CI 99.4-99.9) K=0.89 (95%CI 0.64-0.97); Pv PPV 89.7% (95%CI 80.7-95.0) NPV 93.4% (95%CI 92.3-94.1) K=0.70 (95%CI 0.62-0.76); All PPV 91.0% (95%CI 83.1-95.7) NPV 93.3% (95%CI 92.1-94.4) K=0.73 (95%CI 0.65-0.78) |

42

| Ref # | 57 |
| --- | --- |
| ID | 4117 |
| Disease | Malaria |
| Authors | Sousa-Figueiredo JC, Oguttu D, Adriko M, Besigye F, Nankasi A, Arinaitwe M, et al. |
| Title | Investigating portable fluorescent microscopy (CyScope) as an alternative rapid diagnostic test for malaria in children and women of child-bearing age |
| Year | 2010 |
| Language | English |
| Country | Uganda |
| Type | Peer reviewed paper |
| Design | Cross-sectional |
| Intervention | CyScope® and Paracheck-Pf® |
| Reference test | Microscopy |
| Outcome | Performance |
| Results | Sensitivity= Paracheck-Pf outperformed CyScope with sensitivity 91.9% in adults and 98.4% in children Specificity=98.1% in adults and 96.2% in children |

43

| Ref # | 58 |
| --- | --- |
| ID | 4193 |
| Disease | Malaria |
| Authors | A-Elgayoum SME, El-Feki AEKA, Mahgoub BA, El-Rayah EA, Giha HA |
| Title | Malaria overdiagnosis and burden of malaria misdiagnosis in the suburbs of central Sudan: special emphasis on artemisinin-based combination therapy era |
| Year | 2009 |
| Language | English |
| Country | Sudan |
| Type | Peer reviewed paper |
| Design | Prospective |
| Intervention | Microscopy and RDT |
| Reference test | Expert microscopy |
| Outcome | Performance and cost |
| Results | Sensitivity= Compared with expert microscopy: Microscopy=86%, RDT= 100%  Specificity= Compared with expert microscopy: Microscopy= 29%, RDT=100%  Cost= the use of RDT instead of microscopy in the presence of well-trained GPs will raise the total malaria cost from US$14 million to US$18.8 million; however, deployment of RDT without GPs training will raise the malaria cost enormously (to US$135.8 million). However, the RDT use, regardless of GPs training, will decrease the health burden in terms of accurate diagnosis of malaria. |

44

| Ref # | 59 |
| --- | --- |
| ID | 4197 |
| Disease | Malaria |
| Authors | Chanda P, Castillo-Riquelme M, Masiye F |
| Title | Cost-effectiveness analysis of the available strategies for Diagnosing malaria in outpatient clinics in Zambia |
| Year | 2009 |
| Language | English |
| Country | Zambia |
| Type | Peer reviewed paper |
| Design | A cost-effectiveness evaluation |
| Intervention | Microscopy and RDT |
| Reference test | Expert microscopy |
| Outcome | Impact, performance and cost |
| Results | Impact= the potential savings on treatment if only cases found positive are treated were zero were56% for microscopy and 59% for RDT strategy respectively. It was found that almost 87% of all facility visits were prescribed antimalarials regardless of the malaria test result.  Accuracy= Clinical 24% Microscopy 79% RDT 91%  Cost per visit: Clinical USD 2.7, Microscopy USD 8.2 and RDT USD 4.7. Average Cost-effectivenes Ratio (Total cost/case correctly diagnosed) Clinical USD 17.1, Microscopy USD 11.9, RDT USD 6.5 |

45

| Ref # | 60 |
| --- | --- |
| ID | 4231 |
| Disease | Malaria |
| Authors | Harvey SA, Jennings L, Chinyama M, Masaninga F, Mulholland K, Bell DR |
| Title | Improving community health worker use of malaria rapid diagnostic tests in Zambia: package instructions, job aid and job aid-plus-training |
| Year | 2008 |
| Language | English |
| Country | Zambia |
| Type | Peer reviewed paper |
| Design | Prospective |
| Intervention | Job aid and training programme for use of RDT |
| Reference | List of 16 steps divided into three sub-categories: (1) preparation and documentation, (2) RDT use and (3) safe handling and disposal |
| Outcome | Performance and cost |
| Results | On average, CHWs using the manufacturer's instructions performed 57% of test steps correctly. Those using the job aid alone improved significantly to 80%. Job aid-plus-training CHWs scored highest at 90% correct.  Performance also improved with each successive RDT prepared by a CHW (data not reported). Pair-wise total scores from the second practice test were significantly higher than those from the first (mean difference: 7%, 95%CI 3–11); and scores from the third test (the one observed and reported on here) were significantly higher than those from the second (mean difference: 4%, 95%CI: 2–6). Differences between consecutive tests were larger in the manufacturer's instructions and job aid-only groups than in the job aid-plus training group.  Cost= Altogether, these materials totalled about US $66.00 per CHW trained. Including salaries, per diem, and transportation costs for the trainer, observers, and MOH personnel raises the total per CHW cost to slightly less than US $175.00. |

46

| Ref # | 61 |
| --- | --- |
| ID | 4351 |
| Disease | Malaria |
| Authors | Mboera LEG, Fanello CI, Malima RC, Talbert A, Fogliati P, Bobbio F, et al. |
| Title | Comparison of the Paracheck-Pf H test with microscopy, for the confirmation of Plasmodium falciparum malaria in Tanzania |
| Year | 2006 |
| Language | English |
| Country | Tanzania |
| Type | Peer reviewed paper |
| Design | Cannot tell |
| Intervention | Paracheck-Pf |
| Reference test | Microscopy |
| Outcome | Performance |
| Results | Sensitivity=90.0% Specificity=96.6% PPV 88.9% NPV 97.0% PLR 27.0 (95%CI 20.0–36.0) The Kappa test showed substantial interobserver agreement for the combined data from all five districts (k=50.86) |

47

| Ref # | 62 |
| --- | --- |
| ID | 4361 |
| Disease | Malaria |
| Authors | Singh N, Saxena A, Awadhia SB, Shrivastava R, Singh MP |
| Title | Evaluation of a rapid diagnostic test for assessing the burden of malaria at delivery in India |
| Year | 2005 |
| Language | English |
| Country | India |
| Type | Peer reviewed paper |
| Design | Cross-sectional |
| Intervention | Paracheck Pf and ParaHITf with placental blood |
| Reference test | Microscopy |
| Outcome | Performance |
| Results | Sensitivity= compared with microscopy Paracheck placental blood: 93.3 (95%CI 76.5–98.8), ParaHITf with placental blood: 87.5 (95%CI 75.3–94.4)  Specificity= compared with microscopy: Paracheck with placental blood: 84.4% (95%CI 78.0–89.2) ParaHITf with placental blood: 97.0% (95%CI 95.1–98.2)  Other= compared with microscopy PPV: Paracheck with placental blood: 50.0% (95%CI 36.5–63.5) ParaHITf with placental blood:75.4% (95%CI 62.9–84.9) NPV: Paracheck with placental blood: 98.7% (95%CI 94.9–99.8), ParaHITf with placental blood: 98.7% (95%CI 97.2–99.4) |

48

| Ref # | 63 |
| --- | --- |
| ID | 4590 |
| Disease | Malaria |
| Authors | Harutyunyan V |
| Title | Quality assurance of malaria rapid diagnostic tests (RDT) and its implication for clinical management of malaria |
| Year | 2010 |
| Language | English |
| Country | Tanzania |
| Type | Conference abstract |
| Design | Cannot tell |
| Intervention | RDT at the clinics |
| Reference test | Microscopy |
| Outcome | Impact and performance |
| Results | Impact= after sharing these results with the clinicians, malaria diagnoses based on laboratory confirmation in the clinics rose within two months from 38% of total malaria diagnoses to 64%.  Sensitivity= 93.7% Specificity= 92.2% |

49

| Ref # | 64 |
| --- | --- |
| ID | 4602 |
| Disease | Malaria |
| Authors | Phiri M, Kobayashi T, Chishimba S, Stresman G, Mharakurwa S, Thuma P, et al |
| Title | The predictive value of rapid diagnostic tests for gametocytemia identified by RT-PCR |
| Year | 2010 |
| Language | English |
| Country | Zambia |
| Type | Conference abstract |
| Design | Cross-sectional |
| Intervention | ICT Malaria P. f cassette (pfHRP-2) and RT-PCR |
| Reference test | RT-PCR for gametocytes |
| Outcome | Performance |
| Results | RDT: 31 (10%) positive for gametocytes RT-PCR: 14 (4.5%) positive for gametocytes RDT + RT-PCR: 9 (2.9%) Almost half (45%) of the RDT positive individuals also tested positive for gametocytes. Of the gametocyte positive individuals, 64% were RDT positive |

50

| Ref # | 65 |
| --- | --- |
| ID | 4612 |
| Disease | Malaria |
| Authors | Viana GM, Chamma NN, Barbosa DR, do Carmo EL, Nascimento JM, Peres JM, et al. |
| Title | Evaluation of two rapid diagnostic tests for malaria (OptiMAL-IT® and pALUTOP+4®) in an endemic area of Para State, Brazilian Amazon Region |
| Year | 2010 |
| Language | English |
| Country | Brazil |
| Type | Peer reviewed paper |
| Design | Cannot tell |
| Intervention | PALUTOP+, OptiMAL-IT |
| Reference test | Microscopy or nested PCR |
| Outcome | Performance |
| Results | Sensitivity= Optimal compared with microscopy: 73.9%. PALUTOP+4 compared with microscopy: 85.2%  Specificity= Optimal compared with microscopy: 100%. PALUTOP+4 compared with microscopy: 53.8%  Accuracy= Optimal compared with microscopy: 83.2%. PALUTOP+4 compared with microscopy: 72.3% Kappa for OptiMAL-lT® was 66.7% when compared to microscopy and 62.4% when compared to nested-PCR (substantial agreement); the same index for PALUTOP+4® was 40.5% (moderate agreement) and 32.0% (fair agreement) compared to microscopy and nested-PCR, respectively |

51

| Ref # | 66 |
| --- | --- |
| ID | 6361 |
| Disease | Malaria |
| Authors | Gerstl S, Dunkley S, Mukhtar A, De Smet M, Baker S, Maikere J |
| Title | Assessment of two malaria rapid diagnostic tests, with followup of positive pLDH test results, in a hyperendemic falciparum malaria area |
| Year | 2009 |
| Language | English |
| Country | Sierra Leone |
| Type | Conference abstract |
| Design | Cannot tell |
| Intervention | CareStart™ and Paracheck-Pf |
| Reference test | Blood slide |
| Outcome | Performance |
|  | Sensitivity= Paracheck-Pf: 98.8% (95%CI 95.8-99.8, 2/169) CareStart™, 99.4% (95%CI 96.8-100.0, 1/169)  Specificity= Paracheck-Pf 74.7% (95%CI 67.6-81.0, 44/174) CareStart™ 96.0% (95%CI 91.9- 98.4, 7/174)  Other= of the 155 eligible study subjects for follow-up CareStart™ test, 63.9% (99/155) had a positive test on day 2, 21.3% (33/155) on day 7, 5.8% (9/155) on day 14, 1.9% (3/155) on day 21 and 0.6% (1/155) on day 28. |

52

| Ref # | 67 |
| --- | --- |
| ID | 6739 |
| Disease | Malaria |
| Authors | Abdul W, Rathor HR, Khail AAK, Abdullah MA, Amin U, Ahmad A |
| Title | Incidence of malaria and comparison of microscopy and rapid diagnostic test in District Dir Lower, Lower Dir, Khyber Pakhtunkhwa, Pakistan |
| Year | 2016 |
| Language | English |
| Country | Pakistan |
| Type | Peer reviewed paper |
| Design | Cannot tell |
| Intervention | RDT |
| Reference test | Microscopy |
| Outcome | Performance |
| Results | Sensitivity= 99% Specificity=100% |

53

| Ref # | 68 |
| --- | --- |
| ID | 933 |
| Disease | Malaria |
| Authors | Mahende C, Ngasala B, Lusingu J, Yong T-S, Lushino P, Lemnge M, et al. |
| Title | Performance of rapid diagnostic test, blood-film microscopy and PCR for the diagnosis of malaria infection among febrile children from Korogwe District, Tanzania |
| Year | 2016 |
| Language | English |
| Country | Tanzania |
| Type | Peer reviewed paper |
| Design | Cannot tell |
| Intervention | RDT |
| Reference test | Microscopy or PCR |
| Outcome | Performance |
| Results | Sensitivity= HRP2-based malaria RDT agains microscopy: 88.9% (95%CI 79.3-95.1) HRP2-based malaria RDT and microscopy against PCR: RDT: 88.6% (95%CI 79.5-94.7) Microscopy: 91.1% (95%CI 82.6-96.4)  Specificity= HRP2-based malaria RDT against microscopy: 97.4% (95%CI 96.0-98.4)HRP2-based malaria RDT and microscopy against PCR: RDT: 97.8% (95%CI 96.3.98.8) Microscopy: 100.0% (95%CI 99.4-100.0)  Other= HRP2-based malaria RDT agains microscopy: PPV: 75.3% (95%CI 64.8-84.0) NPV: 99.0% (95%CI 98.0-99.6) HRP2-based malaria RDT and microscopy against PCR PPV RDT: 84.3% (95%CI 74.7-91.4) Microscopy: 100.0% (95%CI 95.0-100.0) NPV RDT: 98.5% (95%CI 97.1-99.3) Microscopy: 98.8% (95%CI 97.6-99.5) |

54

| Ref # | 69 |
| --- | --- |
| ID | 6836 |
| Disease | Malaria |
| Authors | Zahid U, Badshah N, Nadeem MF, Azam H, Khattak AA |
| Title | Evaluation of immunochromatographic (ICT) assay and microscopy for malaria diagnosis in endemic district Dera |
| Year | 2015 |
| Language | English |
| Country | Pakistan |
| Type | Peer reviewed paper |
| Design | Cannot tell |
| Intervention | RDT |
| Reference test | Microscopy |
| Outcome | Performance |
| Results | Sensitivity=100%  Specificity= 99.28%  Other= PPV of 98.98% and NPV of 100% |

55

| Ref # | 70 |
| --- | --- |
| ID | 6964 |
| Disease | Malaria |
| Authors | Bansal R, Jindal N, Sidhu S |
| Title | Disease dynamics and surveillance of malaria in Malwa region of Punjab and evaluation of RDT test |
| Year | 2013 |
| Language | English |
| Country | India |
| Type | Peer reviewed paper |
| Design | Retrospective |
| Intervention | OptiMAL |
| Reference test | Microscopy |
| Outcome | Performance |
| Results | Sensitivity= OptiMAL compared with microscopy: 97.4%  Specificity= OptiMAL compared with microscopy: 100%  Other=PPV OptiMAL compared with microscopy: 100% NPV: OptiMAL compared with microscopy: 99.8% |

56

| Ref # | 71 |
| --- | --- |
| ID | 7079 |
| Disease | Malaria |
| Authors | Panchal HK, Desai PB |
| Title | ICT - a rapid, innovative but simple technique for malaria diagnosis |
| Year | 2012 |
| Language | English |
| Country | India |
| Type | Peer reviewed paper |
| Design | Cannot tell |
| Intervention | ICT ParaHIT Total |
| Reference test | Field stained blood smears |
| Outcome | Performance |
| Results | Sensitivity=ICT: 92.12%  Specificity= ICT: 98.41% |

57

| Ref # | 72 |
| --- | --- |
| ID | 961 |
| Disease | Malaria |
| Authors | Cook J, Grignard L, Al-Eryani S, Al-Selwei M, Mnzava A, Al-Yarie H, et al. |
| Title | High heterogeneity of malaria transmission and a large sub-patent and diverse reservoir of infection in Wusab As Safil district, Republic of Yemen |
| Year | 2016 |
| Language | English |
| Country | Yemen |
| Type | Peer reviewed paper |
| Design | Cannot tell |
| Intervention | RDT and microscopy, molecular a serological analyses |
| Reference test | Microscopy or PCR or serological tests ELISA to detect IgG against *P.falciparum*  and *P. vivax* MSP-1 and *P. vivax* AMA-1 |
| Outcome | Performance |
| Results | Sensitivity= RDT 45.8% (95%CI 41.0-50.6) Microscopy 41.6% (95%CI 36.9-46.4)  Specificity= RDT 95.4% (95%CI 94.3-96.3) Microscopy 97.1% (95%CI 96.3-97.9)  Other= kappa 0.83 (RDT and microscopy) RDT and microscopy did not detect 45 % of infections present The mean multiplicity of infection (MOI) was 2.3 and high heterozygosity and allelic richness were detected |

58

| Ref # | 73 |
| --- | --- |
| ID | 7231 |
| Disease | Malaria |
| Authors | Kamugisha E, Mazigo HD, Manyama MM, Rambau PP, Mirambo MM, Kataraihya JB, et al. |
| Title | Low sensitivity but high specificity of ParaHIT-f in diagnosing malaria among children attending outpatient department in Butimba District Hospital, Mwanza, Tanzania |
| Year | 2009 |
| Language | English |
| Country | Tanzania |
| Type | Peer reviewed paper |
| Design | Cross-sectional |
| Intervention | ParaHIT-f |
| Reference test | Microscopy |
| Outcome | Performance |
| Results | Sensitivity= 29.80% Specificity= 98.80% PPV: 82.4% NPV: 88.3% |

59

| Ref # | 74 |
| --- | --- |
| ID | 7396 |
| Disease | Malaria |
| Authors | Valecha N, Eapen A, Usha Devi C, Ravindran J, Aggarwal A, Subbarao SK. |
| Title | Field evaluation of the ICT Malaria P.f./P.v. immunochromatographic test in India |
| Year | 2002 |
| Language | English |
| Country | India |
| Type | Peer reviewed paper |
| Design | Cannot tell |
| Intervention | ICT P.f./P.v. RDT |
| Reference test | Microscopy |
| Outcome | Performance |
| Results | Sensitivity= P. f 95.9% (95%CI 94-98) P. v 70.6% (95%CI 66-75)  Specificity= P. f 93% (95%CI 91-95) P. v 99,4% (95%CI 98-100) |

60

| Ref # | 75 |
| --- | --- |
| ID | 7403 |
| Disease | Malaria |
| Authors | Mendoza NM, Montoya R, Garcia M, Padilla JC, Bruzon LO, Mendoza E, et al. |
| Title | Evaluation of a rapid diagnostic field test for malaria |
| Year | 2001 |
| Language | Spanish |
| Country | Colombia |
| Type | Peer reviewed paper |
| Design | Cross-sectional |
| Intervention | OptiMAL |
| Reference test | Bloos smear |
| Outcome | Performance and cost |
| Results | Sensitivity= OptiMAL: 98.7% (95%CI 92.5-99.9) OptiMAL Pf 98.1% (95%CI 88.4-99.9) OptiMAL Pv 90.9% (95%CI 69.4-98.4)  Specificity= OptiMAL: 99.3% (95%CI 95.9-100.0) OptiMAL Pf 76.9% (95%CI 55.9-90.2) OptiMAL Pv 100% (95%CI 92.0-100.0)  Other= OptiMAL: Kappa 0.98 PPV: 98.7% (95%CI 92.1-99.9) NPV: 99.3% (95%CI 95.9-100.0)  Cost= OptiMAL: US $2.00 (COP $4.200) Thick blood smear: US $1.80 (COP $3.800) |

61

| Ref # | 76 |
| --- | --- |
| ID | 7423 |
| Disease | Malaria |
| Authors | Singer LM, Newman RD, Diarra A, Moran AC, Huber CS, Stennies G, et al. |
| Title | Evaluation of a malaria rapid diagnostic test for assessing the burden of malaria during pregnancy |
| Year | 2004 |
| Language | English |
| Country | Burkina Faso |
| Type | Peer reviewed paper |
| Design | Cross-sectional |
| Intervention | MAKROmed to detect *P.f* HRP2 |
| Reference test | Microscopy or PCR |
| Outcome | Performance |
| Results | Sensitivity= Compared with PCR Placental RDT: 89%, Placental microscopy: 58%. Peripheral RDT: 92%, Peripheral microscopy: 67%. Peripheral RDT: 90%, Peripheral microscopy: 67%  Specificity= Compared with PCR Placental RDT: 76%, Placental microscopy: 96%. Peripheral RDT: 59%, Peripheral microscopy: 88% Peripheral RDT: 65%, Peripheral microscopy: 98% |

62

| Ref # | 77 |
| --- | --- |
| ID | 986 |
| Disease | Malaria |
| Authors | Sumari D, Grimberg BT, Blankenship D, Mugasa J, Mugittu K, Moore L, Gwakisa P, Zborowski M |
| Title | Application of magnetic cytosmear for the estimation of *Plasmodium falciparum* gametocyte density and detection of asexual stages in asymptomatic children |
| Year | 2016 |
| Language | English |
| Country | Tanzania |
| Type | Peer reviewed paper |
| Design | Cross-sectional |
| Intervention | Magnetic Deposition Microscopy |
| Reference test | ICT dual malaria cassette or light microscopy |
| Outcome | Performance |
| Results | Sensitivity= LM 36.5 % (95%CI 32.2–60.5) and RDT 62.5 % (95%CI 49.5–71.8) compared to MDM  Specificity= the MDM specificities with respect to LM and RDT to all forms of P. falciparum were 87.5 % (95%CI 71.2–89.6 %) and 89.0% (95%CI 82.9–91.4), respectively  Other= The prevalence of P. f by MDM, RDT and LM were 19.5 % (95%CI 15.2–24.4), 17.2% (95%CI 13.1–21.9) and 7.6% (95%CI 4.9–11.2), respectively. The prevalence of P. f gametocytes was 1.3% (4/303 95%CI 0.4–3.3) by LM but was considerably higher and equal to 4% (12/303 95%CI 2.1–6.8) by the MDM |

63

| Ref # | 78 |
| --- | --- |
| ID | 7334 |
| Disease | Malaria |
| Authors | Rajendran C, Dube SN |
| Title | Field evaluation of a rapid immunochromatographic test kit for the diagnosis of Plasmodium falciparum and non-falcipraum malaria parasites from Sonitpur district, Assam. |
| Year | 2006 |
| Language | English |
| Country | India |
| Type | Peer reviewed paper |
| Design | Cannot tell |
| Intervention | ICT Parascreen test |
| Reference test | Microscopy |
| Outcome | Performance |
| Results | Sensitivity= RDT for P.f: 96.3%. RDT for non-P.f: 88.88%  Specificity= RDT for P.f: 98.48%. RDT for non-P.f: 98.48% |

64

| Ref # | 79 |
| --- | --- |
| ID | 16 |
| Disease | Malaria |
| Authors | Ahmed SM, Tefera M |
| Title | Malaria diagnosis and treatment practice following introduction of rapid diagnostic test in selected health posts of Adama Woreda, East Shewa Zone, Oromia Region, Centeral Ethiopia |
| Year | 2015 |
| Language | English |
| Country | Ethiopia |
| Type | Conference abstract |
| Design | Cross-sectional |
| Intervention | RDT |
| Outcome | Impact |
| Results | Impact= 79.8% patients were seen in health posts with available RDT, Overall malaria positivity rate was 57.8%, anti-malaria drugs were prescribed to 100% patients with positive RDT and 54.3% of RDT negative patients. |

65

| Ref # | 80 |
| --- | --- |
| ID | 2149 |
| Disease | Malaria |
| Authors | Kyabayinze DJ, Asiimwe C, Nakanjako D, Nabakooza J, Counihan H, Tibenderana JK |
| Title | Use of RDTs to improve malaria diagnosis and fever case management at primary health care facilities in Uganda |
| Year | 2010 |
| Language | English |
| Country | Uganda |
| Type | Peer reviewed paper |
| Design | quasi-experimental |
| Intervention | RDTs |
| Outcome | Impact, adoption and appropiateness |
| Results | Impact= 38% point reduction in anti-malarial prescriptions in all study health facilities when RDTs were introduced to support malaria clinical diagnosis. Use of RDTs resulted in a 2-fold decrease (RR=0.52 95%CI 0.51-0.54) in antimalarial drug prescription of (59% drop) in the hypo-endemic district of Kapchorwa.  Adoption= 92% of the respondents health care workers believed that positive RDT results were always truly positive and 51% of the respondents health care workers believed that negative RDT results were always truly negative. Utilization of test kits: 90% (35,154) of the patients with suspected malaria were investigated with RDTs, 30% of the patients found to have negative RDT results were prescribed AMD, 1% of positive patients were not prescribed AMD, 99% with positive RDT results received AMD prescription irrespective of age.  Appropiateness= 98% health care workers said they communicate RDTs results to patients. 44% of the health workers mentioned that they do not always have to treat RDT positive patients and the possible reason for not treating may be due to the current guidelines that recommend referral of severe malaria cases.  40% mentioned that they would consider treating based on clinical suspicion despite a negative RDT test result. 98% of the health workers said they were willing and committed to performing RDTs on a daily basis in the management of out-patients who present with fever. |

66

| Ref # | 81 |
| --- | --- |
| ID | 588 |
| Disease | Malaria |
| Authors | Zongo S, Farquet V, Ridde V |
| Title | A qualitative study of health professionals’ uptake and perceptions of malaria rapid diagnostic tests in Burkina Faso |
| Year | 2016 |
| Language | English |
| Country | Burkina Faso |
| Type | Peer reviewed paper |
| Design | Qualitative |
| Intervention | Complex intervention introducing RDTs for the diagnosis of malaria |
| Outcome | Impact, acceptability and appropiateness |
| Results | Impact= RDTs had not changed their prescribing practices.  Acceptability= In the different health centers of the two health districts, the health professionals’ attitudes toward RDTs were definitely heterogeneous, and the logic underlying their use varied. Nevertheless, there was convergence around the need to use this innovation in patient care interactions. RDTs were also seen by the clinicians as adding to their workload and prolonging the consultation time.  Appropiateness= The health professionals mentioned some drawbacks associated with the tool’s use. The lack of gloves for performing the test was mentioned in both districts |

67

| Ref # | 82 |
| --- | --- |
| ID | 725 |
| Disease | Malaria |
| Authors | Alumbasi LT, Gagova I, Mikolasova G, Sokolova J, Kulkova N, Silharova B, et al. |
| Title | Decreased occurrence of highland malaria after introduction of point-of-care rapid diagnostic tests in Kenyan highland near Eldoret in 2250 meters above sea level |
| Year | 2013 |
| Language | English |
| Country | Kenya |
| Type | Conference abstract |
| Design | Cross-sectional |
| Intervention | RDTs |
| Outcome | Impact |
| Results | When RDTs were introduced in January 2011, in combination with blood smear microscopy in our laboratory, the number of confirmed cases dropped to 1919 in 2011 and 748 in first six months of 2012, which is 2.5 to 3-times (80%) less than in previous years. |

68

| Ref # | 83 |
| --- | --- |
| ID | 1264 |
| Disease | Malaria |
| Authors | Diggle E, Asgary R, Gore-Langton G, Nahashon E, Mungai J, Harrison R, et al. |
| Title | Perceptions of malaria and acceptance of rapid diagnostic tests and related treatment practises among community members and health care providers in Greater Garissa, North Eastern Province, Kenya |
| Year | 2014 |
| Language | English |
| Country | Kenya |
| Type | Peer reviewed paper |
| Design | Qualitative |
| Intervention | Official introduction of RDTs in Health facilities |
| Outcome | Impact, acceptability and sustainability |
| Results | Impact= the total number of AL doses dispensed in 2013 was at a four-year low of 4,519 treatments, down from the peak of 56,511 treatments dispensed in 2011  Acceptability= for patients the resemblance of RDT to HIV testing kits poses a problem, as some patients fear they are being tested for HIV, or they believe it is not the right test. The RDT was noted for its ease and swiftness of use, portability and non-reliance on electricity. They also noted a level of scepticism towards RDTs, both among patients and clinic staff, due to their surprising high rate of negative results, as compared with what both patients and providers were used to seeing when only symptoms were used for diagnosis  Sustainability= CHWs reported RDTs not being consistently available in the quantities needed. Participants recounted that it is common to expect a positive diagnosis, and to not accept or trust a negative RDT result. Local pharmacy shops make it easy to obtain malaria treatment, and make it available by request (without use of RDT or microscopy). |

69

| Ref # | 84 |
| --- | --- |
| ID | 1557 |
| Disease | Malaria |
| Authors | Mubi M, Kakoko D, Ngasala B, Premji Z, Peterson S, Björkman A, et al. |
| Title | Malaria diagnosis and treatment practices following introduction of rapid diagnostic tests in Kibaha District, Coast Region, Tanzania |
| Year | 2013 |
| Language | English |
| Country | Tanzania |
| Type | Peer reviewed paper |
| Design | Cross-sectional |
| Intervention | Malaria diagnostic and treatment according the TDR implementation |
| Outcome | Impact, acceptability, adoption, sustainability |
| Results | Impact= Antimalarials were prescribed to all 31 positive patients and 14% of negative patients. 28% non tested patients were given antimalarials. Adherence to diagnostics was 90.5%  Acceptability= health workers main advantage of RDT is ease of use (70%), shorter time to results (55%) no need of electricity (30%) other are: helps to target treatment, confirm/rule out malaria, accurate/specific, and patient satisfaction. Disadvantages are fase negative/inaccurate results 70%, lack of trust by patients, test remaining positive after treatment, tests not able to quantify parasites, negative results in patients with severe symptoms and invalid results (control band non-reactive). 72% health workers have confidence in RDT results because they are approved by relevant authorities, they are sensitive/specific, consistent with symptoms and matches transmission season and patients respond to drugs. The reasons for not confidence were: false negative results, negative results in patients with symptoms, test remaining positive after treatment and good response to drugs in patients with negative results. 39% health workers said patients did not trust RDT because patients assume fever is due to malaria, positive results in private facilities and suspecting they were tested for HIV  Adoption= the overall parasite-base testing rate was 63% (105/168): 66% in facilities equipped with RDT. There were more positive cases in the RDT tested group than in the microscopy group (38 vs 21%), and parasite-based testing was more common in children under 5yo  Sustainability= nine of ten health facilities reported RDT stock-outs at some point since their introduction |

70

| Ref # | 85 |
| --- | --- |
| ID | 2019 |
| Disease | Malaria |
| Authors | D’Acremont V, Kahama-Maro J, Swai , Mtasiwa D, Genton B, Lengeler C |
| Title | Reduction of anti-malarial consumption after rapid diagnostic tests implementation in Dar es Salaam: a before-after and cluster randomized controlled study |
| Year | 2011 |
| Language | English |
| Country | Tanzania |
| Type | Peer reviewed paper |
| Design | Before-after and cluster randomized controlled study. |
| Intervention | Complex intervention including sensitization, training of the clinical and laboratory staff and implementation of mRDT |
| Outcome | Impact |
| Results | The percentage of reduction of prescribed anti-malarials: 22% in intervention versus 60% in control HF (from a baseline in both of 79%) (RR 0.30, 95%CI 0.14-0.70) The proportion of patients that were prescribed antibiotics was higher in intervention HF than in controls: 71% versus 53% respectively (RR = 1.34, 95%CI 1.08-1.70) - from a baseline of 50 and 51%.  The proportion of patients tested for malaria increased in all health facilities except 2, from a median of 30% to 42% (PP ratio 1.21) when mRDTs were introduced and thereafter the proportion of patients tested was stable up to the end of the project |

71

| Ref # | 86 |
| --- | --- |
| ID | 3694 |
| Disease | Malaria |
| Authors | Silumbe K, Yukich JO, Hamainza B, Bennett A, Earle D, Kamuliwo M, et al. |
| Title | Costs and cost-effectiveness of a large-scale mass testing and treatment intervention for malaria in Southern Province, Zambia |
| Year | 2015 |
| Language | English |
| Country | Zambia |
| Type | Peer reviewed paper |
| Design | Cluster-randomized trial |
| Intervention | mass testing and treatment (MTAT) with RDTs and artemether-lumefantrine |
| Outcome | Impact and cost |
| Results | Impact= the three MTAT rounds were estimated to have prevented over 16,000 malaria cases and more than 30 deaths, resulting in a net gain of more than 1,300 DALYs in the year following the intervention. This translates to a cost-effectiveness estimate of USD 894 per DALY averted.  Cost= the overall cost per test administered was USD 4.39, while the overall cost per treatment administered was USD 34.74. RDTs themselves were estimated to cost USD 0.47 per kit and ACT was estimated to cost USD 1 for a child and USD 1.25 for an adult, based on project record data. |

72

| Ref # | 87 |
| --- | --- |
| ID | 3730 |
| Disease | Malaria |
| Authors | Hutchinson E, Chandler C, Clarke S, Lal S, Magnussen P, Kayendeke M, et al. |
| Title | ‘It puts life in us and we feel big’: shifts in the local health care system during the introduction of rapid diagnostic tests for malaria into drug shops in Uganda |
| Year | 2015 |
| Language | English |
| Country | Uganda |
| Type | Peer reviewed paper |
| Design | Qualitative |
| Intervention | RDTs in registered drug shops |
| Outcome | Impact |
| Results | In both arms of the project, involvement in it brought immediate economic benefits: drug shop vendors reported an increase in consultations (which may have arisen due to availability of ACTs at affordable prices and/or increased customer confidence in DSVs), as well as other benefits from improved cash flow: selling RDTs and/or Coartem that had been given free of charge by the project (‘You have helped me to have my income increase because now I can get money that I get from that coartem we get at no cost and then be able to buy other drugs’) |

73

| Ref # | 88 |
| --- | --- |
| ID | 3759 |
| Disease | Malaria |
| Authors | Hasselback L, Crawford J, Chaluco T, Rajagopal S, Prosser W, Watson N |
| Title | Rapid diagnostic test supply chain and consumption study in Cabo Delgado, Mozambique: Estimating stock shortages and identifying drivers of stock-outs |
| Year | 2014 |
| Language | English |
| Country | Mozambique |
| Type | Peer reviewed paper |
| Design | Prospective |
| Intervention | Evaluation of drivers of RDT stock shortages |
| Outcome | Impact and adoption |
| Results | Impact= lost consumption percentages of malaria RDTs by health centre Catapua: 116% Namanhumir: 32% Mecojo: 70% Nakoto: 149% Paquite: 40% Meloco: 44% Nguida: 0% Namacande: 12% Muxara: 117% Pundanhar: 32% Miteda: 20% Cagembe: 133% Namatil: 100%  Adoption= Proportion of health centres reporting stock-outs of malaria RDTs  Overall: laboratory form 17%; pharmacy form 17%; stock card 59% October: laboratory form 33% (95%CI 8-58); pharmacy form 9% (95%CI 0-25); stock card 27% (95%CI 2-52) November: laboratory form 15% (95%CI 0-33); pharmacy form 10% (95%CI 0-28); stock card 45% (95%CI 17-73) December: laboratory form 46% (95%CI 20-72); pharmacy form 13% (95%CI 0-36); stock card 64% (95%CI 37-91) January: laboratory form 43% (95%CI 19-67); pharmacy form 33% (95%CI 3-63); stock card 91% (95%CI 75-107)  February: laboratory form 0%; pharmacy form 0%; stock card 64% (95%CI 37-91) March: laboratory form 0%; pharmacy form 25% (95%CI 0-54); stock card 55% (95%CI 27-83) April: laboratory form 0%; pharmacy form 22% (95%CI 0-48); stock card 73% (95%CI 48-98) May: laboratory form 0%; pharmacy form 22% (95%CI 0-48); stock card 55% (95%CI 27-83) |

74

| Ref # | 89 |
| --- | --- |
| ID | 4294 |
| Disease | Malaria |
| Authors | Moonasar D, Goga AE, Frean J, Kruger P, Chandramohan D |
| Title | An exploratory study of factors that affect the performance and usage of rapid diagnostic tests for malaria in the Limpopo Province, South Africa |
| Year | 2007 |
| Language | English |
| Country | South Africa |
| Type | Peer reviewed paper |
| Design | Qualitative |
| Intervention | RDT |
| Outcome | Impact and acceptability |
| Results | Impact= three (15%) nurses reported that they gave antimalarial drugs to RDT negative patients if the clinical presentation was suggestive of malaria.  Acceptability= nursing staff had huge praise for the use of the RDTs |

75

| Ref # | 90 |
| --- | --- |
| ID | 4588 |
| Disease | Malaria |
| Authors | Tusiime RN, Wabwire-Mangen F, Najjemba R |
| Title | Knowledge and compliance of health workers to malaria rapid diagnostic test guidelines in Rukungiri District, Western Uganda, 2010 |
| Year | 2010 |
| Language | English |
| Country | Uganda |
| Type | Conference abstract |
| Design | Cross-sectional |
| Intervention | RDT |
| Outcome | Impact |
| Results | Of all 460 patients seen at health facilities, two-thirds (69%) were managed in compliance with the national RDT guidelines |

76

| Ref # | 91 |
| --- | --- |
| ID | 428 |
| Disease | Malaria |
| Authors | Mbonye A, Magnussen P, Lal S, Hansen K, Cundill B, Chandler C, Clarke S |
| Title | A cluster randomised trial introducing rapid diagnostic tests into the private health sector in Uganda: impact on appropriate treatment of malaria |
| Year | 2013 |
| Language | English |
| Country | Uganda |
| Type | Conference abstract |
| Design | Cluster randomised trial |
| Intervention | RDT in drug shops compared to presumptive treatment of malaria |
| Outcome | Impact and acceptability |
| Results | The intervention reduced sales of artemisinine combination therapies (ACTs) by approximately 40% compared to drug shops in the control arm (presumptive diagnosis). Drug shops using RDTs were more likely to refer patients and referral was mainly reported for RDT-negative clients. Introduction of RDTs in drug shops was acceptable to drug shop vendors (DSVs), the community and health staff |

77

| Ref # | 92 |
| --- | --- |
| ID | 2042 |
| Disease | Malaria |
| Authors | Chandler CIR, Hall-Clifford R, Asaph T, Magnussen P, Clarke S, Mbonye AK |
| Title | Introducing malaria rapid diagnostic tests at registered drug shops in Uganda: Limitations of diagnostic testing in the reality of diagnosis |
| Year | 2011 |
| Language | English |
| Country | Uganda |
| Type | Peer reviewed paper |
| Design | Qualitative |
| Intervention | RDT |
| Outcome | Acceptability |
| Results | Tests were particularly appealing as a means to confirm an expected malaria diagnosis. However, malaria tests were distrusted when they clashed with the patient’s or provider’s expectation for a negative result. When asked what would happen if the RDT result was negative, most DSWs initially responded that they would refer the client to a health facility. However, if the patient had the signs and symptoms of malaria, most went on to argue that they would still give antimalarial treatment. |

78

| Ref # | 93 |
| --- | --- |
| ID | 2069 |
| Disease | Malaria |
| Authors | Mbonye AK, Ndyomugyenyi R, Turinde A, Magnussen P, Clarke S, Chandler C |
| Title | The feasibility of introducing rapid diagnostic tests for malaria in drug shops in Uganda |
| Year | 2010 |
| Language | English |
| Country | Uganda |
| Type | Peer reviewed paper |
| Design | Qualitative |
| Intervention | RDT |
| Outcome | Acceptability and feasibility |
| Results | Acceptability= most community members and drug sellers did not know about RDTs for malaria diagnosis; but after they received an explanation, they felt it would be a useful intervention. Health officials also saw the advantage in convenience and more accurate treatment. The presence of RDTs was seen as beneficial to all drug shop attendants interviewed, who saw that the tests would improve their reputation and attract patients. RDTs were perceived to improve access to effective treatment of malaria, reduce costs associated with poor treatment, and irrational drug use. However, it perceived a risk of introducing RDTs due to increased cost of the test, potentially forcing clients to seek care elsewhere, a potential delay to referral and a lack of integration with the public health system which would not support supervision and evaluation of RDT use.  Feasibility= our study suggests that introducing rapid diagnostic tests for malaria into drug shops is feasible. Community members and drug sellers perceived RDTs as a useful strategy for effective treatment of malaria. However, we identify a number of challenges that will need to be addressed if RDTs are to be used effectively, including guidelines for treatment or referral of RDT negative patients, It will also be necessary for communities to be sensitized about the test, regulation or subsidization of the price of RDTs and a system for training and supervising drug sellers to use RDTs and adhere to results |

79

| Ref # | 94 |
| --- | --- |
| ID | 3498 |
| Disease | Malaria |
| Authors | Brieger WR, Orji BC, Otolorin E, Ndekhedehe E, Nwadike JU |
| Title | Establishing integrated community management of malaria, pneumonia and diarrhea in selected two Local Government Areas, Akwa Ibom State Nigeria |
| Year | 2011 |
| Language | English |
| Country | Nigeria |
| Type | Conference abstract |
| Design | Cannot tell |
| Intervention | Cannot tell |
| Outcome | Acceptability and feasibility |
| Results | Acceptability= Providers' and community poor acceptance of RDTs. There is a belief that 'blood of someone alive cannot be buried', to address such challenges we held community dialogue and agreed that used cassettes will be sent for appropriately burning before burial. This was more acceptable to the community members. Providers' and volunteers request for incentives and motivation as program are seen as a burden.  Feasibility= difficulty in sourcing RDTs that come with a complete set of ready to use components |

80

| Ref # | 95 |
| --- | --- |
| ID | 4119 |
| Disease | Malaria |
| Authors | Uzochukwu BSC, Chiegboka LO, Enwereuzo C, Nwosu U, Okorafor D, Onwujekwe OE, et al. |
| Title | Examining appropriate diagnosis and treatment of malaria: availability and use of rapid diagnostic tests and artemisinin-based combination therapy in public and private health facilities in south east Nigeria |
| Year | 2010 |
| Language | English |
| Country | Nigeria |
| Type | Peer reviewed paper |
| Design | Cross-sectional |
| Intervention | RDT |
| Outcome | Acceptability and adoption |
| Results | Acceptability= out of the 23 respondents that had used RDTs more than 90% of the respondents rated RDTs to be either good, very good or excellent.  Adoption= 31.1% of healthcare providers ever used RDT. The most common method for diagnosing malaria in the study area by health workers was syndromic approach, followed by microscopy and then RDT examination |

81

| Ref # | 96 |
| --- | --- |
| ID | 6759 |
| Disease | Malaria |
| Authors | Boadu NY, Amuasi J, Ansong D, Einsiedel E, Menon D, Yanow SK |
| Title | Challenges with implementing malaria rapid diagnostic tests at primary care facilities in a Ghanaian district: a qualitative study |
| Year | 2016 |
| Language | English |
| Country | Ghana |
| Type | Peer reviewed paper |
| Design | Qualitative |
| Intervention | RDT |
| Outcome | Acceptability, appropiateness and feasibility |
| Results | Acceptability= providers, concerned with compromised test quality due to poor storage, had little confidence in the veracity of test results. Infrequent quality assurance and control visits to facilities by authorities further undermined providers’ willingness to use RDTs. RDTs were considered appropriate for basic facilities in remote settings without laboratory infrastructure. Providers at hospitals and larger health centres with laboratories clearly stated their preference for microscopy.  Appropiateness= heavy caseloads resulted in long laboratory wait times that precluded test results informing clinical decision-making in real-time  Feasibility= that RDT supplies from the district health directorate to their facilities were often insufficient and sporadic. |

82

| Ref # | 97 |
| --- | --- |
| ID | 515 |
| Disease | Malaria |
| Authors | Hansen KS, Pedrazzoli D, Mbonye A, Clarke S, Cundill B, Magnussen P, et al. |
| Title | Willingness-to-pay for a rapid malaria diagnostic test and Artemisinin-based combination therapy from private drug shops in Mukono district, Uganda |
| Year | 2012 |
| Language | English |
| Country | Uganda |
| Type | Peer reviewed paper |
| Design | A contingent valuation survey with a willingness-to-pay (WTP) format |
| Intervention | RDT |
| Outcome | Acceptability |
| Results | The geometric mean WTP for an RDT was UGX1067 (US$0.53) The majority of respondents (95%) reported that it would be useful if a test for malaria could be performed at drug shops. |

83

| Ref # | 98 |
| --- | --- |
| ID | 1176 |
| Disease | Malaria |
| Authors | Silumbe K, Chiyende E, Finn TP, Desmond M, Puta C, Hamainza B, et al. |
| Title | A qualitative study of perceptions of a mass test and treat campaign in Southern Zambia and potential barriers to effectiveness |
| Year | 2015 |
| Language | English |
| Country | Zambia |
| Type | Peer reviewed paper |
| Design | Qualitative |
| Intervention | Mass malaria testing and treatment (MTAT) with artemether-lumefantrine (AL)(Coartem®) |
| Outcome | Acceptability and appropiateness |
| Results | Acceptability= focus group discussions with the MTAT community members indicated that in general they were pleased with the MTAT programme. CHWs involved in conducting test and treat activities understood the rationale for the MTAT intervention. Participants in the community FGDs acknowledged that some members of the community did not readily participate in the MTAT activities. The primary reported reasons for refusing to be tested included suspicion that CHWs could be practicing satanism and may use their blood for rituals, fear of collected blood being sold or used to test for HIV infection, other uncertainties about how the collected blood would be used, and anxiety about the entire process of testing and treating.  Appropiateness= the most common themes related to challenges encountered during implementation of MTAT included inadequate transport, the need to cover long distances, problems with PDAs, and inadequate compensation and supplies |

84

| Ref # | 99 |
| --- | --- |
| ID | 1191 |
| Disease | Malaria |
| Authors | Ranasinghe S, Ansumana R, Lamin JM, Bockarie AS, Bangura U, Buanie JAG, et al. |
| Title | Attitudes toward home-based malaria testing in rural and urban Sierra Leone |
| Year | 2015 |
| Language | English |
| Country | Sierra Leone |
| Type | Peer reviewed paper |
| Design | Cross-sectional |
| Intervention | Malaria testing |
| Outcome | Acceptability, adoption and appropiateness |
| Results | Acceptability = there were participants who consistently expressed preferences for home or laboratory based testing of whom, 71.2% (42/59) of home-based testing and 78.8% (52/66) of laboratory based preference usually consults with a healthcare provider about suspected malaria. 62.7% (37/59) of home based and 80.0% (52/65) of laboratory based had a healthcare provider recommended a diagnostic test during the most recent consultation for a fever (p= 0.033). 47.5% (28/59) of home based and 63.6% (42/66) of laboratory based had a diagnostic test to determine the cause of most recent febrile illness (p-value= 0.069). 39.7% (23/58) of home based and 77.8% (49/63) of laboratory based usually takes a diagnostic test to confirm suspected malaria (p-value= <0.001). 44.1% (26/59) of home based and 20.9% (14/67) of laboratory based were willing to pay for a home-based test (p-value= 0.005). 45.8% (27/59) of home based and 16.4% (11/67) of laboratory preference were very confident to have the ability to perform a malaria test on self without assistance (p-value= <0.001) compared to somewhat confident 45.8% (27/59) for home based and 26.9% (18/67) Laboratory-based and, Not at all confident in 8.5% (5/59) home-based and 56.7% (38/67) for laboratory-based testing preference. Confidence in ability to perform a malaria test if trained on how to use a test kit Very confident: Home-based testing preference: 83.1% (49/59), Laboratory-based testing preference: 37.3% (25/67) (p-value= <0.001). Somewhat confident: Home-based testing p  : 13.6% (8/59), Laboratory-based testing preference: 23.9% (16/67), Not at all confident: Home-based testing preference: 3.4% (2/59); Laboratory-based testing preference: 38.8% (26/67)  Adoption=Preferences for malaria test location Home-based test by a trained CHV: 40.9% (63/154); Laboratory-based test: 58,4% (90/154); No preference: 0,6% (1/154); Would not take the test either way: 0,0% (0/154) When asked if would you prefer to take a malaria test at a clinical laboratory or in your home? Home-based test by self/family: 52.6% (81/154); Laboratory-based test: 46.1% (71/154); No preference: 1.3% (2/154); Would not take the test either way: 0.0% (0/154) If test done at home, would you prefer to do the test yourself (or have a family member help you) or have a community health volunteer assist you? Home-based test by self/family: 21.3% (33/155); Home-based test by a trained CHV: 76.8% (119/155); No preference: 0.6% (1/155); Would not take the test either way: 1.3% (2/155)  Appropiateness =Reasons for not seeking diagnostic testing for the last febrile illness Did not want to spend money on the test Home-based (by self/family or CHV) testing preference: 71.0% (22/31); Laboratory-based testing preference: 82.6% (19/23). Wanted to wait a few days to see if the fever went away  Home-based (by self/family or CHV) testing preference: 63.3% (19/30); Laboratory-based testing preference: 52.4% (11/21). Did not want to travel to a laboratory Home-based (by self/family or CHV) testing preference: 54.8% (17/31); Laboratory-based testing preference: 60.9% (14/23) Did not need a test because already knew the cause of the fever Home-based (by self/family or CHV) testing preference: 83.9% (26/31); Laboratory-based testing preference: 52.4% (11/21). A doctor or other healthcare provider did not recommend a test  Home-based (by self/family or CHV) testing preference: 25.8% (8/31); Laboratory-based testing preference: 31.6% (6/19). Did not want to have to wait for the test results Home-based (by self/family or CHV) testing preference: 35.5% (11/31); Laboratory-based testing preference: 56.5% (13/23). Does not like blood draws Home-based (by self/family or CHV) testing preference: 16.1% (5/31); Laboratory-based testing preference: 34.8% (8/23) |

85

| Ref # | 100 |
| --- | --- |
| ID | 1735 |
| Disease | Malaria |
| Authors | Ezeoke OP, Ezumah NN, Chandler CCI, Mangham-Jefferies LJ, Onwujekwe OE, Wiseman V, et al. |
| Title | Exploring health providers’ and community perceptions and experiences with malaria tests in South-East Nigeria: a critical step towards appropriate treatment |
| Year | 2012 |
| Language | English |
| Country | Nigeria |
| Type | Peer reviewed paper |
| Design | Qualitative |
| Intervention | Malaria tests |
| Outcome | Acceptability and adoption |
| Results | Acceptability= community and health providers perceptions that test are not always accurate  Adoption= amongst the reasons given by health providers on why people do not demand tests was their perceived self-recognition of malaria symptoms. Health providers recognised testing for malaria was important because symptoms may overlap with those of other illnesses. However, not everybody with fever will be referred for a test. For example depending of the seriousness of the symptoms and willingness of the patient to accept the test.  Financial constraints deter people from having malaria test, the level of awareness also play a role. Lack of facilities for testing. |

86

| Ref # | 101 |
| --- | --- |
| ID | 853 |
| Disease | Malaria |
| Authors | Boadu NYA, Ansong D, Amuasi JH, Nguah SB, Arhin B, Somuah S, Yanow SK |
| Title | A review of malaria rapid diagnostic tests (RDT) guideline implementation in a district hospital in Ghana: has rapid testing been prioritized? |
| Year | 2012 |
| Language | English |
| Country | Ghana |
| Type | Conference abstract |
| Design | Cannot tell |
| Intervention | RDT |
| Outcome | Adoption |
| Results | RDT use ranged from 1.6% to about 4%, from low to high malaria incidence months. Testing with microscopy almost doubling from 10% in non-peak, to 19.3% in peak malaria incidence months |

87

| Ref # | 102 |
| --- | --- |
| ID | 1753 |
| Disease | Malaria |
| Authors | Masaninga F, Sekeseke-Chinyama M, Malambo T, Moonga H, Babaniyi O, Counihan H, et al. |
| Title | Finding parasites and finding challenges: improved diagnostic access and trends in reported malaria and anti-malarial drug use in Livingstone district, Zambia |
| Year | 2012 |
| Language | English |
| Country | Zambia |
| Type | Peer reviewed paper |
| Design | Retrospective |
| Intervention | RDT |
| Outcome | Adoption |
| Results | Rate of issue of RDTs from district level to health centres from start of 2007 to late 2009 and recorded malaria rate for the corresponding period 1st QTR-2007: RDTs issued 0; recorded malaria 12186. 2nd QTR-2007: RDTs issued 575; recorded malaria 7806. 3rd QTR-2007: RDTs issued 850; recorded malaria 10731. 4th QTR-2007: RDTs issued 2300; recorded malaria 3295. 1st QTR-2008: RDTs issued 2400; recorded malaria 3196. 2nd QTR-2008: RDTs issued 3350; recorded malaria 1081. 3rd QTR-2008: RDTs issued 3850; recorded malaria 68. 4th QTR-2008: RDTs issued 5700; recorded malaria 26; 'Unconfirmed' malaria 177. 1st QTR-2009: RDTs issued 4275; recorded malaria 8; 'Unconfirmed' malaria 276. 2nd QTR-2009: RDTs issued 4675; recorded malaria 13; 'Unconfirmed' malaria 345. 3rd QTR-2009: RDTs issued 2875; recorded malaria 2; 'Unconfirmed' malaria 378 |

88

| Ref # | 103 |
| --- | --- |
| ID | 3460 |
| Disease | Malaria |
| Authors | Ospina OL, Cortés LJ, Cucunubá ZM, Mendoza NM, Chaparro P |
| Title | Caracterizacion de la red nacional de diagnostico de malaria, Colombia 2006-2010 |
| Year | 2012 |
| Language | Spanish |
| Country | Colombia |
| Type | Conference abstract |
| Design | Descriptive |
| Intervention | RDT |
| Outcome | Adoption |
| Results | Increased number of diagnostic facilities from 2084 in 2006 to 2975 in 2010 of which 61 are RDT facilities. Routine direct quality assessment in 58% labs and indirect assessment done in 89% labs in 2010. Average percentage of labs visited per year: 35.4% health technicians labs vs 29.5 public and private labs |

89

| Ref # | 104 |
| --- | --- |
| ID | 3761 |
| Disease | Malaria |
| Authors | Fenny AP, Hansen KS, Enemark U, Asante FA |
| Title | Quality of uncomplicated malaria case management in Ghana among insured and uninsured patients |
| Year | 2014 |
| Language | English |
| Country | Ghana |
| Type | Peer reviewed paper |
| Design | Cross-sectional |
| Intervention | RDT and microscopy |
| Outcome | Adoption |
| Results | Laboratory capacity Medical laboratory: district hospital 3; private hospital 1; public health centres 4; CHPS 0 Staff trained to perform microscopy: district hospital 3; private hospital 1; public health centres 4; CHPS 0 Functional microscope, according to the laboratory technician: district hospital 3; private hospital 1; public health centres 4; CHPS 0 Staff trained to perform RDTs: district hospital 3; private hospital 1; public health centres 7; CHPS 1 Malaria testing by microscopy: district hospital 3; private hospital 1; public health centres 4; CHPS 0 Malaria testing by both microscopy and RDT: district hospital 2; private hospital 1; public health centres 2; CHPS 1 |

90

| Ref # | 105 |
| --- | --- |
| ID | 7074 |
| Disease | Malaria |
| Authors | Lima J, Abeyasinghe RR, Fitzpatrick R, Fernando SD |
| Title | Diagnosis and treatment of malaria by health care providers: findings from a post conflict district in Sri Lanka |
| Year | 2012 |
| Language | English |
| Country | Sri Lanka |
| Type | Peer reviewed paper |
| Design | Cannot tell |
| Intervention | Guidelines for diagnostics, treatment and reporting of malaria to Health Care Providers (HCPs) |
| Outcome | Adoption |
| Results | HCPs 37 (51%) were familiar with the national guidelines for the treatment of malaria issued by the AMC, 68 (94%) would refer a suspected malaria patient for confirmation of diagnosis by laboratory testing (microscopy or RDT), 8 HCPs (25% of the population) reported having clinically suspected malaria in patients presenting with fever and/or other clinical manifestations of the disease. All patients thus suspected of having malaria were referred for laboratory confirmation. Three of the HCPs (government doctors) referred the suspected malaria patients for microscopic diagnosis to the Batticaloa General Hospital and the rest based their treatment on the results made available by the laboratory technicians working in the nearest government hospital or private laboratories. |

91

| Ref # | 106 |
| --- | --- |
| ID | 3520 |
| Disease | Malaria |
| Authors | Aborigo RA, Atuguba F, Chatio S, Adoctor J, Binka F, Allen DR |
| Title | Malaria diagnosis and treatment behaviors among public and private sector health care providers in a phase IV trial in Northern Ghana |
| Year | 2011 |
| Language | English |
| Country | Ghana |
| Type | Conference abstract |
| Design | Prospective |
| Intervention | Cannot tell |
| Outcome | Adoption |
| Adoption | No results reported |

92

| Ref # | 107 |
| --- | --- |
| ID | 433 |
| Disease | Malaria |
| Authors | Hansen K, Mbonye A, Lal S, Magnussen P, Clarke S |
| Title | Incremental cost-effectiveness analysis of introducing rapid diagnostic testing for malaria into registered drug shops in Uganda |
| Year | 2013 |
| Language | English |
| Country | Uganda |
| Type | Poster presentation |
| Design | Cluster randomised trial |
| Intervention | RDTin drug shops compared to presumptive treatment of malaria |
| Outcome | Cost |
| Results | "Results will be presented" |

93

| Ref # | 108 |
| --- | --- |
| ID | 4105 |
| Disease | Malaria |
| Authors | Faye A, Ndiaye P, Diagne-Camara M, Badiane O, Wone I, Diongue M, et al. |
| Title | Economic evaluation of rapid diagnostic tests in malaria treatment |
| Year | 2010 |
| Language | French |
| Country | Senegal |
| Type | Peer reviewed paper |
| Design | Cross-sectional |
| Intervention | Economic contribution of TDR to the rational use of CTA |
| Outcome | Cost |
| Results | Cost (A) Acquisition by the State; (B) Session at the Sick  Costs of treatment without RDT: Children's kits (95 cases): (A) unit 287 CFA, total 27,265 CFA; (B) unit 300 CFA, total 28,500 CFA Adolescents' kits (48 cases): (A) unit 553 CFA, total 26,544 CFA; (B) unit 300 CFA, total 14,400 CFA Adults' kits (236 cases): (A) unit 1,043 CFA, total 246,148 CFA; (B) unit 600 CFA, total 141,600 CFA Total cost: (A) 299,957; (B) 184,500 Grant of the State: 115,457 CFA Costs of treatment with RDT: Children's kits (24 cases): (A) unit 287 CFA, total 6,888 CFA; (B) unit 300 CFA, total 7,200 CFA Adolescents' kits (33 cases): (A) unit 553 CFA, total 18,249 CFA; (B) unit 300 CFA, total 9,900 CFA Adults' kits (93 cases): (A) unit 1,043 CFA, total 96,999 CFA; (B) unit 600 CFA, total 55,800 CFA TDR (379): (A) unit 350 CFA, total 132,650 CFA; (B) unit 350 CFA, total 132,650 CFA Total cost: (A) 254,786; (B) 205,550 Grant of the State: 49,236 CFA |

94

| Ref # | 109 |
| --- | --- |
| ID | 3491 |
| Disease | Malaria |
| Authors | Ansah EK, Whitty CJ, Yeung S, Hansen K |
| Title | Cost-effectiveness analysis of introducing rapid diagnostic tests (RDTs) for malaria diagnosis in public health centers where microscopy is available and peripheral clinics where only clinical diagnosis is available: the case of Ghana |
| Year | 2011 |
| Language | English |
| Country | Ghana |
| Type | Conference abstract |
| Design | Prospective |
| Intervention | Optimal IT VS microscopy VS clinical |
| Outcome | Cost |
| Results | The proportion of correctly treated patients was similar between the RDT and the microscopy arms and that the costs per correctly treated patient were at a similar level. In the two health centres with no microscope, the proportion of correctly treated patients was higher and the costs lower in the RDT arm as compared to the clinical diagnosis arm. |

95

| Ref # | 110 |
| --- | --- |
| ID | 4128 |
| Disease | Malaria |
| Authors | Yukich J, D'Acremont V, Kahama J, Swai N, Lengeler C |
| Title | Cost Savings with rapid diagnostic tests for Malaria in low-transmission areas: Evidence from Dar es Salaam, Tanzania |
| Year | 2010 |
| Language | English |
| Country | Tanzania |
| Type | Peer reviewed paper |
| Design | Cannot tell |
| Intervention | RDT |
| Outcome | Cost |
| Results | Provider costs: The total cost of the RDT intervention over this period (not including the test kits) was estimated to be $16,946 in 2008 USD or $1,883 USD per implementing facility. Thus, we estimated that the cost of implementation per RDT (excluding the test kits themselves) was between 0.04 USD and 0.05 USD. The test kits themselves were estimated to cost USD 0.66 each. When calculating the cost per patient in RDT clinics, we include the cost of RDT implementation. Patients direct costs: Patients’ mean total expenditures were lower in RDT clinics (USD 1.02) compared with control clinics (USD 1.33), and were significantly different by the Kruskal-Wallis test for equality of populations. Patients’ mean expenditure on drugs was 0.36 USD lower in RDT clinics than in control clinics. For patients, it is likely that there is some reduced overall spending when RDTs are available. However, the savings is small (USD 0.36) and it represents only a small component of the total economic costs to patients. For providers, the drug cost savings is of a similar order (USD 0.43) as a result of RDT introduction. Unfortunately, these savings appear to be too small to offset the entire cost of RDT introduction and use. Thus, it appears that RDTs may increase the cost of treatment per patient in public facilities, despite reducing anti-malarial drug use and creating drug cost savings for the health system. |

96

| Ref # | 111 |
| --- | --- |
| ID | 4157 |
| Disease | Malaria |
| Authors | Uzochukwu BSC, Obikeze EN, Onwujekwe OE, Onoka CA, Griffiths UK |
| Title | Cost-effectiveness analysis of rapid diagnostic test, microscopy and syndromic approach in the diagnosis of malaria in Nigeria: Implications for scaling-up deployment of ACT |
| Year | 2009 |
| Language | English |
| Country | Nigeria |
| Type | Peer reviewed paper |
| Design | Prospective |
| Intervention | RDT, clinical diagnosis and microscopy |
| Outcome | Cost |
| Results | The mean total patient cost (Drugs, consultation, registration) was $2.52 (SD = $3.63) RDT cost was $0.76 and microscopy $1.30 The prevalence level (43.1%) showed an incremental cost effectiveness ratio (ICER) of $221 per death averted between RDT and syndromic treatment. For the patient cohort of 100,000, there is also a lesser cost of RDT ($0.34 million) compared to presumptive treatment ($0.37 million) and microscopy ($0.39 million) with effectiveness values of 99,862, 99,735 and 99,851 deaths averted for RDT, presumptive treatment and microscopy respectively. RDT is more cost saving than the other diagnostic strategies even when the sensitivity is measured at different levels between 5% to 15%. RDT is cost-saving at the base level of 90% RDT sensitivity with costs increasing with rise in RDT sensitivity. Reduction in sensitivity of microscopy also led to a reduction in costs of microscopic diagnosis strategy. With doubling of cost of RDT from $0.76 to $1.14, use of RDT option becomes less cost-effective than presumptive treatment at a malaria prevalence level of 40%. RDT was found to be less cost-effective at all prevalence levels above 30%. Despite doubling of costs of RDT, microscopy is found to be more costly at all malaria prevalence levels though not so when compared with presumptive treatment strategy. RDT remains more cost-effective than the other diagnostic options at a prevalence level of 40% though with an ICER value of 51,008 which is relatively very high.  At malaria prevalence level of above 40%, RDT is more cost-effective than microscopic and presumptive diagnosis, and at levels below 40%, it still dominates both strategies. With a reduction in cost of ACT by half from $2.50 to $1.25, presumptive treatment will be more cost-effective than RDT and microscopy at malaria prevalence level of 40%. At lower prevalence rates of 20% and 30%, microscopy is dominated relative to presumptive treatment with ICER values of 313 and 411 respectively. |

97

| Ref # | 112 |
| --- | --- |
| ID | 4141 |
| Disease | Malaria |
| Authors | Uzochukwu BS, Onwujekwe OE, Uguru NP, Ughasoro MD, Ezeoke OP |
| Title | Willingness to pay for rapid diagnostic tests for the diagnosis and treatment of malaria in southeast Nigeria: ex post and ex ante |
| Year | 2010 |
| Language | English |
| Country | Nigeria |
| Type | Willingness to pay survey |
| Design | Cannot tell |
| Intervention | RDT |
| Outcome | Cost |
| Results | For the ex post WTP, the willingness to pay for RDTs was positive in 89.8% of the respondents and the mean WTP was 335.1 Naira (2.58 USD). For the ex ante WTP, the willingness to pay for RDTs was positive in 38.1% of the respondents and the mean WTP was 209.27 Naira (1.61 USD) |

98

| Ref # | 113 |
| --- | --- |
| ID | 3760 |
| Disease | Malaria |
| Authors | Mbonye AK, Magnussen P, Chandler CI, Hansen KS, Lal S, Cundill B, Lynch CA, Clarke SE |
| Title | Introducing rapid diagnostic tests for malaria into drug shops in Uganda: design and implementation of a cluster randomized trial |
| Year | 2014 |
| Language | English |
| Country | Uganda |
| Type | Peer reviewed paper |
| Design | Cluster randomized trial |
| Intervention | RDT plus training and job aids vs traning and job aids |
| Outcome | Details of study design |

99

| Ref # | 114 |
| --- | --- |
| ID | 1044 |
| Disease | Malaria |
| Authors | Mbonye AK, Clarke SE, Lal S, Chandler CI, Hutchinson E, Hansen KS, Magnussen P |
| Title | Introducing rapid diagnostic tests for malaria into registered drug shops in Uganda: lessons learnt and policy implications |
| Year | 2015 |
| Language | English |
| Country | Uganda |
| Type | Peer reviewed paper |
| Design | Prospective |
| Intervention | RDT plus training and job aids Vs training and job aids provided to drug shop vendors |
| Outcome | Lessons learnt |

100

| Ref # | 115 |
| --- | --- |
| ID | 4589 |
| Disease | Malaria |
| Authors | Ishengoma D, Lwitiho S, Madebe R, Persson O, Nyagonde N, Vestergaard L, et al. |
| Title | Using Rapid Diagnostic Tests (RDTS) as source of malaria parasite DNA for molecular analyses |
| Year | 2010 |
| Language | English |
| Country | Tanzania |
| Type | Conference abstract |
| Design | Cannot tell |
| Intervention | DNA extraction using ParaHIT®f |
| Outcome | Other use |
| Results | DNA was extracted and successfully amplified by 29 positive RDTs from the field |

101

| Ref # | 116 |
| --- | --- |
| ID | 131 |
| Disease | Tuberculosis |
| Authors | Giang DC, Duong TN, Ha DTM, Nhan HT, Wolbers M, Nhu NTQ, et al. |
| Title | Prospective evaluation of GeneXpert for the diagnosis of HIV- negative pediatric TB cases |
| Year | 2015 |
| Language | English |
| Country | Vietnam |
| Type | Peer reviewed paper |
| Design | Prospective |
| Intervention | GeneXpertMTB/RIF, smear and automated liquid culture (BACTEC MGIT) |
| Reference test | Clinical standard (confirmed, probable and possible TB) and Confirmed TB. Confirmed cases were defined among those with at least 1 defined sign or symptom suggestive of TB and microbiologically confirmed TB, defined as at least one positive smear or MGIT in any sample. Probable TB cases as having at least 1 defined sign or symptom suggestive of TB and a CXR consistent with TB and at least 1 of the following: [1] positive clinical response to TB therapy [3] documented exposure to a household or close contact with a TB case or positive TST. Possible TB cases with at least 1 sign or symptom suggestive of TB and who had either: [1] a CXR that is not consistent with TB and at least 1 of the following: positive clinical response to TB therapy, documented exposure to a household or close contact with a TB case or positive TST or [3] a CXR consistent with TB but none of the other characteristics listed in [1] |
| Outcome | Performance |
| Results | Sensitivity= patient against the clinical gold standard: Smear: 9.2% (12/131) [95%CI 4.2-14.1] MGIT: 29.0% (38/131) [95%CI 21.2-36.8] Xpert: 20.6% (27/131) [95%CI 13.7-27.5] By sample analysis: Smear: 5.4% (14/257) [95%CI 2.2-8.7] MGIT: 23.3% (60/257) [95%CI 16.7-30.0] Xpert: 17.5% (45/257) [95%CI 11.3-23.8] Confirmed TB as the gold standard - Per patient analysis: Xpert: 68.4% (26/38) [95%CI: 53.6-83.2] Confirmed TB as the gold standard - Per sample analysis: Xpert: 57.1% (44/77) [95%CI 42.8-71.5] By sample type: Sputum - Xpert: 41.7% (30/72); Sputum - Smear: 12.5% (9/72); Sputum - MGIT: 50.0% (36/72)  Gastric fluid - Xpert: 7.7% (14/181); gastric fluid - smear: 2.2% (4/181); gastric fluid - MGIT: 13.3% (24/181)  Specificity= Patient against the clinical gold standard Smear: 100% MGIT: 94.7% (18/19) [95%CI 84.7-100] Xpert: 94.7% (18/19) [95%CI 84.7-100] By sample analysis: Smear: 100% (37/37) MGIT: 94.6% (35/37) [95%CI 84.3-100] Xpert: 97.3% (36/37) [95%CI 92.1-100] Confirmed TB as the gold standard - Per patient analysis: Xpert: 98.2% (110/112) [95%CI 95.7-100] Confirmed TB as the gold standard - Per sample analysis: Xpert: 99.1% (215/217) [95%CI 97.8-100]  Other= PPV of Patient against the clinical gold standard: Smear: 100% MGIT: 97.4% (38/39) [95%CI 92.4-100] Xpert: 96.4% (27/28) [95%CI 89.6-1.03] By sample analysis: Smear: 100% (14/14) MGIT: 96.8% (60/62) [95%CI 90.6-100]  Xpert: 97.8% (45/46) [95%CI 93.6-1.02] Confirmed TB as the gold standard - Per patient analysis: Xpert: 92.9% (26/28) [95%CI 83.3-100] Confirmed TB as the gold standard - Per sample analysis: Xpert: 95.7% (44/46) [95%CI 89.7-100] NPV of  Patient against the clinical gold standard Smear: 13.7% (19/138) [95%CI 8.0-19.5] MGIT: 16.2% (18/111) [95%CI 9.4-23.1] By sample analysis: Smear: 13.2% (37/280) [95%CI 7.6-18.8] MGIT: 14.8% (35/232) [95%CI 8.6-21.5] Xpert: 14.5% (36/248) [95%CI 8.4-20.6] Xpert: 14.8% (18/122) [95%CI 8.5-21.1] Confirmed TB as the gold standard - Per patient analysis: Xpert: 90.2% (110/122) [95%CI 84.9-95.5] Confirmed TB as the gold standard - Per sample analysis: Xpert: 86.8% (219/248) [95%CI 81.0-92.6] |

102

| Ref # | 117 |
| --- | --- |
| ID | 817 |
| Disease | Tuberculosis |
| Authors | Al-Ateah SM, Al-Dowaidi MM, El-Khizzi NA |
| Title | Evaluation of direct detection of Mycobacterium tuberculosis complex in Respiratory and non-respiratory clinical specimens using the Cepheid Gene Xpert® system |
| Year | 2012 |
| Language | English |
| Country | Saudi Arabia |
| Type | Peer reviewed paper |
| Design | Cross-sectional |
| Intervention | Cepheid Gene Xpert MTB/RIF |
| Reference test | Microscopy and culture |
| Outcome | Performance |
| Results | Sensitivity= Respiratory samples: Smear = 79.5% (95%CI 74.2-84.7). Xpert = 95.4% (95%CI 89-100) Nonrespiratory samples: Smear = 33.3% (95%CI 31.8 -34.7). Xpert = 94.4% (95%CI 90.2-98.5)  Specificity=Respiratory samples: Smear = 99.2 (95%CI 93.9 -100). Xpert = 100% (95%CI 93.6 -100) Nonrespiratory samples: Smear = 100% (95%CI 98.5-100). Xpert = 100% (95%CI 95.8-100)  Other= PPV In respiratory samples Smear = 97.2% (95%CI 91.9-100). Xpert = 100% (95%CI 93.6-100) Non-respiratory samples Smear=100% (95%CI 98.5-100) Xpert= 100% (95%CI 95.8-100) NPV Respiratory samples: Smear = 93.4% (95%CI 88.1-98.6). Xpert = 98.5% (95%CI 92.1-100). Nonrespiratory samples: Smear = 80% (95%CI 78.5-81.4). Xpert = 98% (95%CI 92.8-100) |

103

| Ref # | 118 |
| --- | --- |
| ID | 1118 |
| Disease | Tuberculosis |
| Authors | Calligaro GL, Theron G, Khalfey H, Peter J, Meldau R, Matinyenya B, et al. |
| Title | Burden of tuberculosis in intensive care units in Cape Town, South Africa, and assessment of the accuracy and effect on patient outcomes of the Xpert MTB/RIF test on tracheal aspirate samples for diagnosis of pulmonary tuberculosis: a prospective burden of disease study with a nested randomised controlled trial |
| Year | 2015 |
| Language | English |
| Country | South Africa |
| Type | Peer reviewed paper |
| Design | Randomised controlled trial |
| Intervention | Xpert MTB/RIF or smear |
| Reference test | Liquid culture BACTEC MGIT 960 system |
| Outcome | Impact and performance |
| Results | Impact= Culture-positive patients started on treatment by 48 h: Smear = 53%. Xpert = 92%. Time to diagnosis (days): Smear = 12.1 (95%CI 0.3-22.2) Xpert = 0.2 (95%CI 0.2-0.3)  Sensitivity= Smear 43% Xpert 100%  Specificity Smear = 100% Xpert = 93.8%  Other= PPV Smear = 100% Xpert = 68.8%. NPV Smear=91.5%. Xpert= 100% Culture-positive patients started on treatment by 48 h: Smear = 53% Xpert=92%. Time to diagnosis (days): Smear = 12.1 (95%CI 0.3-22.2) Xpert = 0.2 (95%CI 0.2-0.3) |

104

| Ref # | 119 |
| --- | --- |
| ID | 1238 |
| Disease | Tuberculosis |
| Authors | Raizada N, Sachdeva KS, Sreenivas A, Kulsange S, Gupta RS, Thakur R, et al. |
| Title | Catching the Missing Million: Experiences in Enhancing TB & DR-TB Detection by Providing Upfront Xpert MTB/RIF Testing for People Living with HIV in India |
| Year | 2015 |
| Language | English |
| Country | India |
| Type | Peer reviewed paper |
| Design | Prospective |
| Intervention | Xpert compared with microscopy and culture |
| Reference test | Smear microscopy, solid or liquid culture |
| Outcome | Performance |
| Results | 770 (27.6%, CI 26.0‒29.3) TB cases were detected on Xpert MTB/RIF, more than 50% being negative on smear microscopy. Remaining, 9 (1.1%) out of the 779 bacteriologically confirmed TB cases were detected on smear microscopy, of which one was not tested and 8 had a negative result for TB on Xpert MTB/RIF. TB positivity rate on Xpert MTB/RIF observed under the study was 27.6% (CI 26‒29.3) against smear positivity rate of 12.9% (CI 11.7‒14.1) Cumulatively, 93% of the cases got test result within 2 days and 99% (2911) had received their results within a week. Overall 89 rifampicin resistant TB cases were diagnosed; 16 (11.2%, CI 6.7‒17.1) being diagnosed among 143 presumptive DR-TB cases and 73 (9.5%, CI 7.6‒11.8) among the 770 positive TB cases detected on Xpert MTB/RIF |

105

| Ref # | 120 |
| --- | --- |
| ID | 1340 |
| Disease | Tuberculosis |
| Authors | Raizada N, Sachdeva KS, Nair SA, Kulsange S, Gupta RS, Thakur R, et al. |
| Title | Enhancing TB case detection: experience in offering upfront Xpert MTB/RIF testing to pediatric presumptive TB and DR TB cases for early rapid diagnosis of drug sensitive and drug resistant TB |
| Year | 2014 |
| Language | English |
| Country | India |
| Type | Peer reviewed paper |
| Design | Prospective |
| Intervention | Xpert MTB/RIF and smear |
| Reference test | Bacteriologically-confirmed or clinically diagnosed case of TB involving the lung parenchyma or the tracheo-bronchial tree with at least one of two smears positive for acid fast bacilli (AFB) using direct microscopy or a patient with symptoms suggestive of TB with two smear examination negative for AFB, with evidence of pulmonary TB by another microbiological method (culture positive or by other approved molecular methods) or chest X-ray. |
| Outcome | Performance |
| Results | The overall positivity rate for Xpert MTB/RIF under the study was 10.4% (95%CI 9.5–11.2) whereas the positivity rate for smear was 4.8% (95%CI 4.2–5.4) Xpert MTB/RIF had higher positivity rates than smear microscopy in all age groups, averaging a 2.2-fold higher proportion of bacterio-  logically-confirmed PTB cases. |

106

| Ref # | 121 |
| --- | --- |
| ID | 1349 |
| Disease | Tuberculosis |
| Authors | Zar HJ, Workman L, Isaacs W, Dheda K, Zemanay W, Nicol MP |
| Title | Rapid diagnosis of pulmonary tuberculosis in African children in a primary care setting by use of Xpert MTB/RIF on respiratory specimens: a prospective study |
| Year | 2013 |
| Language | English |
| Country | South Africa |
| Type | Peer reviewed paper |
| Design | Prospective |
| Intervention | Xpert MTB/RIF or microscopy |
| Reference test | Liquid culture BACTEC MGIT |
| Outcome | Performance |
| Results | PPV Xpert MTB/RIF(first IS specimen): 92.3%. MTB/RIF (both IS specimens)= 84.2%. Xpert MTB/RIF (first NA specimen)= 88.9% Xpert MTB/RIF  (both NA specimens)= 84.6% Microscopy: 75% NPV Xpert MTB/RIF(first IS specimen): 94.6% MTB/RIF (both IS specimens)= 95.9% Xpert MTB/RIF (first NA specimen)= 93.3% Xpert MTB/RIF (both NA specimens)= 94.3 Microscopy:99.6% |

107

| Ref # | 122 |
| --- | --- |
| ID | 1963 |
| Disease | Tuberculosis |
| Authors | Lawn SD, Brooks SV, Kranzer K, Nicol MP, Whitelaw A, Vogt M, et al. |
| Title | Screening for HIV-Associated Tuberculosis and Rifampicin Resistance before Antiretroviral Therapy Using the Xpert MTB/RIF Assay: A Prospective Study |
| Year | 2011 |
| Language | English |
| Country | South Africa |
| Type | Peer reviewed paper |
| Design | Prospective |
| Intervention | Xpert MTB RIF and fluorescent microscopy with auramine O |
| Reference test | Liquid culture MGIT |
| Outcome | Impact and performance |
| Results | Impact= TB cases were diagnosed using the Xpert MTB/RIF assay, increasing case detection by 45.3% (95%CI 32.7–57.9) compared to smear microscopy  Sensitivity= The Xpert MTB/RIF assay detected all smear-positive cases (100% sensitivity, 95%IC 83.9-100). The sensitivity of the Xpert MTB/RIF assay for smear-negative TB was substantially lower than for smear-positive disease and was dependent on the number of sputum samples, with sensitivities of  43.4% (95%CI 29.8-57.7) and 62.3% (95%CI 47.9 -75.2) from one and two samples, respectively. Smear microscopy performed poorly, with one and two samples yielding just 22.2% and 26.4% of TB diagnoses, respectively, compared to 58.3% and 72.2% using the Xpert MTB/RIF assay  Specificity= 99.2% (95%CI 97.7-99.2)  Other= the PPV and NPV of the Xpert MTB/RIF assay were both 94.8%. Rifampicin resistance was correctly identified in all four cases of MDR-TB by the Xpert MTB/RIF assay (100% sensitivity) However, the Xpert MTB/RIF assay also reported rifampicin resistance in three samples from three further patients in which the isolates were reported as rifampicin susceptible using comparator assays |

108

| Ref # | 123 |
| --- | --- |
| ID | 1969 |
| Disease | Tuberculosis |
| Authors | Nicol MP, Workman L, Isaacs W, Munro J, Black F, Eley B, et al. |
| Title | Accuracy of the Xpert MTB/RIF test for the diagnosis of pulmonary tuberculosis in children admitted to hospital in Cape Town, South Africa: a descriptive study |
| Year | 2011 |
| Language | English |
| Country | South Africa |
| Type | Peer reviewed paper |
| Design | Prospective |
| Intervention | Xpert MTB/RIF and concentrated fluorescent acid-fast smear |
| Reference test | Liquid culture BACTEC MGIT |
| Outcome | Performance |
| Results | Sensitivity= MTB/RIF General was 58.7% (95%CI 45.6 - 71.1) for the firs induced sputum and 75.9% (95%CI64.5 - 87.2) for both induced sputum. In HIV infected patients sensitivity of 100% (95%CI 76.8 -100) and in HIV uninfected 68.2% (95%CI 53.9 - 82.5). The sensitivity for smear positive definite tuberculosis was 100% (95%CI 84.6 - 100) and for smear negative 33.3% (95%CI 17.2 -49.5) for the first specimen and 61.1 for both specimens (95%CI 44.4 - 77.8)  Specificity=MTB/RIF General was 99.5% (95%CI 98.5-100) for the first induced sputum and 98.8% (95%CI 97.6 - 99.9) for both specimens. In HIV infected patients the specificity was 100% (95%CI 95.5 -100) and In HIV uninfected was 98.4% (95%CI 96.8 - 99.9)  Other= MTB/RIF PPV General 94.4% for the first specimen and 91.7 for both In HIV infected patients the PPV was 100% and in uninfected patients was 88.2%   MTB/RIF NPV General 93.1%. for both specimens 95.8% in HIV infected patientes 100% and in HIV uninfected patients 94.5% |

109

| Ref # | 124 |
| --- | --- |
| ID | 3634 |
| Disease | Tuberculosis |
| Authors | Bholla M, Kapalata N, Masika E, Chande H, Jugheli L, Sasamalo M, et al. |
| Title | Evaluation of Xpert® MTB/RIF and Ustar EasyNAT™ TB IAD for diagnosis of tuberculous lymphadenitis of children in Tanzania: a prospective descriptive study |
| Year | 2016 |
| Language | English |
| Country | Tanzania |
| Type | Peer reviewed paper |
| Design | Prospective |
| Intervention | Xpert MTB/RIF, EasyNAT and microsocopy |
| Reference test | Composite reference of liquid culture MGIT and/or citology |
| Outcome | Performance |
| Results | Sensitivity= Compared with composite reference estándar (Culture and/or cytology) ZN= 14% Xpert=58% EasyNAT=19%  Specificity= Compared with composite reference estándar (Culture and/or cytology) ZN=100% Xpert=93% EasyNAT=100%  Other= the mixed logistic regression model returned a TBscore for each patient. The correlation of this score with each binary test showed that cytology performed best with a correlation of r = 0.87, followed by culture and Xpert MTB/RIF at par (r = 0.82), EasyNAT (r = 0.60) and ZN (r = 0.42) |

110

| Ref # | 125 |
| --- | --- |
| ID | 564 |
| Disease | Tuberculosis |
| Authors | Held M, Laubscher M, Mears S, Dix-Peek S, Workman L, Zar H, et al. |
| Title | Diagnostic accuracy of the Xpert MTB/RIF assay for extrapulmonary tuberculosis in children with musculoskeletal infections |
| Year | 2016 |
| Language | English |
| Country | South Africa |
| Type | Peer reviewed paper |
| Design | Cross-sectional |
| Intervention | Xpert |
| Reference standard | Culture on BACTEC MGIT 960 and histology (ZN and HE stains) |
| Outcome | Performance |
| Results | Sensitivity= comparing Xpert to a gold standard of culture or histology: 73.9% (95%CI 51.6-89.8) TB culture compared with histology confirmed TB: 60.9% (95%CI 38.5-80, p=0.345) Smear compared with culture or histology: 60.9% (95%CI 38.5-80.3) Evidence of acid-fast bacilli on ZN stain only: 65.2% (95%CI 42.7-83.6) Histology: 100% (95%CI 85.7-100.0)  Specificity= comparing Xpert to a gold standard o culture or histology: 100% (95%CI95.7-100) TB culture compared with histology confirmed TB: 100% (95%CI 95.7-100) Smear compared with culture or histology: 98.8% (95%CI 93.6-99.9)  Evidence of acid-fast bacilli on ZN stain only: 100.0% (95%CI 95.4-100.0) Histology: 100% (95%CI 95.5-100.0)  Other= PPV comparing Xpert to a gold standard o culture or histology: 100% (95%CI 81.6-100) Evidence of acid-fast bacilli on ZN stain only: 100.0% (95%CI 79.6-100.0) Histology: 100% (95%CI 85.7-100.0) NPV comparing Xpert to a gold standard o culture or histology: 93.5% (95%CI 86.3-97.6) Evidence of acid-fast bacilli on Ziehl-Nielson stain only: 91.4% (95%CI 83.8-96.2) Histology: 100.0% (95%CI 95.5-100.0) |

111

| Ref # | 126 |
| --- | --- |
| ID | 3407 |
| Disease | Tuberculosis |
| Authors | Okumu A, |
| Title | Comparison of performance between Ziehl Neelsen (ZN) microscopy and the Xpert MTB/RIF assay in detection of M. tuberculosis in sputum at KEMRI/CDC TB lab |
| Year | 2012 |
| Language | English |
| Country | Kenya |
| Type | Conference abstract |
| Design | Cannot tell |
| Intervention | Xpert MTB/RIF |
| Reference test | ZN microscopy |
| Outcome | Performance |
| Results | Using both ZN microscopy and Xpert MTB/RIF, 34 (13%) were positive by Xpert MTB/RIF and eight (3%) were positive by ZN microscopy. ZN microscopy identified only eight of 34 (24%) positive specimens identified by Xpert MTB/RIF. |

112

| Ref # | 127 |
| --- | --- |
| ID | 3803 |
| Disease | Tuberculosis |
| Authors | Dhasmana DJ, Ross C, Bradley CJ, Connell DW, George PM, Singanayagam A, et al. |
| Title | Performance of Xpert MTB/RIF in the diagnosis of tuberculous mediastinal lymphadenopathy by endobronchial ultrasound |
| Year | 2013 |
| Language | English |
| Country | United Kingdom |
| Type | Peer reviewed paper |
| Design | Cannot tell |
| Intervention | Xpert MTB/RIF, microscopy ZN and auramine phenol, and citology |
| Reference test | Liquid culture BACTEC MGIT 960 |
| Outcome | Performance |
| Results | Sensitivity= Xpert demonstrated an overall sensitivity for culture-positive TB  of 72.6% (95%CI 62.3–81.0)  Specificity= Xpert 96.3% (95%CI 89.1–99.1)  Other= PPV 88.9% (95%CI 69.7-97.1) NPV 86.5% (95%CI 76.9-92.1). PLR: 17.8 (95%CI 5.8-55.9) NLR 0.35 (95%CI 0.2-0.6) |

113

| Ref # | 128 |
| --- | --- |
| ID | 3773 |
| Disease | Tuberculosis |
| Authors | Kerkhoff AD, Wood R, Vogt M, Lawn SD. |
| Title | Predictive value of anemia for tuberculosis in HIV-infected patients in Sub-Saharan Africa: An indication for routine microbiological investigation using new rapid assays |
| Year | 2014 |
| Language | English |
| Country | South Africa |
| Type | Peer reviewed paper |
| Design | Prospective |
| Intervention | Xpert MTB RIF, flurorescent microscopy with auramine O, and TB-LAM |
| Reference test | Liquid culture |
| Outcome | Performance |
| Results | Sensitivity= compared with liquid culture: Microscopy 30% (95%CI 20-41), Xpert: 58% (95%CI 47-69), LAM: 28% (19-39), LAM + microscopy: 44% (95%CI 33-56), LAM+Expert: 61% (95%CI 50-72%)  The specificity of all assays either in isolation or in combination was greater than 98% and 97%, respectively, and did not differ according to the severity of anemia  Compared with liquid culture: PPV Microscopy: 96% (95%CI 78-100), Xpert: 92% (95%CI 80-98), LAM: 79% (95%CI 60-91%), LAM + microscopy: 83,7% (95%CI 69-93), LAM + Xpert: 83% (95%CI 71-91). NPV Microscopy: 88% (95%CI 84-90), Xpert: 92% (95%CI 89-94), LAM: 87% (95%CI 84-90), LAM + microscopy: 90 (95%CI 87-92), LAM + Xpert: 93% (95%CI 90-95) |

114

| Ref # | 129 |
| --- | --- |
| ID | 649 |
| Disease | Tuberculosis |
| Authors | Gous N, Scott LE, Khan S, Reubenson G, Coovadia A, Stevens W |
| Title | Diagnosing childhood pulmonary tuberculosis using a single sputum specimen on Xpert MTB/RIF at point of care |
| Year | 2015 |
| Language | English |
| Country | South Africa |
| Type | Peer reviewed paper |
| Design | Cannot tell |
| Intervention | Xpert MTB RIF and smear miscroscopy |
| Reference test | Liquid culture MGIT |
| Outcome | Performance |
| Results | Sensitivity= Xpert: 0 - 6 months: 0% (95%CI 0-97.5) 7 - 12 months: 0% CI 95% (95%CI 0-97.5) 13 - 24 months: 100% (95%CI 2.5-100) 25 - 60 months: - >60 months: 66.7% (95%CI 22.3-95.7) Culture as the reference Smear microscopy: 33.3% (95%CI 7.9-69.9) Xpert: 62.5% (95%CI 24.7-91.0)  Specificity=Xpert: 0 - 6 months: 100% (95%CI 91.2-100) 7 - 12 months: 98.9% (95%CI 94.5-99.9) 13 - 24 months: 100% (95%CI 94.8-100) 25 - 60 months: 100% (95%CI 92.6-100) >60 months: 97.5% (95%CI 91.4-99.7) Culture as the reference  Smear microscopy: 99.5% (95%CI 98.0-99.9) Xpert: 99.1% (95%CI 97.4-99.8)  Other= Xpert: PPV 0 - 6 months: - 7 - 12 months: 0% (95%CI 0-97.5) 13 - 24 months: 100% (95%CI 2.5-100) 25 - 60 months: - >60 months: 66.7% (95%CI 22.3-95.7) NPV 0 - 6 months: 97.6% (95%CI 87.1-99.9) 7 - 12 months: 98.9% (95%CI 94.5-99.9) 13 - 24 months: 100% (95%CI 94.8-100) 25 - 60 months: 100% (95%CI 92.6-100) >60 months: 97.5% (95%CI 91.4-99.7) Culture as the reference  PPV: Smear microscopy: 66.0% (95%CI 15.4-93.5); Xpert: 62.5% (95%CI 24.7-91.0) NPV: Smear microscopy: 98.4% (95%CI 96.5-99.4); Xpert: 99.1% (95%CI 97.4-99.8) |

115

| Ref # | 130 |
| --- | --- |
| ID | 6704 |
| Disease | Tuberculosis |
| Authors | Nhu NTQ, Heemskerk D, Thu DDA, Chau TTH, Mai NTH, Nghia HDT, et al. |
| Title | Evaluation of GeneXpert MTB/RIF for diagnosis of tuberculous meningitis |
| Year | 2014 |
| Language | English |
| Country | Viertnam |
| Type | Peer reviewed paper |
| Design | Randomized controlled trial |
| Intervention | Xpert MTB/RIF, ZN smear, and MGIT culture |
| Reference test | Clinical case definition based on: clinical, CSF, cerebral imaging, evidence of tuberculosis elsewhere, exclusion of alternative diagnosis criteria |
| Outcome | Performance |
| Results | Sensitivity= Xpert compared to clinical diagnosis of TBM: 59.3% (108/182; 95%CI 51.8-66.5) Smear relative to final clinical diagnosis: 78.6% (143/182; 95%CI 71.9-84.3) MGIT culture to final clinical diagnosis: 66.5% (121/182; 95%CI 59.1-73.3)  Xpert MTB/RIF relative to smear: 73.4% (105/143; 95%CI 65.4-80.5) Xpert MTB/RIF relative to MGIT culture: 85.1% (103/121; 95%CI 77.5-90.9) Xpert: 50.0% (13/26; 95%CI 29.9-70.1) Smear: 88.5% (23/26; 95%CI 69.8-97.6) MGIT culture: 57.7% (15/26; 95%CI 36.9-76.6) Xpert for the “definite TBM” result: 54.2% (13/24; 95%CI 32.8-74.4) Xpert with addition of a vortexing step: 60.9% (95/156; 95%CI 52.8-68.6) Smear with addition of a vortexing step: 76.9% (120/157; 95%CI 69.5-83.3) MGIT culture with addition of a vortexing step: 67.9% (106/156; 95%CI 60.0-75.2) Xpert for the “definite TBM” result: 74.8% (95/127; 95%CI 66.3-82.1) Xpert MTB/RIF for low-volume samples: 51.7% (15/29; 95%CI 32.5-70.6) Xpert MTB/RIF for medium-volume samples: 61.5% (64/104; 95%CI 44.2-73.0) Xpert MTB/RIF for high-volume samples: 59.2% (29/49; 95%CI 44.2-73.0) Xpert for TBM in HIV patients: 78.8% (52/66; 95%CI 77.6-79.7) Xpert for TBM in non-HIV-infected patients: 47.9% (45/94; 95%CI 47.0-48.7)  Specificity= Xpert compared to clinical diagnosis of TBM 99.5% (95%CI 97.2-100)  Other=Xpert against final clinical diagnosis of TBM PPV 99.1% (108/109; 95%CI 95.0-100) NPV 72.5% (196/270; 95%CI 66.9-77.8) Xpert for TBM against clinical diagnosis for HIV infection: OR=4,01 (95%CI 3.65-4.36; p<0,001) |

116

| Ref # | 131 |
| --- | --- |
| ID | 4545 |
| Disease | Tuberculosis |
| Authors | Zar HJ, Workman L, Boehme C, Eley B, Nicol MP |
| Title | Cartridge-Based Automated Nucleic Acid Amplification Test (xpert Mtb/rif) For The Diagnosis Of Pulmonary Tuberculosis In Hiv-Infected And Uninfected Children: A Prospective Study |
| Year | 2011 |
| Language | English |
| Country | South Africa |
| Type | Conference abstract |
| Design | Prospective |
| Intervention | Xpert MTB/RIF and smear |
| Reference test | Culture |
| Outcome | Performance |
| Results | Sensitivity= Xpert compared with culture 74.3% (95%CI 64.1-84.5). HIV positive patients: 100%. HIV uninfected: 66%  Specificity Xpert compared with culture: 98.2% ([95%CI 96.9-99.5). HIV positive patients: 99%. HIV uninfected: 98%  Other= the incremental yield from a second IS sample over the first was 8 cases (14%) using culture and 12 cases (25%) using Xpert MTB/Rif. Xpert MTB/RIF provided more rapid results than culture [median time to positive Xpert 1 (interquartile range 0-3) days compared to 14 (11-20) for culture |

117

| Ref # | 132 |
| --- | --- |
| ID | 6746 |
| Disease | Tuberculosis |
| Authors | Peñata A, Salazar R, Castaño T, Bustamante J, Ospina S |
| Title | Molecular diagnosis of extrapulmonary tuberculosis and sensitivity to rifampicin |
| Year | 2016 |
| Language | Spanish |
| Country | Colombia |
| Type | Peer reviewed paper |
| Design | Cross-sectional |
| Intervention | Xpert® MTB/RIF and ZN microscopy |
| Reference test | Ogawa-Kudoh culture |
| Outcome | Performance |
| Results | Sensitivity= Xpert MTB/RIF vs culture: 93.5% (95%CI 83.2-100) Xpert MTB/RIF vs baciloscopy: 100% (95%CI 95-100) Baciloscopy vs culture: 38.7% 95% CI (95%CI 19.5-57.4) Xpert® MTB / RIF test according to culture and sample type sterile Liquids Cerebrospinal fluid: 100% (95%CI 91-100) Pleural fluid: 100% (95%CI 75-100) Synovial fluid: 100% (95%CI 50-100) Peritoneal fluid: 100% (95%CI 50-100) Tissues Ganglion: 75% (95%CI 20-100) Lung: 100% (95%CI 83-100) Bone: 100% (95%CI 50-100) Not Specified: 100% (95%CI 95-100) Others Total: 94% (95%CI 83-100)  Specificity= Xpert MTB/RIF vs culture: 97% (95%CI 95.1-99) Xpert MTB/RIF vs baciloscopy: 92% (95%CI 89-95) Baciloscopy vs culture: 100% (95%CI 99.8-100)  Xpert® MTB / RIF test according to culture and sample type sterile Liquids  Cerebrospinal fluid: 99% (95%CI 97-100) Pleural fluid: 100% (95%CI 98-100)  Synovial fluid: 100% (95%CI 87-100) Peritoneal fluid: 100% (95%CI 96-100)  Tissues Ganglion: 66% (95%CI 0-100) Lung: 100% 95%CI 50-100) Bone: 100% (95%CI 87-100) Not specified: 94% (95%CI 90-99) others total: 97% 95% CI (95%CI 95-99)  Other= Baciloscopy vs. culture: kappa 0.5366 (95%CI 0.3567-0.7165) Xpert® MTB / RIF Vs. Baciloscopy: kappa 0.4431 (95%CI 0.2751-0.6111) Xpert® MTB / RIF Vs. farming: kappa 0.811 (95%CI 0.7074-0.9147) Xpert MTB/RIF vs culture: PPV 74.3% (95%CI 59.3-89.3); NPV 99.4% (95%CI 98.4-100) |

118

| Ref # | 133 |
| --- | --- |
| ID | 4596 |
| Disease | Tuberculosis |
| Authors | Wang Y, Williams C, Merritt S, Kasinathan V, Young AN |
| Title | Evaluation of GenXpert MTB/RIF assay on AFB-smear positive respiratory samples |
| Year | 2010 |
| Language | English |
| Country | Cannot tell |
| Type | Conference abstract |
| Design | Cannot tell |
| Intervention | GeneXpert MTB/RIF and MTB Direct |
| Reference test | Culture |
| Outcome | Performance |
| Results | Sensitivity= MTB/RIF assay compared to culture: 96.2%  Specificity= MTB/RIF assay compared to culture: 100%  Other= the incremental yield from a second IS sample over the first was 8 cases (14%) using culture and 12 cases (25%) using Xpert MTB/Rif. Xpert MTB/RIF provided more rapid results than culture [median time to positive Xpert 1 (interquartile range 0-3) days compared to 14 (11-20) for culture] |

119

| Ref # | 134 |
| --- | --- |
| ID | 4073 |
| Disease | Tuberculosis |
| Authors | Boehme CC, Nicol MP, Nabeta P, Michael JS, Gotuzzo E, Tahirli R, et al. |
| Title | Feasibility, diagnostic accuracy, and eﬀectiveness of decentralised use of the Xpert MTB/RIF test for diagnosis of tuberculosis and multidrug resistance: a multicentre implementation study |
| Year | 2011 |
| Language | English |
| Country | South Africa, Peru, Azerbaijan, Philippines, Uganda and India |
| Type | Peer reviewed paper |
| Design | Quantitative randomized controlled trial prospective study." |
| Intervention | Xpert MTB/RIF |
| Reference test | Microscopy and solid (Lowestein-Jensen or Ogawa-Kudoh) or liquid ( BACTEC MGIT) culture |
| Outcome | Performance |
| Results | Sensitivity of MTB/RIF in rifampicin-resistant cases compared with culture: 94.4% (95%CI 90.8–96.6)  Specificity of MTB/RIF in rifampicin-sensitive cases compared with culture: 98.3% (95%CI 97.1–99.0)  PPV of MTB/RIF 93.2%; NPV 98.6% |

120

| Ref # | 135 |
| --- | --- |
| ID | 3805 |
| Disease | Tuberculosis |
| Authors | Pandie S, Peter JG, Kerbelker ZS, Meldau R, Theron G, Govender U, et al. |
| Title | Diagnostic accuracy of quantitative PCR (Xpert MTB/RIF) for tuberculous pericarditis compared to adenosine deaminase and unstimulated interferon-γ in a high burden setting: a prospective study |
| Year | 2014 |
| Language | English |
| Country | South Africa |
| Type | Peer reviewed paper |
| Design | Prospective |
| Intervention | Xpert MTB/RIF, ADA and uIFNγ assays |
| Reference test | Liquid culture and histology |
| Outcome | Performance |
| Results | Sensitivity= Xpert MTB/RIF: 63.8% (95%CI 52.4-75.1) uIFNγ: 95.7% (95%CI 88.1-98.5) ADA (rule-in cut-point: >107 IU/ml): 15.7% (95%CI 9.0-26.0) ADA (cut-point in current clinical use: ≥35 IU/ml): 95.7% (88.1-98.5) Tygerberg score ≥6: 85.3% (95%CI 75.9-81) Clinical predictors (rule-in cut-point: >6.1): 60.8% (95%CI 49.4-71.1) Clinical predictors (Youden’s and rule-out cut-point: >3.5): 91.9% (95%CI 83.4-96.2) Xpert MTB combined with uIFNγ (with uIFNγ if Xpert MTB/RIF negative): 97.1% (95%CI 89.9-99.2) Xpert MTB combined with ADA (with ADA if Xpert MTB/RIF negative): 98.4% 95% CI (95%CI 91.7-99.7)  Specificity=Xpert MTB/RIF: 100% (95%CI 85.6-100) uIFNγ: 96.3% (95%CI 81.7-99.3) ADA (rule-in cut-point: >107 IU/ml): 96% (95%CI 80.5-99.3) ADA (cut-point in current clinical use: ≥35 IU/ml): 84% (95%CI 65.4-93.6) Tygerberg score ≥6: 77.3% (95%CI 56.6-89.9) Clinical predictors (rule-in cut-point: >6.1): 96.3% (95%CI 81.7-99.3) Clinical predictors (Youden’s and rule-out cut-point: >3.5): 81.5% (95%CI 63.3-91.8) Xpert MTB combined with uIFNγ (with uIFNγ if Xpert MTB/RIF negative): 100% 95% CI (95%CI 86.7-100) Xpert MTB combined with ADA (with ADA if Xpert MTB/RIF negative): 100% 95% CI (95%CI 85.7-100)  Other= LR+ uIFNγ: 25.8 (95%CI 3.6-184) ADA (rule-in cut-point: >107 IU/ml): 3.93 (95%CI 0.21-72.5) ADA (cut-point in current clinical use: ≥35 IU/ml): 6.0 (95%CI 3.7-9.8)  Tygerberg score ≥6: 3.75 (95%CI 2.52-5.59) Clinical predictors (rule-in cut-point: >6.1): 16.4 (95%CI 2.25-119.9) Clinical predictors (Youden’s and rule-out cut-point: >3.5): 4.96 (95%CI 3.34-7.36)  LR- Xpert MTB/RIF: 0.36 (95%CI 0.33-0.39) uIFNγ: 0.045 (95%CI 0.023-0.09)  ADA (rule-in cut-point: >107 IU/ml): 0.88 (95%CI 0.85-0.91) ADA (cut-point in current clinical use: ≥35 IU/ml):0.051 (95%CI 0.026-0.10) Tygerberg score ≥6: 0.19 (95%CI 0.15-0.24) Clinical predictors (rule-in cut-point: >6.1): 0.41 (95%CI 0.38-0.44) Clinical predictors (Youden’s and rule-out cut-point: >3.5): 0.10 (95%CI 0.07-0.14) Xpert MTB combined with uIFNγ (with uIFNγ if Xpert MTB/RIF negative): 0.03 (95%CI 0.01-0.08) Xpert MTB combined with ADA (with ADA if Xpert MTB/RIF negative): 0.02 (95%CI 0.002-0.11)  PPV: Xpert MTB/RIF: 100% (95%CI 98.0-100) uIFNγ: 91.7% (95%CI 88.1-94.3) ADA (rule-in cut-point: >107 IU/ml): 62.7% (95%CI 51.4-72.8) ADA (cut-point in current clinical use: ≥35 IU/ml): 71.9% (95%CI 67.3-76.1) Tygerberg score ≥6: 61.7% (95%CI 56.9-66.2) Clinical predictors (rule-in cut-point: >6.1): 87.6% (95%CI 82.4-91.4) Clinical predictors (Youden’s and rule-out cut-point: >3.5): 68.0% (95%CI 63.3-72.4) Xpert MTB combined with uIFNγ (with uIFNγ if Xpert MTB/RIF negative): 100% (95%CI 98.7-100) Xpert MTB combined with ADA (with ADA if Xpert MTB/RIF negative): 100% (95%CI 98.7-100)  NPV= Xpert MTB/RIF: 86.6% (95%CI 84.0-88.7) uIFNγ: 98.1% (95%CI 96.8-98.9) ADA (rule-in cut-point: >107 IU/ml): 72.7% (95%CI 69.7-75.4) ADA (cut-point in current clinical use: ≥35 IU/ml): 97.9% (95%CI 96.4-98.7) Tygerberg score ≥6: 92.5% (95%CI 90.0-94.3) Clinical predictors (rule-in cut-point: >6.1): 85.1% (95%CI 82.5-87.5) Clinical predictors (Youden’s and rule-out cut-point: >3.5): 95.9% (95%CI 94-97.2) Xpert MTB combined with uIFNγ (with uIFNγ if Xpert MTB/RIF negative): 98.8% (95%CI 97.7-99.4) Xpert MTB combined with ADA (with ADA if Xpert MTB/RIF negative): 99.3% (95%CI 98.4-99.7) |

121

| Ref # | 136 |
| --- | --- |
| ID | 981 |
| Disease | Tuberculosis |
| Authors | Naidoo P, Dunbar R, Lombard C, du Toit E, Caldwell J, Detjen A, et al. |
| Title | Comparing tuberculosis diagnostic yield in smear/culture and Xpert MTB/Rif-based algorithms using a non-randomised stepped-wedge design |
| Year | 2016 |
| Language | English |
| Country | South Africa |
| Type | Peer reviewed paper |
| Design | Non-randomized trial |
| Intervention | Prior to Aug 2011 smear/culture algorithm from Aug 2011 to Feb 2013 Xpert based algorithm |
| Reference test | Fluorescent microscopy with Auramine and liquid culture BACTEC MGIT |
| Outcome | Performance |
| Results | Proportion of TB cases: 20.9% (95%CI 19.9 to 22) in Smear/culture algorithm and 17.9 (95%CI 16.4 to 19.5) in Xpert algorithm, results similar after adjusting for MDR. Positivity declined over time from 23.6% (22.2 to 25.1) at T1 to 17.5% (15 to 20) at T7. After adjusting for time there were not differences in positivity results of algorithms with 19.1% (17.6 to 20.5) in smear/culture and 19.3% (17.7 to 20.9) in Xpert algorithm) |

122

| Ref # | 137 |
| --- | --- |
| ID | 295 |
| Disease | Tuberculosis |
| Authors | O'Donnell MR, Pym A, Jain P, Munsamy V, Wolf A, Karim F, et al. |
| Title | A Novel Reporter Phage To Detect Tuberculosis and Rifampin Resistance in a High-HIV-Burden Population |
| Year | 2015 |
| Language | English |
| Country | South Africa |
| Type | Peer reviewed paper |
| Design | Cannot tell |
| Intervention | Phage assay and Xpert |
| Reference test | “composite reference standard” and liquid MGIT culture |
| Outcome | Performance |
| Results | Sensitivity= Phage assay: 95.90 (95%CI 90.69–98.64). Xpert: 94.12 (95%CI 88.25–97.59)  Specificity= Phage assay: 83.33 (95%CI 67.18–93.59) Xpert: 97.22 (95%CI 85.42–99.54).  Detection of rifampin resistance: Sensitivity: Phage assay: 86.67% (95%CI 69.26–96.16), Xpert: 83.33% (95%CI 62.60–90.97). Specificity: Phage assay: 88.28% (95%CI 81.41–93.29), Xpert: 95.16% (95%CI 94.19–99.75). |

123

| Ref # | 138 |
| --- | --- |
| ID | 3990 |
| Disease | Tuberculosis |
| Authors | Lawn SD, Kerkhoff AD, Vogt M, Wood R |
| Title | Diagnostic accuracy of a low-cost, urine antigen, point-of-care screening assay for HIV-associated pulmonary tuberculosis before antiretroviral therapy: a descriptive study |
| Year | 2011 |
| Language | English |
| Country | South Africa |
| Type | Peer reviewed paper |
| Design | Cannot tell |
| Intervention | Determine TB LAM and Clearview TB ELISA |
| Reference test | Automated liquid MGIT culture, fluorescent microscopy with auramine O, Xpert MTB RIF |
| Outcome | Performance |
| Results | Sensitivity: TB-LAM compared with culture 28.2% (95%CI 19.0–39.0), TB-LAM compared with Xpert MTB/RIF: 57.6% (IC95% 46.4–68.3)  Specificity: TB-LAM compared with culture 98.6% (95%CI 97.0–99.5), TB-LAM compared with Xpert MTB/RIF: 99.1% (95%CI 97.6–99.7) |

124

| Ref # | 139 |
| --- | --- |
| ID | 161 |
| Disease | Tuberculosis |
| Authors | Cox HS, Daniels JF, Muller O, Nicol MP, Cox V, van Cutsem G, et al. |
| Title | Impact of decentralized care and the Xpert MTB/RIF test on rifampicin-resistant tuberculosis treatment initiation in Khayelitsha, South Africa |
| Year | 2015 |
| Language | English |
| Country | South Africa |
| Type | Peer reviewed paper |
| Design | Retrospective |
| Intervention | Complex intervention including: Patients counseling, home visits, training of health staff, descentralized model of care, liquid culture, Line probe assay and Xpert |
| Outcome | Impact |
| Results | During the Line probe assay implementation the median time to treatment TTT of patients with rifampicin resistant TB was 25 days (16-36). Xpert was implemented at the end of 2011 and was associated with a further decline in TTT to a median of 8 days in 2013 (P < .0001) |

125

| Ref # | 140 |
| --- | --- |
| ID | 338 |
| Disease | Tuberculosis |
| Authors | Maghimbi A, Majigo M, Mashinji V, Loy G, Mwakyusa S |
| Title | Challenges fueling the complexities of TB diagnosis & TBHIV comorbidity in Tanzania- IHV experience |
| Year | 2013 |
| Language | English |
| Country | Tanzania |
| Type | Conference abstract |
| Design | Cannot tell |
| Intervention | Installing GeneXpert machine |
| Outcome | Impact |
| Results | Increase of 6.6% TB cases that were missed by fluorescent microscopy |

126

| Ref # | 141 |
| --- | --- |
| ID | 348 |
| Disease | Tuberculosis |
| Authors | Lawn SD, Kerkhoff A, Burton R, Schutz C, Van Wyk G, Vogt M, et al. |
| Title | Massive diagnostic yield of HIV-associated tuberculosis using rapid urine assays in South Africa |
| Year | 2014 |
| Language | English |
| Country | South Africa |
| Type | Conference abstract |
| Design | Observational |
| Intervention | Fluorescent microscopy, liquid culture, Xpert MTB/RIF and TB LAM |
| Outcome | Impact |
| Results | Using samples obtained in the first 24-hours, the proportions of final diagnoses made by sputum microscopy, sputum-Xpert, urine-LAM and urine-Xpert (30-40 ml concentrated urine) were 19.4%, 26.6%, 38.1% and 59.0%, respectively. Rapid urine tests used together diagnosed 69.1% (96 of 139) of cases. This further increased to 80.6% (112 of 139) of cases when combined with sputum Xpert testing |

127

| Ref # | 142 |
| --- | --- |
| ID | 532 |
| Disease | Tuberculosis |
| Authors | Ssengooba W, Respeito D, Mambuque E, Blanco S, Bulo H, Mandomando I, et al. |
| Title | Do xpert MTB/RIF cycle threshold values provide information about patient delays for tuberculosis diagnosis? |
| Year | 2016 |
| Language | English |
| Country | Mozambique |
| Type | Peer reviewed paper |
| Design | Prospective |
| Intervention | Xpert, AFB smear, Ziehl Neelsen microscopy, Culture |
| Outcome | Impact |
| Results | Distribution of patient delay and Ct values: symptom duration showed a median of 30 days and an IQR of 30-45 days. 37% had medium (16–22 cycles) Xpert Ct values. There was no correlation between Ct values and delays (Spearman R^2^ = 0.001. p = 0.612) Log-transforming the delays did not improve the correlation (R^2^ <0.001. p = 0.798) Multivariate predictors of patient delay for TB diagnosis: Ct 23-28 cycles: OR 0.99 (95%CI 0.504-1.962). p value 0.987, Ct 16-22 cylces: OR 0.93 (95%CI 0.499-1.745). p value 0.828, Ct<16 cycles: OR 1.05 (95%CI 0.471-2.362) p value 0.897, HIV-negative: OR 1.74 (95%CI 1.193-3.512) p value 0.009 |

128

| Ref # | 143 |
| --- | --- |
| ID | 1083 |
| Disease | Tuberculosis |
| Authors | Hoang TTT, Nguyen NV, Dinh SN, Nguyen HB, Cobelens F, Thwaites G, et al. |
| Title | Challenges in detection and treatment of multidrug resistant tuberculosis patients in Vietnam |
| Year | 2015 |
| Language | English |
| Country | Vietnam |
| Type | Peer reviewed paper |
| Design | Cannot tell |
| Intervention | Xpert MTB/RIF |
| Outcome | Impact, adoption and penetration |
| Results | Impact= In 2013 there were 5065 (95%CI 3355–6700) MDR-TB patients among 102,196 notified TB cases. The proportion of MDR-TB presumptive cases tested by Xpert MTB/RIF was 31.2 % (5668/18,165) for the whole country and 37.8% (5668/14,998) in the 35 PMDT provinces. Among 5668 MDR-TB presumptive cases tested, 997 cases were detected as rifampicin resistant (17.6 %). Retreatment patients who showed no sputum conversion after 3 months of the category II regimen were tested frequently: 85.7 % (413/482). Of 997 rifampicin-resistant cases detected, 948 (95.1 %) were enrolled for MDR-TB treatment, accounting for just 18.7 % (95%CI 14.1–28.3) of the estimated 5065 MDR-TB cases in the whole of Vietnam. In the 35 PMDT provinces, the enrollment proportion of MDR-TB cases was low: 948/3982 (23.8 %; 95%CI18.0–35.9).  Adoption= Obstacle: a set of key documents is lacking: updated guidelines, concise and clear SOPs, and standard training modules. Proposed solution: The NTP is strongly recommended to ensure that key documents are prepared and circulated to appropriate staff, with proper training. Letters with updates should be discouraged, unless there is an urgency. Obstacle: Failures in identifying presumptive MDR-TB cases for screening. Proposed solution: The development of consistent training modules in accordance with national guidelines and SOPs.  Obstacle: Current Vietnam policy is to require patient to be hospitalized at the start of treatment. However, there is insufficient hospitalization capacity and patients may refuse to be referred to another treatment centre in another province due to distance from either hometown or additional costs without getting health insurance reimbursement. Proposed solution: The NTP policy needs consider the adoption for ambulatory treatment with community-based care as also recommended by WHO. Health insurance need to support MDR-TB patients in reimbursing costs in case there is a need for referral to another province for PMDT treatment. Obstacle: poor links between the NTP and public sector and no management system for MDR-TB patients in prison. There is no mechanism to refer MDR-TB presumptives or MDR-TB patients from the private sector to the NTP to be diagnosed or for treatment. The private sectors often do not notify MDR-TB cases to the NTP. Proposed solution: Establish the collaboration between the private sector and PMDT. Ensure private sector adheres to treatment guidelines for TB and MDR-TB. Provide diagnosis and treatment service for MDR-TB patients in prison.  Penetration= obstacle: Absence of a sound referral system for sending sputum samples to a laboratory. Proposed solution: A sound national referral system should be set up with a shipping agency who can do this safely. Obstacle: Temporary MDR-TB drug stock-out due to procurement and distribution delay resulted in patients either not enrolled for treatment or a delay in treatment. Proposed solution: Improve drug procurement and distribution system. Obstacle: TB units in many districts remain located in health centers that focus on prevention, and are separate from the general hospitals, which is discrepant from MoH policy. Proposed solution: Enforce policy to locate TB units in the district general hospitals. Training should be provided to appropriate staff. |

129

| Ref # | 144 |
| --- | --- |
| ID | 3698 |
| Disease | Tuberculosis |
| Authors | Trajman A, Durovni B, Saraceni V, Menezes A, Cordeiro-Santos M, Cobelens F, et al. |
| Title | Impact on patients' treatment outcomes of XpertMTB/RIF implementation for the diagnosis of tuberculosis: Follow-up of a stepped-wedge randomized clinical trial |
| Year | 2015 |
| Language | English |
| Country | Brazil |
| Type | Peer reviewed paper |
| Design | Stepped-wedge randomized clinical trial |
| Intervention | Smear microscopy and Xpert MTB/RIF |
| Outcome | Impact |
| Results | Impact= Patients’ treatment outcomes in the baseline and intervention arm, stratified by type of diagnosis: overall Sucessful: baseline 1267 (68.3%), intervention 1571 (70.4%). Loss to follow-up: baseline 300 (16.2%), intervention 356 (15.9%). TB-attributed death: baseline 71 (3.8%), intervention 52 (2.3%). Other deaths: baseline 38 (2.0%), intervention 36 (1.6%). Transfer out: baseline 145 (7.8%), intervention 160 (7.2%). Change of diagnosis: baseline 16 (0.9%), intervention 18 (0.8%). Resistance: baseline 19 (1.0%), intervention 39 (1.7%) |

130

| Ref # | 145 |
| --- | --- |
| ID | 3718 |
| Disease | Tuberculosis |
| Authors | Churchyard GJ, Stevens WS, Mametja LD, McCarthy KM, Chihota V, Nicol MP, et al. |
| Title | Xpert MTB/RIF versus sputum microscopy as the initial diagnostic test for tuberculosis: a cluster-randomised trial embedded in South African roll-out of Xpert MTB/RIF |
| Year | 2015 |
| Language | English |
| Country | South Africa |
| Type | Peer reviewed paper |
| Design | Cluster-randomised trial |
| Intervention | Xpert vs fluorescent microscopy |
| Outcome | Impact |
| Results | Impact= Based on the total cohort of 4656 participants, the 6-month mortality risk was 3.9% (91/2324) and 5.0% (116/2332) in the Xpert and microscopy groups, respectively, giving a risk ratio, adjusted for randomisation stratum only, of 0.86 (95%CI 0.56–1.28; p=0.43). The median time to starting tuberculosis treatment was 7 days in the Xpert group and 10 days in the microscopy group. |

131

| Ref # | 146 |
| --- | --- |
| ID | 3958 |
| Disease | Tuberculosis |
| Authors | Lawn SD, Kerkhoff AD, Vogt M, Wood R. |
| Title | Clinical significance of lipoarabinomannan detection in urine using a low-cost point-of-care diagnostic assay for HIV-associated tuberculosis |
| Year | 2012 |
| Language | English |
| Country | South Africa |
| Type | Peer reviewed paper |
| Design | Cannot tell |
| Intervention | TB LAM |
| Outcome | Impact |
| Results | Had this assay been used in the clinic during initial screening visit, approximately 40% of the sickest patients might have been able to start TB treatment immediately. The chances of survival of those who died without TB treatment may thereby have been improved. |

132

| Ref # | 147 |
| --- | --- |
| ID | 382 |
| Disease | Tuberculosis |
| Authors | Durovni B, Saraceni V, van den Hof S, Trajman A, Cordeiro-Santos M, Cavalcante S, et al. |
| Title | Impact of replacing smear microscopy with Xpert MTB/RIF for diagnosing tuberculosis in Brazil: a stepped-wedge cluster-randomized trial |
| Year | 2014 |
| Language | English |
| Country | Brazil |
| Type | Peer reviewed paper |
| Design | Stepped-Wedge Cluster-Randomized Trial |
| Intervention | One-sample Xpert |
| Outcome | Impact |
| Results | Laboratory-confirmed notifications: Smear microscopy 30.5 (95%CI 24.9-36.1); Xpert 48.7 (95%CI 41.5-55.8) ITT analysis (availability of back-up smear examination): Smear microscopy 30.5% (95%CI 24.9-36.1) and Xpert 51.1% (95%CI 44.0-58.3). Notifications despite negative laboratory result: Smear microscopy 12.1% (95%CI 6.1-18.0) and Xpert 7.3% (95%CI 2.1-12.5).  Notifications with no laboratory test: Smear microscopy 36.9% (95%CI 26.8-47.1) and Xpert 35.8% (95%CI 27.6-43.9). All notifications: Smear microscopy 79.6% (95%CI 65.7-93.4) and Xpert 91.7 (95%CI 80.2-103.2) Positive laboratory examinations: Smear microscopy 41.5% (95%CI 34.1-48.8) and Xpert 66.2% (95%CI 57.3-75.2). Notification rate difference: 18.1% (95%CI 9.4-26.8) ITT analysis (availability of back-up smear examination): 20.6% (95%CI 12.0-29.2) Notifications despite negative laboratory result: -4.8 (95%CI 12.3-2.8). Notifications with no laboratory test: -1.1% (95%CI -13.5-11.2) All notifications: 12.2 (95%CI -5.0-29.3) Positive laboratory examinations: 24.7 (95%CI 13.7-35.8) Notification rate ratio unadjusted 1.59 (95%CI 1.31-1.88) p value <0.001; adjusted 1.59 Laboratory-confirmed notifications. ITT analysis (availability of back-up smear examination): unadjusted 1.67 (95%CI 1.39-1.96) p value <0.001; adjusted 1.68 (95%CI 1.41-1.95) p value <0.001. Notifications despite negative laboratory result: unadjusted 0.61 (95%CI 0.01-1.23) p value 0.206; adjusted 0.52 (95%CI 0.21-0.84) p value 0.004. Notifications with no laboratory test: unadjusted 0.97 (95%CI 0.63-1.30) p value 0.850; adjusted 0.98 (95%CI 0.64-1.32) p value 0.923. All notifications: unadjusted 1.15 (95%CI 0.94-1.37) p value 0.157; adjusted 1.16 (95%CI 0.96-1.37) p value 0.115. Positive laboratory examinations: unadjusted 1.60 (95%CI 1.33-1.86) p value <0.001; adjusted 1.62 (95%CI 1.40-1.84) p value <0.001. |

133

| Ref # | 148 |
| --- | --- |
| ID | 327 |
| Disease | Tuberculosis |
| Authors | Adelman M, Kurbatova E, Wang W, Leonard M, White N, McFarland D, et al. |
| Title | Cost analysis of a nucleic acid amplification test for pulmonary tuberculosis |
| Year | 2014 |
| Language | English |
| Country | The United states of America |
| Type | Conference abstract |
| Design | cost-effectiveness study |
| Intervention | NAAT |
| Reference test | Culture |
| Outcome | Performance and cost |
| Results | Sensitivity= 99.6%. Specificity 99%.  Cost= Average cost per patient tested with the NAAT was $9922 versus $11848 without NAAT; average cost savings were $1925 per AFB+ specimen tested with NAAT. |

134

| Ref # | 149 |
| --- | --- |
| ID | 529 |
| Disease | Tuberculosis |
| Authors | Naidoo P, Dunbar R, du Toit E, van Niekerk M, Squire SB, Beyers N, et al. |
| Title | Comparing laboratory costs of smear/culture and Xpert MTB/RIF-based tuberculosis diagnostic algorithms |
| Year | 2016 |
| Language | English |
| Country | South Africa |
| Type | Peer reviewed paper |
| Design | Cost study |
| Intervention | Xpert MTB/RIF algorithm |
| Outcome | Cost |
| Results | The Xpert cost per test was US$19.03. The largest cost driver was consumables (77%), due mostly to the cost of the Xpert MTB/RIF cartridges. In the smear based algorithm the mean cost per TB case diagnosed was US$48.77 and in the Xpert based algortihm was US$125.32. The total cost per MDR-TB case diagnosed was US$190.14 in the smear-culture based algorithm compared to US$183.86 in the Xpert-based algorithm. |

135

| Ref # | 150 |
| --- | --- |
| ID | 3715 |
| Disease | Tuberculosis |
| Authors | du Toit E, Squire SB, Dunbar R, Machekano R, Madan J, Beyers N, et al. |
| Title | Comparing multidrug-resistant tuberculosis patient costs under molecular diagnostic algorithms in South Africa |
| Year | 2015 |
| Language | English |
| Country | South Africa |
| Type | Peer reviewed paper |
| Design | Cohort |
| Intervention | LPA and Xpert MTB/RIF |
| Outcome | Cost |
| Results | Direct costs (median. IQR) - $US in LPA group (all patients): Transport costs 3.4 [0-6.9]; medical costs 0 [0-18.1]; direct costs 6.7 [1.1-28.2]. In the Xpert group (all patients): Transport costs 1.5 [0-6.5]; medical costs 0 [0-16.0]; direct costs 4.4 [0.0-22.2]. In the LPA group (pre-treatment): Transport costs 3.2 [0-6.9]; medical costs 0 [0-18.1]; direct costs 6.5 [1.1-25.9] In the Xpert group (pre-treatment): Transport costs 1.5 [0-6.5]; medical costs 0 [0-15.7]; direct costs 4.2 [0.0-20.3]. In the LPA group (on first-line anti-tuberculosis treatment): Transport costs 4.5 [0-6.2]; medical costs 0 [0-24.1]; direct costs 27.5 [0.0-30.0]. In the Xpert group (on first-line anti-tuberculosis treatment): Transport costs 3.4 [0-21.7]; medical costs 0 [0-22.9]; direct costs 4.6 [0.0-44.6]. Indirect costs (median. IQR) - $US in the LPA group (all patients): Cost of transport time 12.3 [6.2-29.6]; Cost of time in health facility 23.7 [11.7-64.4]; Indirect costs 40.0 [20.4-105.9]. In the Xpert group (all patients): Cost of transport time 4.6 [2.6-14.3]; Cost of time in health facility 13.4 [8.2-39.0]; Indirect costs 22.1 [11.0-54.5]. In the LPA group (pre-treatment): Cost of transport time 9.9 [5.8-23.2]; Cost of time in health facility 19.9 [8.9-46.1]; Indirect costs 33.7 [17.5-87.1]. In the Xpert group (pre-treatment): Cost of transport time 4.0 [2.5-9.9]; Cost of time in health facility 12.1 [7.3-30.3]; Indirect costs 17.3 [10.9-46.7]. In the LPA group (on first-line anti-tuberculosis treatment): Cost of transport time 54.8 [30.1-91.2]; Cost of time in health facility 86.4 [31.9-117.0]; Indirect costs 164.7 [76.1-234.5]. In the Xpert group (on first-line anti-tuberculosis treatment): Cost of transport time 25.4 [21.6-46.9]; Cost of time in health facility 37.0 [19.1-155.6]; Indirect costs 61.3 [46.7-202.4]. Total patient costs (median. IQR) - $US in the LPA group (all patients): 68.1 [32.0-142.0]. In the Xpert group (all patients): 38.3 [14.1-79.3]. In the LPA group (pre-treatment): 49.8 [23.7-96.4]. In the Xpert group (pre-treatment): 29.0 [12.5-57.6]. In the LPA group (on first-line anti-tuberculosis treatment): 167.6 [105.1-273.2]. In the Xpert group (on first-line anti-tuberculosis treatment): 179.4 [65.8-228.7] |

136

| Ref # | 151 |
| --- | --- |
| ID | 1121 |
| Disease | Tuberculosis |
| Authors | de Camargo KR, Jr., Guedes CR, Caetano R, Menezes A, Trajman A. |
| Title | The adoption of a new diagnostic technology for tuberculosis in two Brazilian cities from the perspective of patients and healthcare workers: a qualitative study |
| Year | 2015 |
| Language | English |
| Country | Brazil |
| Type | Peer reviewed paper |
| Design | Qualitative |
| Intervention | Xpert MTB/RIF |
| Outcome | Acceptability |
| Results | There was no noticeable resistance or difficulty posed by the lab technicians to the new technology. The lab workers reported that the implementation of the new technology has had a very positive impact on the improvement of working conditions in the laboratory. They do not have to constantly deal with fire, nor with foul odors. The ergonomic conditions improved considerably since the technicians do not need to spend their day poring over microscopes. |

137

| Ref # | 152 |
| --- | --- |
| ID | 1691 |
| Disease | Tuberculosis |
| Authors | Telles MAdS, Menezes A, Trajman A |
| Title | Bottlenecks and recommendations for the incorporation of new technologies in the tuberculosis laboratory network in Brazil |
| Year | 2012 |
| Language | English |
| Country | Brazil |
| Type | Peer reviewed paper |
| Design | Retrospective |
| Intervention | Xpert MTB/RIF |
| Outcome | Feasibility |
| Results | Strengths: descentralization, SOP, computerized labs, work organization, QC qualified staff, suitable size. Bottlenecks: QC of periferal network, insufficient visits, low coverage, delay reporting of results, low financial investment, lack of staff replacement program. |

138

| Ref # | 153 |
| --- | --- |
| ID | 3800 |
| Disease | Tuberculosis |
| Authors | Durovni B, Saraceni V, van den Hof S, Trajman A, Cordeiro-Santos M, Cavalcante S, et al. |
| Title | Operational lessons drawn from pilot implementation of Xpert MTB/Rif in Brazil |
| Year | 2014 |
| Language | English |
| Country | Brazil |
| Type | Peer reviewed paper |
| Design | Prospective |
| Intervention | Xpert |
| Outcome | Adoption |
| Results | To minimize errors when using Xpert, notification forms have to be adapted and an efficient communication system has to be in place. A regular supply of PCR cartridges and the ready availability of spare parts must be negotiated with the manufacturer of Xpert. The capacity to perform sputum smear microscopy should be maintained for follow-up tests and for the testing of sputum samples too scanty to run Xpert. |

139

| Ref # | 154 |
| --- | --- |
| ID | 3111 |
| Disease | Tuberculosis |
| Authors | Perkins MD, Conde MB, Martins M, Kritski AL. |
| Title | Serologic diagnosis of tuberculosis using a simple commercial multiantigen assay |
| Year | 2003 |
| Language | English |
| Country | Brazil |
| Type | Peer reviewed paper |
| Design | Cross-sectional |
| Intervention | ICT Tuberculosis |
| Outcome | Performance |
| Reference test | Microscopy and Löwenstein-Jensen culture |
| Results | Sensitivity= Combining acid-fast sputum smear microscopy with serology (in HIV-uninfected TB patients): 77.1% which was equivalent to culture (p = 0.80).  Combined sensitivity of microscopy plus serology was 37.9% in HIV-coinfected patients and 72.4% overall.  Specificity=Comparing Xpert to a gold standard o culture or histology: 100% (95%CI 95.7-100) TB culture compared with histology confirmed TB: 100% (95%CI 95.7-100) Smear compared with culture or histology: 98.8% (95%CI 93.6-99.9) Evidence of acid-fast bacilli on Ziehl-Nielsen stain only: 100.0% (95%CI 95.4-100.0) Histology: 100% (95%CI 95.5-100.0)  Other=PPV100% (95%CI 81.6-100) Evidence of acid-fast bacilli on Ziehl-Nielsen stain only: 100.0% (95%CI 79.6-100.0) Histology: 100% (95%CI 85.7-100.0) NPV:  93.5% (95%CI 86.3-97.6) Evidence of acid-fast bacilli on Ziehl-Nielson stain only: 91.4% (95%CI 83.8-96.2) Histology: 100.0% (95%CI 95.5-100.0) |

140

| Ref # | 155 |
| --- | --- |
| ID | 3158 |
| Disease | Tuberculosis |
| Authors | Gounder C, De Queiroz Mello FC, Conde MB, Bishai WR, Kritski AL, Chaisson RE, et al. |
| Title | Field evaluation of a rapid immunochromatographic test for tuberculosis |
| Year | 2002 |
| Language | English |
| Country | Brazil |
| Type | Peer reviewed paper |
| Design | Case-control study |
| Intervention | ICT1 and ICT 2 |
| Reference test | Löwenstein-Jensen culture |
| Outcome | Performance |
| Results | Sensitivity ICT-1: using serum: 65% (95%CI 41–85), using plasma: 70% (95%CI 6–88) and using whole blood: 83% (95%CI 59–96). ICT 2 using serum: 70 (95%CI 46–88) Specificity= Among the controls, the specificities ranged from 71 to 100%, while among patients suspected of having PTB the specificities were poorer, ranging from 46 to 67%. |

141

| Ref # | 156 |
| --- | --- |
| ID | 4607 |
| Disease | Tuberculosis |
| Authors | Dumaplin D, Ferrer M, Divinagracia EM, Cabana R, Sirilan L, Guzman-Trivilegio R, et al. |
| Title | Clinical evaluation of a lateral flow serologic test in the rapid diagnosis of pulmonary TB in a public-private mix for dots setting in iloilo city, philippines |
| Year | 2010 |
| Language | English |
| Country | The Philippines |
| Type | Conference abstract |
| Design | Case-control |
| Intervention | TB STAT PAK II assay |
| Reference test | Cannot tell |
| Outcome | Performance |
| Results | Sensitivity= 63.4%. Specificity=100%. PPV and NPV was 100% and 53.44%, respectively. |

142

| Ref # | 157 |
| --- | --- |
| ID | 3588 |
| Disease | Tuberculosis |
| Authors | Dumaplin D, Ferrer M, Divinagracia EM, Sirilan L, Cabana R, Guzman-Trivilegio R, et al. |
| Title | Clinical utility of a lateral flow serologic test in the rapid diagnosis of pulmonary TB in a public-private mix for DOTS setting in Iloilo City, Philippines |
| Year | 2011 |
| Language | English |
| Country | Philippines |
| Type | Conference abstract |
| Design | Case-control study |
| Intervention | TB STAT PAK II assay |
| Reference test | Cannot tell |
| Outcome | Performance |
| Results | Sensitivity= 65%. Specificity=100%. PPV and NPV was 100% and 50.94%, respectively. |

143

| Ref # | 158 |
| --- | --- |
| ID | 7118 |
| Disease | Tuberculosis |
| Authors | García-Cruz AE, Olvera-Castillo R, Hernández-Zarza NM, Antuna-Puente B, Uribe-Campero L, Rivas-Ruiz R, et al. |
| Title | Diagnosis of pulmonary tuberculosis by rapid immunochromatography |
| Year | 2011 |
| Language | Spanish |
| Country | Mexico |
| Type | Peer reviewed paper |
| Design | Cross-sectional |
| Intervention | PRIM |
| Reference test | Culture and clinical and PPD and CXR (Class 3 American Thorax Society) |
| Outcome | Performance |
| Results | Sensitivity= 79.2% (95%CI 67.2-87.5) Specificity= 100% (95%CI 93.6-100) PPV: 100% (95%CI 92.1-100) NPV: 82.6% (95%CI 72.5-89.6) LR+: 563 (95%CI 1.15-4000.6) LR-: 0.21 (0.13 a 0.33) The concordance (kappa) of the reading of the result (positive or negative) between the two PRIM observers was 0.83. |

144

| Ref # | 159 |
| --- | --- |
| ID | 6948 |
| Disease | Tuberculosis |
| Authors | Al-Jebouri MM, Wahid NM. |
| Title | An Evaluation of QuantiFERON-TB Gold in-Tube and Immunological Tests for TB Diagnosis in Iraqi Patients |
| Year | 2014 |
| Language | English |
| Country | Iraq |
| Type | Peer reviewed paper |
| Design | Prospective |
| Intervention | OnSite TB IgM/IgG |
| Reference test | QuantiFERON Gold In-Tube |
| Outcome | Performance |
| Results | Sensitivity= 88% Specificity=55% |

145

| Ref # | 160 |
| --- | --- |
| ID | 2569 |
| Disease | Tuberculosis |
| Authors | Ašćerić M, Nadarević A, Avdić S, Vrabac-Mujčinagić M, Nukić S, Mujčinović Z |
| Title | Hexagon TB fo the rapid diagnosis of lung TBC in praxis. |
| Year | 2007 |
| Language | English |
| Country | Boznia and Herzegovina |
| Type | Peer reviewed paper |
| Design | Cross-sectional |
| Intervention | Hexagon TB for diagnosis of pulmonary tuberculosis compared with X-ray, BK and culture |
| Reference test | Chest X-Ray, microscopy and LöW culture |
| Outcome | Performance |
| Results | 11 patients had positive Hexagon TB, 10 had changes in X-ray, 3 culture positive, and 1 BK positive. |

146

| Ref # | 161 |
| --- | --- |
| ID | 4088 |
| Disease | Tuberculosis |
| Authors | Van Beek SC, Nhung NV, Sy DN, Sterk PJ, Tiemersma EW, Cobelens FGJ |
| Title | Measurement of exhaled nitric oxide as a potential screeningtool for pulmonary tuberculosis |
| Year | 2011 |
| Language | English |
| Country | Vietnam |
| Type | Peer reviewed paper |
| Design | Cross-sectional |
| Intervention | Exhaled nitric oxide (eNO) analyser |
| Reference test | Löwenstein-Jensen culture |
| Outcome | Performance |
| Results | Sensitivity= 77.8% (95% CI 67.8–85.9)  Specificity= 61.5% (95%CI 47.0–74.7)  When comparing TB patients with the combined comparison groups (area under ROC curve [AUC] 0.568), no optimal cut-off value could be identified. When comparing TB patients with hospital workers, the diagnostic performance was better AUC 0.723 with an optimum cut-off value of 10ppb |

147

| Ref # | 162 |
| --- | --- |
| ID | 669 |
| Disease | Tuberculosis |
| Authors | Barreto LBPF, Lourenço MCS, Rolla VC, Veloso VG, Huf G |
| Title | Use of amplified Mycobacterium tuberculosis direct test in respiratory samples from HIV-infected patients in Brazil |
| Year | 2014 |
| Language | English |
| Country | Brazil |
| Type | Peer reviewed paper |
| Design | Prospective |
| Intervention | AMTD compared with culture |
| Reference test | Löwenstein-Jensen and BACTEC MGIT 960 cultures |
| Outcome | Performance |
| Results | Sensitivity=AMTD compared with LJ = 87.5% (95%CI 71.0-96.5). AMTD compared with MGIT = 88.6% (95%CI 73.3-96.8). In Smear negative patients =70.8% (95%CI 48.6-87.3)  Specificity= AMTD compared with LJ =89.4% (95%CI 80.8-95.0) AMTD compared with MGIT =92.4% (95%CI 84.2-97.2) In Smear negative patients = 94.8% (95% CI 87.2-98.6)  PPV= AMTD compared with LJ =75.7% (95%CI 58.8-88.2) AMTD compared with MGIT =83.8% (95%CI 68.0-93.8) In Smear negative patients = 81.0% (95%CI 58.1-94.6). NPV= AMTD compared with LJ =95.0% (95%CI 87.7-98.6). AMTD compared with MGIT =94.8% (95%CI 87.2-98.6). In Smear negative patients =91.3% (95%CI 82.8-96.4). LR+: AMTD compared with LJ =8.25 (95%CI 4.39-15.54). AMTD compared with MGIT =11.66 (95%CI 5.35-25.40). LR- AMTD compared with LJ =0.14 (95%CI 0.06-0.35). AMTD compared with MGIT =0.12 (95%CI 0.05-0.31). |

148

| Ref # | 163 |
| --- | --- |
| ID | 1508 |
| Disease | Visceral leishmaniasis |
| Authors | Moura AS, Lopes HM, Mourão MV, Morais MH. |
| Title | Performance of a rapid diagnostic test for the detection of visceral leishmaniasis in a large urban setting |
| Year | 2013 |
| Language | English |
| Country | Brazil |
| Type | Peer reviewed paper |
| Design | Cannot tell |
| Intervention | rK39 RDT |
| Reference test | Ministry of Health definition of confirmed VL: individuals from an area of occurrence of VL with fever and spleen enlargement I) in whom the parasite is identified in direct examination or culture and/or II) demonstrate a positive IFI reaction (titer equal or greater than 1:80) or, although not showing laboratory confi mation, III) demonstrate a favorable treatment response |
| Outcome | Performance |
| Results | Sensitivity= rK39: 72.4% (95%CI 64.6-79%) RDT in HIV-infected patiens: 60% (95%CI 40.7-76.6)  Specificity= rK39: 99.6& (95%CI 97.6-99.9) RDT in HIV-infected patiens: 100% (95%CI 94.7-100)  PPV 99.1% (95%CI 94.8-99.8); NPV 85.5% (80.8-89.1) RDT in HIV-infected patiens: PPV 100% (95%CI 79.6-100); NPV 87.2% (95%CI 77.9-92.9%) |

149

| Ref # | 164 |
| --- | --- |
| ID | 59 |
| Disease | Visceral leishmaniasis |
| Authors | Celeste BJ, Sanchez MCA, Almeida RPD, Castelo Branco CMF, Lindoso JAL, Paniago AMM, et al. |
| Title | Evaluation of immunochromatographic assay with recombinant antigen K39, using whole blood, serum and oral fluid, in the diagnosis of visceral leishmaniasis in Brazilian endemic areas |
| Year | 2015 |
| Language | English |
| Country | Brazil |
| Type | Conference abstract |
| Design | Case-control study |
| Intervention | RDT with rk39, IFAT and ELISA |
| Reference test | Parasitology and/or Direct agglutination test ≥3200 |
| Outcome | Performance |
| Results | Sensitivity In whole blood and serum = 87.59% Oral fluid=71.72% Specificity In Whole blood=100% |

150

| Ref # | 165 |
| --- | --- |
| ID | 224 |
| Disease | Visceral leishmaniasis |
| Authors | Alcoba G, Atia AA, Mumina A, Sterk E, Antierens A, Chappuis F |
| Title | Impact of pediatric and adult acute malnutrition on visceral leishmaniasis RK39 diagnostic test results and clinical outcome in the sudan |
| Year | 2014 |
| Language | English |
| Country | Sudan |
| Type | Conference abstract |
| Design | Cannot tell |
| Intervention | rK39 RDT |
| Reference test | Direct agglutination test or lymph node aspirate |
| Outcome | Performance |
| Results | No significant rK39 false-negative difference was detected in malnourished patient. 8.4% with negative rK30 RDT was confirmed by direct agglutination test or lymph biopsy.  For ages 6-59 months rK39 was negative in 7.4% of GAM and 8.6% non-GAM children (p=0.78). For adults, rK39 was negative in 11.20% of GAM vs. 8.48% of non-GAM (p=0.37) |

151

| Ref # | 166 |
| --- | --- |
| ID | 2692 |
| Disease | Visceral leishmaniasis |
| Authors | Moreno EC, Melo MN, Lambertucci JR, Serufo JC, Andrade ASR, Antunes CMF, Genaro O, Carneiro M |
| Title | Diagnosing human asymptomatic visceral leishmaniasis in an urban area of the State of Minas Gerais, using serological and molecular biology techniques |
| Year | 2006 |
| Language | English |
| Country | Brazil |
| Type | Peer reviewed paper |
| Design | Cross-sectional |
| Intervention | rk39-ELISA and strip test |
| Reference test | IFAT and ELISA with crude antigen from *L. amazonensis* |
| Outcome | Performance |
| Results | Sensitivity= IFAT: 30.1% (95%CI 22.4-38.6) ELISA: 24.8% (95%CI 17.7-33.0) rk39-ELISA: 26.3% (95%CI 19.1-34.7) Serological tests: 55.6% (95%CI 46.8-64.3)  Specificity= IFAT: 63.4% (95%CI 52.8-73.2) ELISA: 71.0% (95%CI 52.8-73.2) rk39-ELISA: 76.3% (95%CI 66.4-84.5) Serological tests: 39.8% (95%CI 29.8-50.5)  Kappa= IFAT 0.74 (95%CI 0.40-1.0) ELISA 0.53 (95%CI 0.18-0.89) Strip test 0.14 (95%CI -0.11-0.40) PCR test 0.89 (95%CI 0.67-1.0) Pearsons’ correlation for: ELISA absorbency r=0.63 IFAT titers r=0.76 |

152

| Ref # | 167 |
| --- | --- |
| ID | 3972 |
| Disease | Visceral leishmaniasis |
| Authors | Goswami RP, Goswami RP, Das S, Ray Y, Rahman M. |
| Title | Testing urine samples with rK39 strip as the simplest non-invasive field diagnosis for visceral leishmaniasis: An early report from eastern India |
| Year | 2012 |
| Language | English |
| Country | India |
| Type | Peer reviewed paper |
| Design | Case-control study |
| Intervention | Kalazar detect using blood and urine |
| Reference test | VL with parasitology of spleen or bone-marrow aspirate and slit-slin smear or skin biopsy for post-Kala azar dermal leishmaniasis |
| Outcome | Performance |
| Results | Sensitivity=rk39 strip test with urine: 100% (95%CI 94.95-100). Wit blood: 100% (95%CI 94.95-100) Specificity=rk39 strip test with urine: 86.33% (95%CI 79.23-91.36). With blood: 92.08% (95%CI 85.95-95.78) |

153

| Ref # | 168 |
| --- | --- |
| ID | 4228 |
| Disease | Visceral leishmaniasis |
| Authors | Gavgani AM, Khademvatan S, Ghazanchaei A. |
| Title | Katex antigen-detection test as a diagnostic tool for latent visceral leishmanisis cases |
| Year | 2008 |
| Language | English |
| Country | Iran |
| Type | Peer reviewed paper |
| Design | Prospective |
| Intervention | Katex |
| Reference test | ELISA IgM, ELISA IgG, DAT and IFA |
| Outcome | Performance |
| Results | Sensitivity: Katex 77.7%, IFA 83.3%, DAT 88.8%, ELISA IgG 94.4% and ELISA IgM 72.2%.  Specificity: Katex 98.2%, IFA 92.9%, DAT 91.2%, ELISA IgG 87.7% and ELISA IgM 89%.  PPV: Katex 93.3%, IFA 78.9%, DAT 76%, ELISA IgG 70.8% and ELISA IgM 68.4%; NPV: Katex 93.3%, IFA 94.6%, DAT 96.2%, ELISA IgG 98%, ELISA IgM 91.4%; efficiency: Katex 95.5, IFA 90.6, DAT 90.6, ELISA IgG 89, ELISA IgM 85.3 |

154

| Ref # | 169 |
| --- | --- |
| ID | 28 |
| Disease | Visceral leishmaniasis |
| Authors | Assis TS, Guimarães PN, Oliveira E, Peruhype-Magalhães V, Gomes LI, Rabello A. |
| Title | Study of implementation and direct cost estimates for diagnostic tests for human visceral leishmaniasis in an urban area in Brazil |
| Year | 2007 |
| Language | English |
| Country | Brazil |
| Type | Peer reviewed paper |
| Design | Prospective |
| Intervention | Implementig diagnostic tests IT-LEISH and the DAT-LPC for the diagnosis of visceral leishmaniasis. |
| Outcome | Cost |
| Results | The cost to train each health professional was estimated at US$ 7.13 for the implementation of the IT LEISH and US$ 9.93 for the implementation of the DAT-LPC. The direct cost and performance time of the tests in Ribeirão das Neves were respectively estimated to be US$ 6.62 and 30 minutes for the IT LEISH and US$ 5.44 and 50 minutes for the DAT-LPC. For the other diagnostic tests costs were: Kala-Azar Detect rapid test –US$ 6.72 and 60 minutes; bone marrow aspirate – US$ 26.85 and 130 minutes; IFAT – US$ 12.54 and 140 minutes; PCR – US$ 34.02 and 200 minutes |

155

| Ref # | 170 |
| --- | --- |
| ID | 948 |
| Disease | Visceral leishmaniasis |
| Authors | Assis TM, Guimarães PN, Oliveira E, Peruhype-Magalhães V, Gomes LI, Rabello A. |
| Title | Acceptance and potential barriers to effective use of diagnostic tests for visceral leishmaniasis in an urban area in Brazil |
| Year | 2016 |
| Language | English |
| Country | Brazil |
| Type | Peer reviewed paper |
| Design | Prospective |
| Intervention | IT LEISH |
| Outcome | Acceptability |
| Results | 96% of patients considered fingertip blood collection a positive feature of the rapid test. 86% of health care professionals considered the test easy to perform.  All professionals from the Municipal Laboratory  relied on the results of the DAT-LPC; they experienced no difficulty in performing the test, classified the technical complexity of the test as simple and easy to read, and believed that the implementation of the DAT-LPC in the health service could benefit VL patients |

156

| Ref # | 171 |
| --- | --- |
| ID | 3903 |
| Disease | Visceral leishmaniasis |
| Authors | Akhoundi B, Mohebali M, Shojaee S, Jalali M, Kazemi B, Bandehpour M, et al. |
| Title | Rapid detection of human and canine visceral leishmaniasis: Assessement of a latex agglutination test based on the A2 antigen from amastigote forms of Leishmania infantum |
| Year | 2013 |
| Language | English |
| Country | Iran |
| Type | Peer reviewed paper |
| Design | Cross-sectional |
| Intervention | Latex agglutination test (LAT) |
| Reference test | Bone marrow smears and Direct agglutination test (DAT) titre ≥3:200 |
| Outcome | Performance |
| Results | Sensitivity= A2 LAT 88.4%, Pro LAT 88.4%. Specificity= A2 LAT 93.5%, Pro LAT 100% Validity (Sn+Sp)/2 = A2 LAT 90.9, Pro LAT 90.1, and Agreement A2 LAT 0.914, pro LAT 0.904. Reproducibility of LAT 98% in 2 human sera. False positives with both LAT in 1 TB and 1 leprosy patients |

157

| Ref # | 172 |
| --- | --- |
| ID | 2594 |
| Disease | Leptospirosis |
| Authors | McBride AJ, Santos BL, Queiroz A, Santos AC, Hartskeerl RA, Reis MG, et al. |
| Title | Evaluation of four whole-cell Leptospira-based serological tests for diagnosis of urban leptospirosis |
| Year | 2007 |
| Language | English |
| Country | Brazil |
| Type | Peer reviewed paper |
| Design | Cannot tell |
| Intervention | The Leptospira immunoglobulin M (IgM) ELISA, Dip-S-Tick, LeptoTek DriDot, EIE-IgM-Leptospirose (Bio-Manguinhos) |
| Reference test | MAT and culture in paired serum samples |
| Outcome | Performance |
| Result | Sensitivity=Acute phase (n=96) MAT: 68.8% (95%CI 58.4-77.6) ELISA BM: 79.2% (95%CI 69.4-86.5) ELISA PB: 87.5% (95%CI 78.8-93.1) DD: 80.0% (95%CI 70.3-87.2) DS: 72.3% (95%CI 62.0-80.8) Acute phase (n=72) MAT: 45.8% (95%CI 34.2-57.9) ELISA BM: 54.2% (95%CI 42.1-65.8) ELISA PB: 66.7% (95%CI 54.5-77.1) DD: 50.0% (95%CI 37.9-62.1) DS: 32.9% (95%CI 22.4-45.2) Convalescent phase (n=50) MAT: 100.0% (95%CI 91.1-100.0) ELISA BM: 96.0% (95%CI 85.1-99.3) ELISA PB: 92.0% (95%CI 79.9-97.4) DD: 84.0% (95%CI 70.3-92.4) DS: 80.0% (95%CI 65.9-89.5).  Specificity n=80 MAT: 100.0% (95%CI 94.3-100.0) ELISA BM: 95.0% (95%CI 87.0-98.4) ELISA PB: 87.5% (95%CI 77.8-93.5) DD: 95.0% (95%CI 87.0-98.4) DS: 100.0% (95%CI 94.2-100.0) |

158

| Ref # | 173 |
| --- | --- |
| ID | 4483 |
| Disease | Leptospirosis |
| Authors | Eapen CK, Sugathan S, Kuriakose M, Abdoel T, Smits HL. |
| Title | Evaluation of the clinical utility of a rapid blood test for human leptospirosis |
| Year | 2002 |
| Language | English |
| Country | India |
| Type | Peer reviewed paper |
| Design | Cannot tell |
| Intervention | Lateral flow assay |
| Reference test | IgM ELISA titre ≥1:80 |
| Outcome | Performance |
| Result | Sensitivity= Lateral flow assay: 65.4% (collected during the first 10 days after the onset of illness), 80.9% (collected at the later stage) ELISA 56.7% and 84.3%  Specificity Lateral flow assay: 93.6% ELISA: 94.2%  Kappa 0.87 (95%CI 0.74-1) between lateral flow assay and IgM ELISA |

159

| Ref # | 174 |
| --- | --- |
| ID | 5074 |
| Disease | Leptospirosis |
| Authors | Cermeño Vivas JR, Sandoval De Mora M, Bognanno J, Caraballo A. |
| Title | Clinical and epidemiological features of leptospirosis in Bolívar state, Venezuela. Comparison of diagnostic methods: LEPTO-Dipstick and plaque macroscopic agglutination test. |
| Year | 2005 |
| Language | Spanish |
| Country | Venezuela |
| Type | Peer reviewed paper |
| Design | Cross-sectional |
| Intervention | LEPTO-Dipstick and the macroscopic agglutination in plate with the termorresistent antigen |
| Reference test | MAT seroconversión or 4x increased in titre or titre ≥1:200 |
| Outcome | Performance |
| Results | Sensitivity=TR = 86.7%, LEPTO-Dipstick = 93.3%  Specificity= TR= 25%, LEPTO-Dipstick = 25%  PPV TR=52%, LEPTO-Dipstick=53.9%. NPV TR= 66.7%, LEPTO-Dipstick = 80% |

160

| Ref # | 175 |
| --- | --- |
| ID | 2681 |
| Disease | Leptospirosis |
| Authors | Obregon AM, Fernandez C, Rodriguez I, Rodriguez J, Zamora Y. |
| Title | Laboratory advances in serologic diagnosis and research of human leptospirosis in Cuba |
| Year | 2007 |
| Language | Spanish |
| Country | Cuba |
| Type | Peer reviewed paper |
| Design | Descriptive |
| Intervention | Lepto Tek Lateral Flow (LTLF), Lepto Tek Dip Stick (LTDS) and Lepto Tek  Dri-Dot (LTDD) |
| Reference test | Direct Aglutination and MAT |
| Outcome | Performance |
| Results | Sensitivity= LTLF:> 90% LTDS:> 90% LTDD:> 85% Latex-Mixed Conjugate: 93.8%  Specificity Latex-Mixed Conjugate: 90.4%  PPV: Latex-Sejroe conjugate: 90.9% latex-blended conjugate: 94.2% NPV: Latex-Sejroe conjugate: 95.8% latex-blended conjugate: 96.6% |

161

| Ref # | 176 |
| --- | --- |
| ID | 3482 |
| Disease | Leptospirosis |
| Authors | Nabity SA, Ribeiro GS, Takahashi D, Medeiros MA, Lessa C, Damião A, et al. |
| Title | Evaluation of a dual path platform (DPP) assay for the rapid diagnosis of leptospirosis |
| Year | 2011 |
| Language | English |
| Country | Brazil |
| Type | Conference abstract |
| Design | Cannot tell |
| Intervention | Rapid serodiagnostic assay |
| Reference test | Whole leptospira IgM ELISA |
| Outcome | Performance |
| Results | Sensitivity was 85% (95%CI 81-89) and 61% (95%CI 42-78) for acute-phase severe and mild leptospirosis from Salvador, respectively, which was similar to whole-Leptospira IgM ELISA (82% [95%CI 76-86] and 38% [95%CI 18-62], respectively). During the 1st seven days of illness, sensitivity was 77% (95%CI 66-85) and 56% (95%CI 35-75) for severe and mild leptospirosis. In severe disease convalescence, sensitivity was equivalent (98% [95%CI 94-99]) to ELISA (99% [95%CI 95-99]). Sensitivity for acute-phase Curitiba sera (58% [95%CI 46-69]) was similar to ELISA (66% [95%CI 55-77]); whereas it was lower (81% [95%CI 65-91]) than ELISA (100% [95%CI 91-100]) in convalescence.  The specificity was ≥94% for dengue, hepatitis A, syphilis, febrile outpatients, and Salvador blood donors. However, specificity was lower (85%; 95%CI 77-91) for healthy residents of a slum within Salvador with high endemic transmission of leptospirosis. |

162

| Ref # | 177 |
| --- | --- |
| ID | 1126 |
| Disease | Filariasis |
| Authors | Yahathugoda TC, Supali T, Rao RU, Djuardi Y, Stefani D, Pical F, et al. |
| Title | A comparison of two tests for filarial antigenemia in areas in Sri Lanka and Indonesia with low-level persistence of lymphatic filariasis following mass drug administration |
| Year | 2015 |
| Language | English |
| Country | Sri Lanka and Indonesia |
| Type | Peer reviewed paper |
| Design | Cross-sectional |
| Intervention | Alere Filariasis Test Strip (FTS) and Binax Now Filariasis card test (Card test) |
| Reference test | None |
| Outcome | Performance |
| Results | % Agreement: 98%. Kappa: 0.825 (IC95% 0.739–0.912) More FTS turned positive at both time points compared to Card Tests (1.7 % vs. 0.5 % at 30 min, and 3.8 % vs. 1.4 % at 12 h, respectively) |

163

| Ref # | 178 |
| --- | --- |
| ID | 2929 |
| Disease | Filariasis |
| Authors | Jamail M, Andrew K, Junaidi D, Krishnan AK, Faizal M, Rahmah N. |
| Title | Field validation of sensitivity and specificity of rapid test for detection of Brugia malayi infection |
| Year | 2005 |
| Language | English |
| Country | Malaysia |
| Type | Peer reviewed paper |
| Design | Cannot tell |
| Intervention | Complex intervention including health education, training of the staff and use of Brugia rapid |
| Reference test | Microscopy |
| Outcome | Performance, acceptability and appropiateness |
| Results | Sensitivity 87% [(20/23); 95% CI: 66.4–97.2] Specificity 100%  Acceptability= In general the field staff was satisfied with the ease of use and of the test interpretation. One notable advantage of the test, as reported by the field staff, is the increased drug compliance by the villagers as they could themselves visualize the positive dipstick results.  Appropiateness= Besides being a much more sensitive test than TBS in detecting Brugia infection, it does not require night blood sampling and results can be obtained soon after the blood sample is taken. |

164

| Ref # | 179 |
| --- | --- |
| ID | 3679 |
| Disease | Filariasis |
| Authors | Dewi RM, Tuti S, Ganefa S, Anwar C, Larasati R, Ariyanti E, et al |
| Title | Brugia Rapid™ antibody responses in communities of Indonesia in relation to the results of ‘transmission assessment surveys’ (TAS) for the lymphatic filariasis elimination program. |
| Year | 2015 |
| Language | English |
| Country | Indonesia |
| Type | Peer reviewed paper |
| Design | Cross-sectional |
| Intervention | Brugia rapid |
| Reference test | Microscopy |
| Outcome | Performance |
| Results | In the non-endemic site a total of 23 (5%) study participants were antibody positive; however, none was positive for microfilaremia. In the Post-MDA passing TAS site three (1 %) participants were antibody positive, 21 (4%) had invalid Brugia Rapid™ tests, and 490 (95%) were antibody negative; all 514 participants were negative for microfilaremia. In the post-MDA, failing TAS site: 79 (15%) of participants were antibody positive, one (0.2%) had an invalid Brugia Rapid™ test, and 443 (85%) were antibody negative. One participant was positive for B. malayi microfilaremia as well as antibodies. |

165

| Ref # | 180 |
| --- | --- |
| ID | 5720 |
| Disease | Filariasis |
| Authors | Oliveira CMd |
| Title | Validação dos anticorpos monoclonais Og4C3 e AD12 no diagnóstico da filariose bancroftiana em inquérito populacional |
| Year | 2003 |
| Language | Portuguese |
| Country | Brazil |
| Type | Thesis |
| Design | Diagnostic evaluation study |
| Intervention | ELISA - Og4C3 and ICT |
| Reference test | Microscopy |
| Outcome | Performance |
| Results | Sensitivity= ELISA-Og4C3 vs Thick smear: 96.1% (95%CI 91.8-98.3) TIC vs Thick smear: 94.4% (95%CI 89.7-97.2)  Specificity= ELISA-Og4C3 vs Thick smear: 85.3% (95%CI 82.2-88.0) TIC vs Thick smear: 90.7% (95%CI 87.2-93.4)  ELISA-Og4C3 vs Thick smear: PPV 65.9% (95%CI 59.8-71.6); NPV 98.7% (95%CI 97.1-99.4); accuracy 87.7 TIC vs Thick smear: PPV 83.3% (95%CI 77.3-88.0); NPV 97.1% (95%CI 94.5-98.5); accuracy 91.9% |

166

| Ref # | 181 |
| --- | --- |
| ID | 5743 |
| Disease | Filariasis |
| Authors | Oliveira PAdS |
| Title | Avalidação da acurácia de diferentes testes laboratorias no diagnóstico da filariose em crianças e adolescentes |
| Year | 2010 |
| Language | Portuguese |
| Country | Brazil |
| Type | Thesis |
| Design | Cross-sectional |
| Intervention | parasitological methods and filarial antigens |
| Reference test | Microscopy |
| Outcome | Performance |
| Results | Sensitivity= Thick drop: 85.2 (95%CI 65.4-95.1) Knott: 85.2% (95%CI 65.4-95.1) ICT: 100% (95%CI 84.5-100) Og4C3: 100% (95%CI 84.5-100)  Specificity Thick drop: 99.8% (95%CI 98.7-100) Knott: 99.6% (95%CI 98.3-99.9) ICT: 84.4% (95%CI 80.8-87.4) Og4C3: 86.5% (95%CI 83.0-89.3)  Thick drop: PPV 95.8 (95%CI 76.9-99.8); NPV 99.2% (95%CI 97.7-99.7); Accuracy 99.0% (95%CI 97.6-99.6); LR+ 0.88; LR- 0.99 Knott: PPV 92.0% (95%CI 72.5-98.6); NPV 99.2% (95%CI 97.7-99.7); Accuracy 98.8% (95%CI 97.3-99.5); LR+ 0.92; LR- 0.99 ICT: PPV 26.2% (95%CI 18.3-36.0); NPV 100% (95%CI 98.8-100); Accuracy 85.2% (95%CI 81.8-88.1); LR+ 3.81; LR- 1.18 Og4C3: PPV 29.0% (95%CI 20.3-39.5); NPV 100% (95%CI 98.9-100); Accuracy 87.2% (95%CI 83.9-89.9); LR+ 3.44; LR- 1.16 |

167

| Ref # | 182 |
| --- | --- |
| ID | 2676 |
| Disease | Enteric fever |
| Authors | Dong B, Galindo CM, Shin E, Acosta CJ, Page AL, Wang M, et al. |
| Title | Optimizing typhoid fever case definitions by combining serological tests in a large population study in Hechi City, China |
| Year | 2007 |
| Language | English |
| Country | China |
| Type | Peer reviewed paper |
| Design | Prospective |
| Intervention | Widal, IDL-Tubex® and Typhidot-M® tests |
| Reference test | Combination of the three tests |
| Outcome | Performance |
| Results | Sensitivity= Tubex1: Tx≥2: 100%, Tx≥4: 69%, Tx≥6: 62% Tx≥8: 23% Tx≥10: 15% Typhidot-M1: Td=positive: 54%  Specificity= Tubex1: Tx≥2: 43% Tx≥4: 95% Tx≥6: 95% Tx≥8: 100% Tx≥10: 100% Typhidot-M1: Td=positive: 91% |

168

| Ref # | 183 |
| --- | --- |
| ID | 4319 |
| Disease | Enteric fever |
| Authors | Dutta S, Sur D, Manna B, Sen B, Deb AK, Deen JL, et al. |
| Title | Evaluation of new-generation serologic tests for the diagnosis of typhoid fever: data from a community-based surveillance in Calcutta, India |
| Year | 2006 |
| Language | English |
| Country | India |
| Type | Peer reviewed paper |
| Design | Cannot tell |
| Intervention | Widal, Typhidot and Tubex test |
| Reference test | Culture |
| Outcome | Performance |
| Results | Sensitivity= Tubex 0.56 (95%CI 0.47-0.66) FEVER OF ≤ 5 DAYS: Typhidot 0.41 (95%CI 0.29-0.54) Tubex 0.57 (95%CI 0.46-0.69) FEVER OF > 5 DAYS: Typhidot 0.59 (95%CI 0.41-0.77) Tubex 0.55 (95%CI 0.38-0.72)  Specificity= Typhidot 0.83 (95%CI 0.71-0.94) Tubex 0.88 (95%CI 0.82-0.94) FEVER OF ≤ 5 DAYS: Typhidot 0.84 (95%CI 0.72-0.97) Tubex 0.91 (95%CI 0.84-0.97) FEVER OF > 5 DAYS: Typhidot 0.75 (95%CI 0.45-1.05) Tubex 0.81 (95%CI 0.68-0.93)  PPV: Typhidot 0.85; Tubex 0.81 FEVER OF ≤ 5 DAYS: Typhidot 0.83; Tubex 0.85 fever of > 5 days: Typhidot 0.89; Tubex 0.72 NPV: Typhidot 0.42; Tubex 0.69 fever of ≤ 5 days: Typhidot 0.44; Tubex 0.70 fever of > 5 days: Typhidot 0.33; Tubex 0.66 |

169

| Ref # | 184 |
| --- | --- |
| ID | 7276 |
| Disease | Enteric fever |
| Authors | Tanyigna KB, Ogor JO. |
| Title | The sensitivity of Diazo test in the diagnosis of enteric fevers |
| Year | 2008 |
| Language | English |
| Country | Nigeria |
| Type | Peer reviewed paper |
| Design | Cross-sectional |
| Intervention | Diazo |
| Reference test | Widal |
| Outcome | Performance |
| Results | Sensitivity= 27.3% |

170

| Ref # | 185 |
| --- | --- |
| ID | 1424 |
| Disease | Schistosomiasis |
| Authors | Adriko M, Standley CJ, Tinkitina B, Tukahebwa EM, Fenwick A, Fleming FM, et al. |
| Title | Evaluation of circulating cathodic antigen (CCA) urine-cassette assayas a survey tool for Schistosoma mansoni in different transmission settings within Bugiri District, Uganda |
| Year | 2014 |
| Language | English |
| Country | Uganda |
| Type | Peer reviewed paper |
| Design | Cross-sectional |
| Intervention | CCA1 and CCA2 |
| Reference test | Six Kato-Katz |
| Outcome | Performance |
| Results | Sensitivity= CCA1 88% (95%CI 82-93), CCA2 59% (95%CI 51-66)  Specificity= CCA1 52% (95%CI 46-58), CCA2 91% (95%CI 87-94%)  PPV CCA1 53% (95%CI 47-58%), CCA2 79% (95%CI 71-86%). NPV CCA1 88% (95%CI 82-92%), CCA2 79% (95%CI 74-83%). AUC CCA1=0.7, CCA2 =0.75 |

171

| Ref # | 186 |
| --- | --- |
| ID | 1014 |
| Disease | Schistosomiasis |
| Authors | Silveira AM, Costa EG, Ray D, Suzuki BM, Hsieh MH, Fraga LA, et al. |
| Title | Evaluation of the CCA Immuno-Chromatographic Test to Diagnose Schistosoma mansoni in Minas Gerais State, Brazil |
| Year | 2016 |
| Language | English |
| Country | Brazil |
| Type | Peer reviewed paper |
| Design | Cross-sectional |
| Intervention | CCA-ICT |
| Reference test | Kato-Katz and ELISA |
| Outcome | Performance |
| Results | Sensitivity= compared with Kato-katz when trace results were considered negative: 68.7% (95%CI 53.7-81.3). When Trace results were considered positive: 85.4% (95%CI 72.2-93.9)  Specificity= compared with Kato-katz when trace results were considered negative: 97.6% (95%CI 87.1-99.6). When Trace results were considered positive: 78% (95%CI 62.4-89.4)  ROC indicated that the trace score should be considered negative in order to segregate the “2KK-NEG non-endemic area” from the “2KK-POS endemic area”. PPV: 97.1% (95%CI 84.7-99.9). NPV: 72.7% (95%CI 59-83.9). AUC: 0.832. The AUC values of SEA-ELISA (AUC: 0.744 (95%CI 0.654–0.834))and SWAP-ELISA (0.704 (95%CI 0.615–0.793)) were lower that obtained with the CCA-ICT |

172

| Ref # | 187 |
| --- | --- |
| ID | 1682 |
| Disease | Schistosomiasis |
| Authors | Sheele JM, Kihara JH, Baddorf S, Byrne J, Ravi B |
| Title | Evaluation of a novel rapid diagnostic test for Schistosoma haematobium based on the detection of human immunoglobulins bound to filtered Schistosoma haematobium eggs |
| Year | 2013 |
| Language | English |
| Country | Kenya |
| Type | Peer reviewed paper |
| Design | Cross-sectional |
| Intervention | RDTSh (Anti-human IgG conjugated to horseradish peroxidase and microscopy |
| Reference test | Urine microscopy |
| Outcome | Performance |
| Results | Sensitivity= 97% (95%CI 91–100) Specificity= 78% (95%CI 67–89)  The RDT-Sh was positive in 46% (6/13) of urine samples containing 0.5–1 egg/10 ml urine, in 89% of urine samples containing >1 egg/10 ml (58/65 samples) and in 97% of urine samples containing >11 eggs/10 ml urine (35/36 samples) |

173

| Ref # | 188 |
| --- | --- |
| ID | 3633 |
| Disease | Dengue |
| Authors | Shih HI, Hsu HC, Wu CJ, Lin CH, Chang CM, Tu YF, et al. |
| Title | Applications of a Rapid and Sensitive Dengue DUO Rapid Immunochromatographic Test Kit as a Diagnostic Strategy during a Dengue Type 2 Epidemic in an Urban City |
| Year | 2016 |
| Language | English |
| Country | Taiwan |
| Type | Peer reviewed paper |
| Design | Prospective observational study |
| Intervention | NS1 and IgM/IgG |
| Reference test | RT-PCR |
| Outcome | Performance |
| Results | Sensitivity= NS1: day 0 of fever 80.65% (95%CI 62.53 - 92.55). Day 1: 95.90% (95%CI 93.11 - 98.68). Day 2: 95.4% (95%CI 91 - 99.8%). Day 3: 97.22% (95%CI 91.85 - 100). Day 4 and 5: 100%. IgM: day 0: 35.48% (95%CI 19.23-54.63). Day 1: 4.62% (95%CI 1.67 - 7.56). Day 2: 2.3% (95%CI 0.00 - 5.45). Day 3: 8.33% (95%CI 0.00 - 17.36). Day 4: 33.33% (95%CI 9.92 - 65.11). Day 5: 88.89% (95%CI 51.75 - 99.72). NS1&IgM: Day 0: 90.32% (95%CI 62.53 - 92.55). Day 1: 95.9 (95%CI 93.11 - 98.68). Day 2: 95.4% (95%CI 91.00 - 99.8). Day 3: 97.22% (95%CI 91.85 - 100). Day 4 and 5: 100%.  Specificity= NS1: day 0 of fever 60.87% (95%CI 38.54 - 80.29). Day 1: 84.13% (95%CI 75.1 - 93.15). Day 2: 76% (95%CI 54.87 - 90.64%). Day 3: 58.82% (95%CI 54.87-90.64). Day 4: 50% (95%CI 11.81 - 88.19). Day 5: 14.29% (95%CI 0.36- 57.87). IgM: day 0: 69.57% (95%CI 47.08 - 86.02). Day 1: 76.19% (95%CI 63.79 - 86.02). Day 2: 64% (95%CI 42.52 - 82.03). Day 3: 52.94% (95%CI 27.81 - 77.02). Day 4: 50% (95%CI 11.81 - 88.19). Day 5: 14.29% (95%CI 0.36 - 57.87). NS1&IgM: Day 0: 56.52% (95%CI 34.49 - 76.81). Day 1: 74.6% (95%CI 62.06 - 84.73). Day 2: 60% (95%CI 38.67 - 78-87). Day 3: 47.06% (95%CI 22.98 - 72.19). Day 4: 50% (95%CI 11.81 - 88.19) Day 5: 14.29% (95%CI 0.36 - 57.87).  PPV NS1: day 0 of fever 73.53 % (95%CI 55.64 - 87.12). Day 1: 94.92% (95%CI 91.86 - 97.99). Day 2: 93.26% (95%CI 88.05 - 98.47%). Day 3: 83.33 (95%CI 72.06 - 96.6). Day 4: 0% (95%CI 51.91 -95.67). Day 5: 60% (95%CI 32.29 - 83.66). IgM: day 0; 61.11% (95%CI 37.75 - 82.7). Day 1: 37.5% (95%CI 18.8 - 59.41). Day 2: 18.18% (95%CI 2.28 - 51.78). Day 3: 27.27% (95%CI 6.02 - 60.97). Day 4: 57.14% (95%CI 18.41- 90.10). Day 5: 60% (95%CI 32.29 - 83.66). NS1&IgM: Day 0: 73.68% (95%CI 59.68 - 87.68). Day 1: 94.92% (95%CI 91.86 - 97.99). Day 2: 89.25% (95%CI 82.95 -95.54). Day 3: 79.55% (95%CI 64.7 - 90.20). Day 4: 80% (95%CI 51.91-95.67) Day 5: 60% (95%CI 32.2-93.66)  NPV NS1: day 0 of fever 70% (95%CI 49.92 - 90.08). Day 1: 86.89% (95%CI 78.41. - 95.36). Day 2: 82.61% (95%CI 61.22 - 95.05). Day 3: 90.91% (95%CI 58.72 - 99.77). Day 4: and Day 5: 100%. IgM: day 0: 44.44% (95%CI 27.94 - 61.90). Day 1: 20.51% (95%CI 15.34 - 25.69). Day 2: 15.84% (95%CI 8.72 -22.96). Day 3: 21.43% (95%CI 10.30 - 36.81). Day 4: 27.27% (95%CI 6.02 - 60.97). Day 5: 50% (95%CI 1.26-98.74). NS1&IgM: Day 0: 81.25% (95%CI 54.35 - 95.95). Day 1: 85.45% (95%CI 76.14 - 94.77). Day 2: 78.95% (95%CI 54.43 -93.95). Day 3: 88.89% (95%CI 51.75 - 99.72). Day 4 and 5: 100% |

174

| Ref # | 189 |
| --- | --- |
| ID | 6812 |
| Disease | Dengue |
| Authors | Alzahrani A. |
| Title | Knowledge and practice of primary health-care physicians regarding the dengue fever in Makkah Al-Mokarramah city, 2013 |
| Year | 2014 |
| Language | English |
| Country | Saudi Arabia |
| Type | Peer reviewed paper |
| Design | Cross-sectional |
| Intervention | RDT/ testing procedures and performance, supply chain management, QA/ QC, staff training, documentation, and storage and waste management |
| Reference test | Cannot tell |
| Outcome | Performance |
| Results | 44% (7/16) of health facilities scored ≤60% for testing performance and only 1 of 16 health facilities achieved 90% |

175

| Ref # | 190 |
| --- | --- |
| ID | 5360 |
| Disease | Leprosy |
| Authors | Grossi MAdF, Leboeuf MAA, Andrade ARCd, Lyon S, Antunes CMdF, Bührer-Sékula S. |
| Title | The influence of ML Flow test in leprosy classification |
| Year | 2008 |
| Language | English |
| Country | Brazil |
| Type | Peer reviewed paper |
| Design | Descriptive exploratory |
| Intervention | ML flow test |
| Reference test | Microscopy and clinical classification |
| Outcome | Impact and performance |
| Results | Impact= Reduction in the percentage of multibacillary (MB) patients among the total of new cases diagnosed in Minas Gerais was observed, falling from 78.1% in 2000, to 65.8%, in March 2004. The reduction in MB cases was greater in health services that participated in the ML Flow study, decreasing from 73.1 to 53.3%, than in the non-participating services, which showed a decrease from 80.6 to 72.2%  Agreement= between ML Flow and classification by skin lesion counts and bacilloscopy was moderate (Kappa: 0.51 and Kappa: 0.48, respectively), but substantial for final classification given by the health center for treatment purposes. The agreement between the first reading of the ML Flow test, as recorded by the local health professionals involved in the study, and the second reading taken by a single, independent examiner in Belo Horizonte was nearly perfect (kappa: 0.81) with 91% agreement in the results.(Kappa: 0.77) |

176

| Ref # | 191 |
| --- | --- |
| ID | 6656 |
| Disease | Leprosy |
| Authors | Duthie MS, Orcullo FM, Maghanoy A, Balagon MF |
| Title | Need for, and acceptability of, rapid diagnostic tests that can facilitate the diagnosis of leprosy |
| Year | 2016 |
| Language | English |
| Country | Philippines |
| Type | Peer reviewed paper |
| Design | Cannot tell |
| Intervention | RDTs |
| Outcome | Acceptability |
| Results | Thought it would be beneficial to have a simple and rapid diagnostic and treatment monitoring test for leprosy: 95.9% patients, 93.2% HHC, 81.4% of the general population responding ‘yes’, 5 patients that did not respond ‘yes’ all responded ‘probably’. No patient responded negatively to the potential use of RDT, 1.9% and 1.3% of HHC and the general population responded ‘no’. The subjects’ perspectives on the extent of benefit associated with RDT: 87.7% answered that RDT use would be very beneficial with 9.9% and 2.5% thinking that it would be moderately and slightly beneficial, respectively. If they would personally submit to testing: 88.6% of the patient population with strongest indication of ‘definitely’ followed by 69.4% of HHC and 72.2% of EC. |

177

| Ref # | 192 |
| --- | --- |
| ID | 1319 |
| Disease | Chagas disease |
| Authors | Shah V, Ferrufino L, Gilman RH, Ramirez M, Saenza E, Malaga E, et al. |
| Title | Field evaluation of the InBios Chagas Detect Plus rapid test in serum and whole blood specimens in Bolivia |
| Year | 2014 |
| Language | English |
| Country | Bolivia |
| Type | Peer reviewed paper |
| Design | Cross-sectional |
| Intervention | Chagas Detect Plus (CDP) compared with conventional serological assays |
| Reference test | Combination of Indirect Hemagluttination Assay (IHA) titre ≥1:16, and immunofluorescent antibody assay, and ELISAs (cutoff 0.300 OD) above of negative controls |
| Outcome | Performance |
| Results | Sensitivity= CDP in whole blood: 96.2%. CDP in serum: 99.3%  Specificity= CDP in whole blood: 98.8%. CDP in serum: 96.9%  Kappa in whole blood: 0.962 (95%CI 0.940-0.984). Kappa in serum: 0.948 (95%CI 0.916-0.98) |

178

| Ref # | 193 |
| --- | --- |
| ID | 84 |
| Disease | Human african trypanosomiasis |
| Authors | Mpanya A, Mbo F, Lumbala C, Hasker E, Ilunga J, Lutumba P, et al |
| Title | Direct comparison of the card agglutination test for trypanosomiasis (CATT) and a rapid diagnostic test in a highly endemic district |
| Year | 2015 |
| Language | English |
| Country | Democratic Republic of Congo |
| Type | Conference abstract |
| Design | Cannot tell |
| Intervention | CATT, RDT SD BIOLINE HAT, and loop mediated isothermal amplification (LAMP) |
| Reference test | Cannot tell |
| Outcome | Performance |
| Results | LAMP results were available for 79 sero-suspects so far and 24 (30%) tested positive. An additional 31 LAMP-positives were identified among seronegative controls. |

179

| Ref # | 194 |
| --- | --- |
| ID | OTHER SOURCES |
| Disease | Cholera |
| Authors | Mukherjee P, Ghosh S, Ramamurthy T, Bhattacharya MK, Nandy RK, Takeda Y, et al. |
| Title | Evaluation of a rapid immunochromatographic dipstick kit for diagnosis cholera emphasizes its outbreak utility. |
| Year | 2010 |
| Language | English |
| Country | India |
| Type | Peer reviewed paper |
| Design | Cannot tell |
| Intervention | Crystal VC |
| Reference test | Culture |
| Outcome | Performance |
| Results | Sensitivity=91.7% Specificity=72.9% PPV= 63.5%. NPV=94.4% |
